# Guest Binding Drives Host Redistribution in Libraries of Co^II^_4_L_4_ Cages

**DOI:** 10.1002/anie.202004627

**Published:** 2020-05-07

**Authors:** Marion Kieffer, Rana A. Bilbeisi, John D. Thoburn, Jack K. Clegg, Jonathan R. Nitschke

**Affiliations:** ^1^ Department of Chemistry University of Cambridge Lensfield Road Cambridge CB2 1EW UK; ^2^ Department of Civil and Environmental Engineering American University of Beirut Beirut Lebanon; ^3^ Department of Chemistry Randolph-Macon College Ashland VA 23005 USA; ^4^ School of Chemistry and Molecular Biosciences The University of Queensland St Lucia QLD 4072 Australia

**Keywords:** host-guest systems, mass spectrometry, metal-organic Cages, self-assembly, supramolecular library

## Abstract

Two Co^II^_4_L_4_ tetrahedral cages prepared from similar building blocks showed contrasting host–guest properties. One cage did not bind guests, whereas the second encapsulated a series of anions, due to electronic and geometric effects. When the building blocks of both cages were present during self‐assembly, a library of five Co^II^ **L^A^**_*x*_ **L^B^**_4−*x*_ cages was formed in a statistical ratio in the absence of guests. Upon incorporation of anions able to interact preferentially with some library members, the products obtained were redistributed in favor of the best anion binders. To quantify the magnitudes of these templation effects, ESI‐MS was used to gauge the effect of each template upon library redistribution.

## Graphical abstract

**A statistical library of dynamic tetrahedral cages** was formed by self‐assembly of building blocks of similar sizes. ESI‐MS was used to probe the subtle effects of anion binding on the dynamic library formed. Upon the addition of anions able to bind within the library members, an increase or decrease of the proportion of each congener of the library was observed, allowing for information on relative binding affinities to be gained via MS.

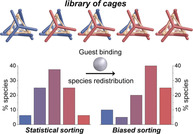

Molecular recognition is a fundamental process within biological systems. The conformational restructuring which a system undergoes upon the introduction of additional components^1^ is a crucial aspect of processes such as drug–protein interactions.^2^ Synthetic supramolecular systems can be engineered to mimic their biological counterparts,^3^ where the reconfiguration of species is enabled by the dynamic nature of the interactions between building blocks.^4^ Coordination cages are capable of reorganization upon stimuli such as light,^5^ pH changes,^6^ or guest templation^7^ and thus represent attractive reorganizing systems to study.

The outcome of self‐sorting^8^ in dynamic libraries can be influenced by the addition of new components to libraries,^9^ which interact preferentially with certain library members.^10^ Mass spectrometry has proven to be a useful technique to quantify templation effects on these systems,^11^ which could allow for better understanding of the binding processes that induce molecular reconfiguration.

The Fe^II^_4_L_4_ tetrahedral cages prepared from trianilines **A** or **B** were reported not to encapsulate guest molecules, due to their small cavity sizes.^12^ We anticipated that preparing analogous cages with Co^II^ instead of Fe^II^ might yield structures with slightly larger cavities, thus enabling guest uptake, as Co^II^ cages had previously been shown to encapsulate larger guests than their Fe^II^ analogs.^13^ Tetrahedral cages **1** and **2** (Figure 1) were thus synthesized by the reaction of the corresponding trianiline **A** or **B** (4 equiv), 2‐formylpyridine (12 equiv) and Co(NTf_2_)_2_ (4 equiv). The ^1^H NMR spectra of **1** and **2** were consistent with species of overall *T* symmetry (Figures S1 and S4), while ESI‐MS confirmed Co^II^_4_L_4_ stoichiometry (Figures S2 and S5). Single crystals of **1**(ClO_4_)_8_ and **2**(ClO_4_)_8_ suitable for X‐ray diffraction were obtained by slow vapor diffusion of either Et_2_O or EtOAc into solutions of **1** or **2** in CH_3_CN. The crystal structures (Figures 1, S10, S11) confirm the formation of the tetrahedral cages. The average Co^II^–Co^II^ distances for **1** and **2** were both found to be 12.0 Å, which is slightly larger than that of the Fe^II^ analogs (11.9 and 11.8 Å respectively).^12^ While cage **1** was found to crystallize without a guest bound inside its cavity, the crystal structure of **2** showed an encapsulated ClO_4_^−^ anion.

**Figure 1:**
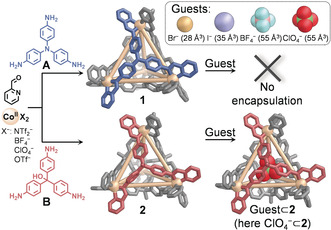
**A** and **B** self‐assembled with 2‐formylpyridine and Co^II^ to form **1** and **2**, respectively. Three‐dimensional views are constructed from the X‐ray crystal structures of **1** and ClO_4_^−^⊂**2** (Co^II^: orange, **L^A^**: blue, **L^B^**: red; orange lines indicate the closest metal–metal separations, to illustrate geometry). The insert gives the list of guests encapsulated by **2** but not **1** along with their volumes (Å^3^).

Br^−^, I^−^, BF_4_^−^, and ClO_4_^−^ anions, as well as acetonitrile, were found to bind within **2** (see Supporting Information section 4.2). Internal guest binding was indicated by the appearance of a new set of ^1^H NMR signals upon addition of these anions, consistent with the formation of a host–guest complex in slow exchange on the NMR time scale.^14^ The relative binding affinities depended on guest size, with smaller species binding more strongly within **2**, in the order Br^−^ > I^−^ ≫ BF_4_^−^ ≥ ClO_4_^−^ ≫ CH_3_CN.^15^ As the amount of anion X^−^ added increased, the concentration of host‐guest complex (X^−^⊂**2**) reached a plateau at a concentration below 1:1 binding (Figures S31, S32). The analysis described in section 5 in the Supporting Information allowed us to conclude that anion encapsulation was competing with anion‐TBA^+^ association (Figures S29, S30) and with the encapsulation of CH_3_CN, in geared equilibrium processes. These three association phenomena were considered together (Supporting Information section 5.3), producing the following association constants in CD_3_CN: *K*_Br_‐=16000±1000 m ^−1^, *K*_I_‐=4800±400 m ^−1^, *K* ${}_{\mathrm{BF}{}_{4}}$ ‐=580±50 m ^−1^, *K* ${}_{\mathrm{ClO}{}_{4}}$ ‐=1100±100 m ^−1^, *K* ${}_{\mathrm{CH}{}_{3}\mathrm{CN}}$ =0.0072±0.0004 m ^−1^.

No binding was observed within **1** when the anions studied were added following cage formation (Figure S18) or when cage **1** was formed in the presence of the anions (Figures S19 and S20). Given that **2** encapsulates a variety of anions, we were surprised that host **1** did not encapsulate any of these guests. Calculations of the cages’ internal voids (Figure S15) indicated that the cavities of **1** and **2** were of nearly equal volumes (59 Å^3^ and 56 Å^3^, respectively). The cavity in **1** was expected to accommodate Br^−^ and I^−^, with occupancies of 48 % and 59 %, respectively (Table S2).^16^ Although BF_4_^−^⊂**1** and ClO_4_^−^⊂**1** are predicted to have ≈95 % occupancy, the X‐ray structure of ClO_4_^−^⊂**2** demonstrated that such high occupancy is possible. Guest binding may be hindered by the lesser degree of pyramidalization in nitrogen‐centered **L^A^** than in hydroxymethyl‐centered **L^B^** (Supporting Information Section 3.4). In addition, we hypothesize that the *p* orbitals on four facial nitrogen atoms contribute electron density to the microenvironment within the cavity, thus destabilizing the binding of anionic guests. This hypothesis is supported by molecular orbital and electrostatic potential calculations (Supporting Information section 3.6).

Given the similarities in size and geometry between tritopic amines **A** and **B**, and the product complexes **1** and **2**, we investigated the formation of heteroleptic libraries of Co^II^ **L^A^**_*x*_ **L^B^**_4−*x*_ cages in the presence of guest molecules. The reaction of **A** and **B** (1 equiv each), 2‐formylpyridine (6 equiv) and Co(NTf_2_)_2_ (2 equiv) in CH_3_CN led to the formation of a library of cages (**Lib** ${}_{NTf{}_{2}}$ ) as confirmed by ESI‐MS and ^1^H NMR (Figures 2 a and b, S33 and S34). Slow diffusion of Et_2_O into the solution of **Lib** ${}_{NTf{}_{2}}$ in the presence of ClO_4_^−^ produced crystals suitable for diffraction studies (Supporting Information section 3.3). Single‐crystal X‐ray diffraction afforded a structure displaying disorder that was best modeled whereby each face of each cage had a 50 % probability of incorporating a residue of **A**, and 50 % **B**.

**Figure 2:**
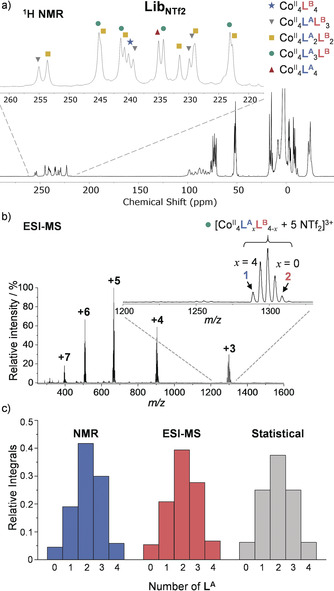
a) ^1^H NMR spectrum of **Lib** ${}_{NTf{}_{2}}$ with each of the imine signals attributed to one of the cage species in the insert. b) Low‐resolution ESI‐MS of **Lib** ${}_{NTf{}_{2}}$ . The +3 region of the mass spectra is expanded, showing the peak clusters corresponding to cages in the absence of a template. c) Normalized integrals of **Lib** ${}_{NTf{}_{2}}$ obtained by NMR and ESI‐MS compared to the statistical binomial distribution.

When the reaction was carried with sub‐stoichiometric amounts of 2‐formylpyridine (3 equiv instead of 6 equiv), a different library **Lib′** ${}_{NTf{}_{2}}$ was formed, where structures of composition Co^II^_4_ **L^A^**_*x*_ **L^B^**_4−*x*_ with 2≤*x*≤4 were expressed predominantly (Figure S37). Three sets of imine signals, containing six, four, and one resonance, respectively, were observed in the ^1^H NMR spectrum of **Lib′** ${}_{NTf{}_{2}}$ (Figure S38). Based upon symmetry, these sets of signals were assigned to Co^II^_4_ **L^A^**_2_ **L^B^**_2_, Co^II^_4_ **L^A^**_3_ **L^B^** and Co^II^_4_ **L^A^**_4_, respectively. Each of the 16 signals observed in the imine region of the ^1^H NMR spectrum of **Lib** ${}_{NTf{}_{2}}$ were thus attributed to one of the Co^II^ **L^A^**_*x*_ **L^B^**_4−*x*_ cage congeners (Figures 2 a and S39). Monitoring the formation of **Lib** ${}_{NTf{}_{2}}$ over time, suggested that the final equilibrated library developed from a more complex mixture of species present upon mixing. Heating at 70 °C for 18 h was required to approach equilibrium (Supporting Information section 6.3).

The distribution of species within **Lib** ${}_{NTf{}_{2}}$ was examined with ^1^H NMR and ESI‐MS (Supporting Information section 7.2). Since similar ESI response factors were observed for **1** and **2** (Figure S48), we hypothesized that the heteroleptic Co^II^ **L^A^**_*x*_ **L^B^**_4−*x*_ cages might have similar response factors as well. The integral of the signal for each species might thus reflect its concentration, relative to the others present in solution. No significant difference between the ratio of species in **Lib** ${}_{NTf{}_{2}}$ was observed with either analytical method (NMR or ESI‐MS), validating the use of ESI‐MS spectra to determine the relative concentrations of cage species in solution. In addition, the collection of Co^II^_4_ **L^A^**_*x*_ **L^B^**_(4−*x*)_ species was observed in an approximately 1:4:6:4:1 ratio both by NMR and ESI‐MS, close to the expected binomial distribution (Figure 2 c). The ratio of species was also found to be time‐independent in the ESI‐MS traces (Supporting Information Section 8).

Four libraries of mixed cages (**Lib_X_**, where X=Br^−^, I^−^, BF_4_^−^ or ClO_4_^−^) were prepared by the addition of the tetrabutylammonium salt of the corresponding anion X^−^ (2 equiv) to **Lib** ${}_{NTf{}_{2}}$ (Figure 5). The overlap between some signals and the low intensity of the ^1^H NMR signals corresponding to the host–guest complexes limited our ability to deconvolute them (Figure S42 and S45), therefore precluding quantification of the species present in solution. Thus, detailed ESI‐MS studies were undertaken.

The ESI‐MS spectra of the libraries in the presence of template anions (**Lib_X_**) displayed clusters of peaks corresponding to individual Co^II^_4_ **L^A^**_*x*_ **L^B^**_(4−*x*)_ cages associated with zero, one or more X^−^ (Figures 3 and S43, S44, S46, S47), indicative of X^−^ associating either externally or internally with the cages. The influence of encapsulating anion X^−^ on the library constitution could be deciphered by analyzing the distribution of the library members X^−^⊂Co^II^_4_ **L^A^**_*x*_ **L^B^**_4−*x*_, which was obtained by subtracting the distributions of library members having externally associated X^−^ (Supporting Information Section 7.3). The distributions thus observed deviated strongly from the near‐binomial distribution observed for **Lib** ${}_{NTf{}_{2}}$ , with anion templation favoring the incorporation of **L^B^** into the structures. The greatest deviations were observed for the smaller anions (Figure 4 a), corresponding to the trends observed for anion binding affinity within **2**: Br^−^ > I^−^ ≫ BF_4_^−^ > ClO_4_^−^.

**Figure 3:**
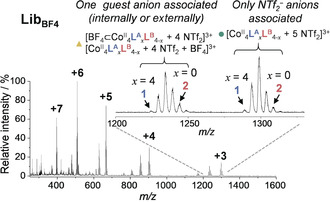
Low‐resolution ESI‐MS of **Lib** ${}_{BF{}_{4}}$ obtained after addition of TBABF_4_ (2 equiv) to **Lib** ${}_{NTf{}_{2}}$ . The +3 region of the mass spectra is expanded, showing the peak clusters corresponding to cages with no BF_4_^−^ (green circle) and cages with one BF_4_^−^ associated (yellow triangle).

**Figure 4:**
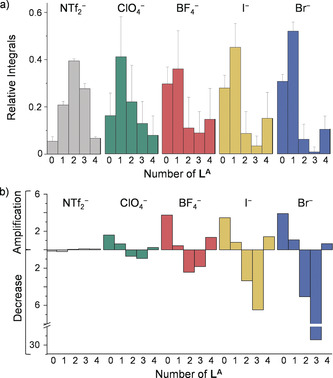
a) Normalized ESI‐MS integrals of libraries with no guest (NTf_2_^−^, gray) and after addition of guests (Br^−^, I^−^, BF_4_^−^ and ClO_4_^−^ in blue, yellow, red, and green, respectively). Each experiment was repeated three times and averaged across all charge states to obtain standard deviations, of which only positive values are shown as error bars for clarity. b) Factor of amplification or decrease of each congener generated in the libraries relative to the binomial distribution baseline with no amplification.

Cage **2** was not observed to be the major species present in solution, with Co^II^ **L^A^L^B^**_3_ species being favoured statistically (Figure 4 a). We calculated the deviation of the proportions of the generated cages from the expected binomial distribution for each of the five libraries (Figure 4 b and Supporting Information Section 7.3). While no significant changes were observed for **Lib** ${}_{NTf{}_{2}}$ , greater perturbations were observed for the libraries in the presence of templating anions. The amplitude of changes was observed to correlate with anion binding affinities. Cage **2** was amplified the most, with amplification factors of 160 % for **Lib** ${}_{ClO{}_{4}}$ , 370 % for **Lib** ${}_{BF{}_{4}}$ , 350 % for **Lib_I_** and 390 % for **Lib_Br_** compared to **Lib** ${}_{NTf{}_{2}}$ . Co^II^ **L^A^**_2_ **L^B^**_2_ and Co^II^ **L^A^**_3_ **L^B^** were clearly disfavoured, with decreases of up to 29‐fold observed in the case of **Lib_Br_**. However, cage **1**, which incorporates only **A** residues, also exhibited amplification as a result of the anion templation of other cages in solution. Cage **1** was not observed to encapsulate any of the anions present in the libraries tested (Figures S18–S20). Thus, we suggest the high proportion of **1** to be due to it serving as a ‘sink’ for residual **A** that were not incorporated into the anion binding cages (Figure 5).^17^

**Figure 5:**
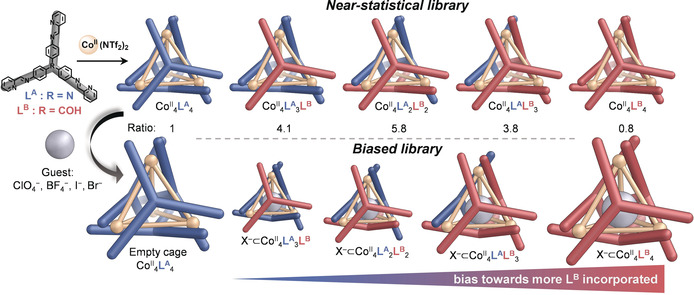
Formation of a library of Co^II^ **L^A^**_*x*_ **L^B^**_(4−*x*)_, 0≤*x*≤4. The library expressed a near‐statistical distribution of cage species in the absence of a guest. A bias towards structures incorporating more **L^B^** was observed when guests were added.

The magnitudes of the deviations of the libraries of Co^II^_4_ **L^A^**_*x*_ **L^B^**_4−*x*_ complexes (Figure 4 a) from the statistically‐expected values were used to calculate relative Gibbs free energies for each library member (Supporting Information section 7).^18^ This analysis was based on the observation that mass spectrometric response factors were similar for cages **1** and **2**, and that as a consequence, the values of the integrals reflected the quantity of each species in solution. These energies were plotted as a function of the number of **L^A^** incorporated for each anion used (Figure 6). Cage **2** was the most stable structure in all libraries, as reflected in the greater degree of amplification of this species. This observation suggests the differences in relative energies of a few kJ mol^−1^ not to be enough to lead to the exclusive formation of cage **2**. The energetic cost was raised for each **L^A^** incorporated due to the poorer fit for anions within cavities surrounded by more **A** residues, as discussed above. The library member Co^II^ **L^A^**_3_ **L^B^**, which should be affected by the presence of a template to a greater extent, had the least favorable relative energy in most cases. Anions which bound most strongly within **2** perturbed the libraries from a binomial distribution to a greater degree.

**Figure 6:**
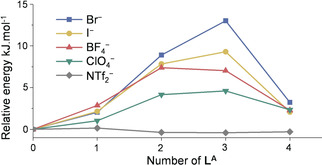
Relative energies (kJ mol^−1^) of each Co^II^_4_ **L^A^**_*x*_ **L^B^**_4−*x*_ cage within the libraries. Each experiment was repeated three times and averaged across all charge states.

A fine‐grained study of guest binding within complex heteroleptic supramolecular libraries was carried out. Cage **2**, the strongest anion binder, was amplified to a greater degree than any other species, while Co^II^ **L^A^**_3_ **L^B^** was the least favored structure. Large anions that fit most poorly within the cage cavities resulted in lower stabilization of library members, and thus less species redistribution. ESI‐MS hence allowed quantification of the degree to which guest binding favored structures that incorporated more of the ligand that is best able to accommodate the guest. Related ESI‐MS methods, used alongside other quantitative techniques such as NMR, may thus enable quantification of molecular interactions and reconfigurations in increasingly complex systems.

## Conflict of interest

The authors declare no conflict of interest.

## Supporting information

As a service to our authors and readers, this journal provides supporting information supplied by the authors. Such materials are peer reviewed and may be re‐organized for online delivery, but are not copy‐edited or typeset. Technical support issues arising from supporting information (other than missing files) should be addressed to the authors.

Supplementary

## References

[1] M. Hochgürtel , H. Kroth , D. Piecha , M. W. Hofmann , C. Nicolau , S. Krause , O. Schaaf , G. Sonnenmoser , A. V. Eliseev , Proc. Natl. Acad. Sci. USA 2002, 99, 3382–3387. 11891312 10.1073/pnas.052703799 PMC122532

[2] D. E. Scott , A. R. Bayly , C. Abell , J. Skidmore , Nat. Rev. Drug Discovery 2016, 15, 533. 27050677 10.1038/nrd.2016.29

[3a] M. J. Wiester , P. A. Ulmann , C. A. Mirkin , Angew. Chem. Int. Ed. 2011, 50, 114; 10.1002/anie.201000380 20922725

[3b] M. Mondal , A. K. H. Hirsch , Chem. Soc. Rev. 2015, 44, 2455–2488; 25706945 10.1039/c4cs00493k

[3c] D. Zamora-Olivares , T. S. Kaoud , K. N. Dalby , E. V. Anslyn , J. Am. Chem. Soc. 2013, 135, 14814–14820; 23991633 10.1021/ja407397z PMC3846390

[3d] G. Yang , W. Zheng , G. Tao , L. Wu , Q.-F. Zhou , Z. Kochovski , T. Ji , H. Chen , X. Li , Y. Lu , H.-m. Ding , H.-B. Yang , G. Chen , M. Jiang , ACS Nano 2019, 13, 13474–13485; 31651143 10.1021/acsnano.9b07134

[3e] S. Ulrich , P. Dumy , Chem. Commun. 2014, 50, 5810–5825; 10.1039/c4cc00263f 24675774

[3f] P. M. Punt , G. H. Clever , Chem. Sci. 2019, 10, 2513–2518. 30931097 10.1039/c8sc05020a PMC6399679

[4a] S. J. Rowan , S. J. Cantrill , G. R. L. Cousins , J. K. M. Sanders , J. F. Stoddart , Angew. Chem. Int. Ed. 2002, 41, 898–952; 10.1002/1521-3773(20020315)41:6<898::aid-anie898>3.0.co;2-e 12491278

[4b] D. Komáromy , M. C. A. Stuart , G. Monreal Santiago , M. Tezcan , V. V. Krasnikov , S. Otto , J. Am. Chem. Soc. 2017, 139, 6234–6241; 28398730 10.1021/jacs.7b01814 PMC5423079

[4c] J.-M. Lehn , Chem. Soc. Rev. 2007, 36, 151–160; 17264919 10.1039/b616752g

[4d] L. Wang , L. Cheng , G. Li , K. Liu , Z. Zhang , P. Li , S. Dong , W. Yu , F. Huang , X. Yan , J. Am. Chem. Soc. 2020, 142, 2051–2058. 31905287 10.1021/jacs.9b12164

[5a] R.-J. Li , J. J. Holstein , W. G. Hiller , J. Andréasson , G. H. Clever , J. Am. Chem. Soc. 2019, 141, 2097–2103; 30620873 10.1021/jacs.8b11872

[5b] S. Oldknow , D. R. Martir , V. E. Pritchard , M. A. Blitz , C. W. G. Fishwick , E. Zysman-Colman , M. J. Hardie , Chem. Sci. 2018, 9, 8150–8159; 30542566 10.1039/c8sc03499k PMC6238882

[5c] M. J. Burke , G. S. Nichol , P. J. Lusby , J. Am. Chem. Soc. 2016, 138, 9308–9315; 27351912 10.1021/jacs.6b05364

[5d] D. Samanta , P. S. Mukherjee , J. Am. Chem. Soc. 2014, 136, 17006–17009. 25423470 10.1021/ja511360e

[6a] K. Kurihara , K. Yazaki , M. Akita , M. Yoshizawa , Angew. Chem. Int. Ed. 2017, 56, 11360–11364; 10.1002/anie.201703357 28649701

[6b] Y. Liu , B. Shi , H. Wang , L. Shangguan , Z. Li , M. Zhang , F. Huang , Macromol. Rapid Commun. 2018, 39, 1800655; 10.1002/marc.201800655 30318827

[6c] S. M. Jansze , G. Cecot , K. Severin , Chem. Sci. 2018, 9, 4253–4257. 29780555 10.1039/c8sc01108g PMC5944229

[7a] R. L. Paul , Z. R. Bell , J. C. Jeffery , J. A. McCleverty , M. D. Ward , Proc. Natl. Acad. Sci. USA 2002, 99, 4883–4888; 11929962 10.1073/pnas.052575199 PMC122688

[7b] R. Custelcean , Chem. Soc. Rev. 2014, 43, 1813–1824; 24384869 10.1039/c3cs60371g

[7c] H. T. Chifotides , K. R. Dunbar , Acc. Chem. Res. 2013, 46, 894–906; 23477406 10.1021/ar300251k

[7d] B. Wang , Z. Zang , H. Wang , W. Dou , X. Tang , W. Liu , Y. Shao , J. Ma , Y. Li , J. Zhou , Angew. Chem. Int. Ed. 2013, 52, 3756–3759; 10.1002/anie.201210172 23427114

[7e] S. Mirtschin , A. Slabon-Turski , R. Scopelliti , A. H. Velders , K. Severin , J. Am. Chem. Soc. 2010, 132, 14004–14005; 20860361 10.1021/ja1063789

[7f] K. Kumazawa , Y. Yamanoi , M. Yoshizawa , T. Kusukawa , M. Fujita , Angew. Chem. Int. Ed. 2004, 43, 5936–5940; 10.1002/anie.200460868 15547899

[7g] R. Sekiya , M. Fukuda , R. Kuroda , J. Am. Chem. Soc. 2012, 134, 10987–10997; 22663382 10.1021/ja303634u

[7h] C. M. Hong , D. M. Kaphan , R. G. Bergman , K. N. Raymond , F. D. Toste , J. Am. Chem. Soc. 2017, 139, 8013–8021. 28581740 10.1021/jacs.7b03812

[8a] Z. He , W. Jiang , C. A. Schalley , Chem. Soc. Rev. 2015, 44, 779–789; 25374006 10.1039/c4cs00305e

[8b] M. M. Safont-Sempere , G. Fernández , F. Würthner , Chem. Rev. 2011, 111, 5784–5814; 21846150 10.1021/cr100357h

[8c] Y.-R. Zheng , H.-B. Yang , B. H. Northrop , K. Ghosh , P. J. Stang , Inorg. Chem. 2008, 47, 4706–4711; 18433099 10.1021/ic800038j

[8d] M. Kołodziejski , A. R. Stefankiewicz , J.-M. Lehn , Chem. Sci. 2019, 10, 1836–1843. 30842852 10.1039/c8sc04598d PMC6369437

[9a] D. Beaudoin , F. Rominger , M. Mastalerz , Angew. Chem. Int. Ed. 2017, 56, 1244–1248; 10.1002/anie.201610782 28004471

[9b] S. P. Black , D. M. Wood , F. B. Schwarz , T. K. Ronson , J. J. Holstein , A. R. Stefankiewicz , C. A. Schalley , J. K. M. Sanders , J. R. Nitschke , Chem. Sci. 2016, 7, 2614–2620; 28660033 10.1039/c5sc04906g PMC5477050

[9c] C.-W. Hsu , O. Š. Miljanić , Chem. Commun. 2016, 52, 12357–12359; 10.1039/c6cc06772g 27711334

[9d] A. R. Stefankiewicz , J. K. M. Sanders , Chem. Commun. 2013, 49, 5820–5822; 10.1039/c3cc41158c 23525236

[9e] W. Makiguchi , J. Tanabe , H. Yamada , H. Iida , D. Taura , N. Ousaka , E. Yashima , Nat. Commun. 2015, 6, 7236; 26051291 10.1038/ncomms8236 PMC4468858

[9f] E. Badetti , N. A. Carmo dos Santos , F. A. Scaramuzzo , C. Bravin , K. Wurst , G. Licini , C. Zonta , RSC Adv. 2018, 8, 19494–19498; 10.1039/c8ra03989e PMC9080712 35540993

[9g] C. A. Wiley , L. R. Holloway , T. F. Miller , Y. Lyon , R. R. Julian , R. J. Hooley , Inorg. Chem. 2016, 55, 9805–9815; 27623080 10.1021/acs.inorgchem.6b01644

[9h] G. Men , J.-M. Lehn , Chem. Sci. 2019, 10, 90–98. 30713621 10.1039/c8sc03858a PMC6333171

[10a] O. Š. Miljanić , Chem 2017, 2, 502–524;

[10b] J. Atcher , A. Moure , J. Bujons , I. Alfonso , Chem. Eur. J. 2015, 21, 6869–6878; 25777367 10.1002/chem.201406155

[10c] A. M. Valdivielso , F. Puig-Castellví , J. Atcher , J. Solà , R. Tauler , I. Alfonso , Chem. Eur. J. 2017, 23, 10789–10799; 28480991 10.1002/chem.201701294

[10d] A. Blanco-Gómez , T. Rama , O. Domarco , I. Neira , V. Blanco , J. M. Quintela , M. D. García , C. Peinador , Dalton Trans. 2017, 46, 15671–15675; 29106423 10.1039/c7dt03379f

[10e] J. Septavaux , G. Germain , J. Leclaire , Acc. Chem. Res. 2017, 50, 1692–1701; 28644617 10.1021/acs.accounts.7b00147

[10f] M. Ziegler , J. J. Miranda , U. N. Andersen , D. W. Johnson , J. A. Leary , K. N. Raymond , Angew. Chem. Int. Ed. 2001, 40, 733–736; 11241606

[10g] K. Severin , Chem. Eur. J. 2004, 10, 2565–2580; 15146528 10.1002/chem.200305660

[10h] N. Halina , P. Jaroslaw , G. Rafal , M. Robert , T. Dominik , P. Barbara , B. Andrzej , P. Anna , M. Jean-Francois , G. Johann , B. Marc Le , Comb. Chem. High Throughput Screening 2006, 9, 753–770.

[11a] C. Bravin , E. Badetti , R. Puttreddy , F. Pan , K. Rissanen , G. Licini , C. Zonta , Chem. Eur. J. 2018, 24, 2936–2943; 29205565 10.1002/chem.201704725

[11b] U. Warzok , M. Marianski , W. Hoffmann , L. Turunen , K. Rissanen , K. Pagel , C. A. Schalley , Chem. Sci. 2018, 9, 8343–8351. 30542581 10.1039/c8sc03040e PMC6243472

[12] R. A. Bilbeisi , J. K. Clegg , N. Elgrishi , X. de Hatten , M. Devillard , B. Breiner , P. Mal , J. R. Nitschke , J. Am. Chem. Soc. 2012, 134, 5110–5119. 22043943 10.1021/ja2092272

[13] T. K. Ronson , C. Giri , N. Kodiah Beyeh , A. Minkkinen , F. Topić , J. J. Holstein , K. Rissanen , J. R. Nitschke , Chem. Eur. J. 2013, 19, 3374–3382. 23344965 10.1002/chem.201203751

[14a] F. J. Rizzuto , J. P. Carpenter , J. R. Nitschke , J. Am. Chem. Soc. 2019, 141, 9087–9095; 31079455 10.1021/jacs.9b03776

[14b] F. J. Rizzuto , W.-Y. Wu , T. K. Ronson , J. R. Nitschke , Angew. Chem. Int. Ed. 2016, 55, 7958–7962; 10.1002/anie.201602135 PMC4999047 27095669

[15] J. K. Clegg , J. Cremers , A. J. Hogben , B. Breiner , M. M. J. Smulders , J. D. Thoburn , J. R. Nitschke , Chem. Sci. 2013, 4, 68–76.

[16] S. Mecozzi , J. Rebek , Chem. Eur. J. 1998, 4, 1016–1022.

[17] A. Osypenko , S. Dhers , J.-M. Lehn , J. Am. Chem. Soc. 2019, 141, 12724–12737. 31364844 10.1021/jacs.9b05438

[18] M. Kieffer , B. S. Pilgrim , T. K. Ronson , D. A. Roberts , M. Aleksanyan , J. R. Nitschke , J. Am. Chem. Soc. 2016, 138, 6813–6821. 27145216 10.1021/jacs.6b02445

