# Supplementary material for: Guest Binding Drives Host Redistribution in Libraries of CoII 4L4 Cages
Source: Angew Chem Int Ed Engl. 2020 May 7;59(28):11369–73. doi: 10.1002/anie.202004627 (PMC7383889; doi:10.1002/anie.202004627)
Supplement: Supplementary file 1 — Supplementary [file ANIE-59-11369-s001.pdf]

Angewandte Chemie
Eine Zeitschrift der Gesellschaft Deutscher Chemiker

Supporting Information

Guest Binding Drives Host Redistribution in Libraries of $Co^{II}{}_4L_4$ Cages

*Marion Kieffer, Rana A. Bilbeisi, John D. Thoburn, Jack K. Clegg, and Jonathan R. Nitschke**

anie_202004627_sm_miscellaneous_information.pdf

Table of Contents

Table of Contents 1
1) Materials and methods 2
1.1) General 2
1.2) Mass spectrometry (MS) 2
1.3) Nuclear Magnetic Resonance (NMR) 2
2) Synthesis 3
2.1) Cage 1 3
2.2) Cage 2 4
3) Crystallography 7
3.1) Crystal structure of 1 7
3.2) Crystal structure of 2 8
3.3) Crystal structure of cage library 9
3.4) Ligand pyramidalization 10
3.5) Cavity Volume Calculations 11
3.6) Calculation of Molecular Orbitals and Electrostatic Potentials 12
4) Host-guest studies 13
4.1) Guest binding in 1 13
4.2) Guest binding in 2 16
5) Binding modes in cage 2 21
5.1) Role of TBA^+ 21
5.2) Binding curves and fitting 23
5.3) Equations and mathematical model 26
Equation for [HG], [HS] and [H] 26
The Merit Function χ^2 28
Minimization Routine and Error Propagation 28
6) Characterization of libraries of heteroleptic cages 29
6.1) Lib_{NTf_2} 29
6.2) Lib'_{NTf_2} 30
6.3) Kinetic study of the formation of Lib_{NTf_2} 32
6.4) Lib_{ClO_4} and Lib_{BF_4} 33
6.5) Lib_I and Lib_{Br} 35
7) Distributions and energies calculations 37

7.1) Response factor of 1 and 2 37

7.2) Lib_{NTf_2} 37

7.3) Lib_{ClO_4}, Lib_{BF_4}, Lib_I and Lib_{Br}. 39

8) Time dependency of ESI-MS response factors 44

References 47

Author Contributions 47

1) Materials and methods

1.1) General

All reagents and solvents were purchased from commercial suppliers and were used as supplied without purification unless stated otherwise. Commercially supplied pararosaniline base **B** was found to contain impurities (including fuchsin). The material supplied by Aldrich was found to contain the least amount of contaminant and could be obtained in satisfactory purity after multiple washes with CH_3CN. Cobalt(II)bis(trifluoromethane)sulfonamide was prepared following literature procedures.[1]

1.2) Mass spectrometry (MS)

Low resolution electrospray ionisation mass spectrometry (LR-ESI-MS) was carried out on a Micromass Quattro LC mass spectrometer (cone voltage 15-25 eV, desolvation temp. 313 K, ionization temp. 313 K) infused from a Harvard Syringe Pump at a rate of 10 μL min^{-1}. High resolution electrospray ionisation mass spectrometry (HR-ESI-MS) was undertaken on a Thermofisher LTQ Orbitrap XL hybrid ion trap mass spectrometer.

1.3) Nuclear Magnetic Resonance (NMR)

NMR spectra were recorded at 298K on a Bruker 400 MHz Avance III HD Smart Probe. ^{1}H and ^{13}C chemical shift values are reported in ppm relative to the solvent residual peak. ^{19}F chemical shift values are reported in ppm relative to the internal reference peak of C_6F_6 in CD_3CN (δ = −164.90 ppm). Coupling constants (J) are reported in hertz (Hz) and the signal multiplicities are described as: s (singlet), d (doublet), t (triplet), m (multiplet) and b (broad). Wide sweep paramagnetic NMR spectra were recorded in the analogue digitisation mode with a spectral width (SW) of 407.42 ppm, a transmitter frequency offset (O1P) of 130.00 ppm and an acquisition time of 0.1 sec. Due to the experimental difficulties associated with collecting NMR data for ^{1}H nuclei with vastly different relaxation times, differences between measured and theoretical integration values were in some cases observed. While the paramagnetic nature of the complex precluded complete assignment of the proton environments, we propose that through-bond proximity of the proton environment to each Co^{II} centers dictates the extent of downfield shifting of each signal, as observed in previous reports.[1-2]

2) Synthesis

2.1) Cage 1

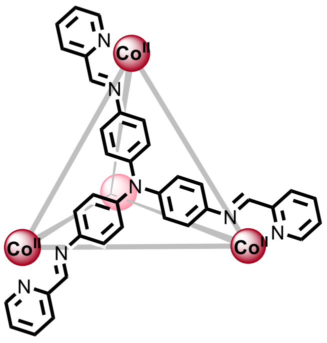

Tris(4-aminophenyl)amine **A** (9.0 mg, 31.1 µmol, 1.0 equiv.), 2-formylpyridine (10.0 mg, 93.3 µmol, 3.0 equiv.) and $Co(NTf_2)_2$ (21.5 mg, 31.1 µmol, 1.0 equiv.) were combined in CH_3CN or CD_3CN (2.5 mL) in a sealed 5 mL reaction tube. The solution was stirred and heated at 70 °C for 18 h. A dark orange stock solution of cage **1** (3.11 mM) was obtained and was used without further purification.

^{1}H NMR (400 MHz, 298K, CD_3CN) δ 234.6 (s, 12H), 87.6 (s, 12H), 71.5 (s, 12H), 51.0 (s, 12H), 14.8 (s, 12H), –7.7 (s, 24H), –25.1 (s, 24H). **^{19}F NMR** (376 MHz, 298K, CD_3CN) δ – 76.82. **LR-ESI-MS** [charge, calculated mass]: *m/z* = 1288.9 [**1**$(NTf_2)_5^{3+}$, 1288.0], 896.7 [**1**$(NTf_2)_4^{4+}$, 896.0], 661.3 [**1**$(NTf_2)_3^{5+}$, 660.8], 504.4 [**1**$(NTf_2)_2^{6+}$, 504.1], 392.4 [**1**$(NTf_2)^{7+}$, 392.1], 308.4 [**1**$^{8+}$, 308.1].

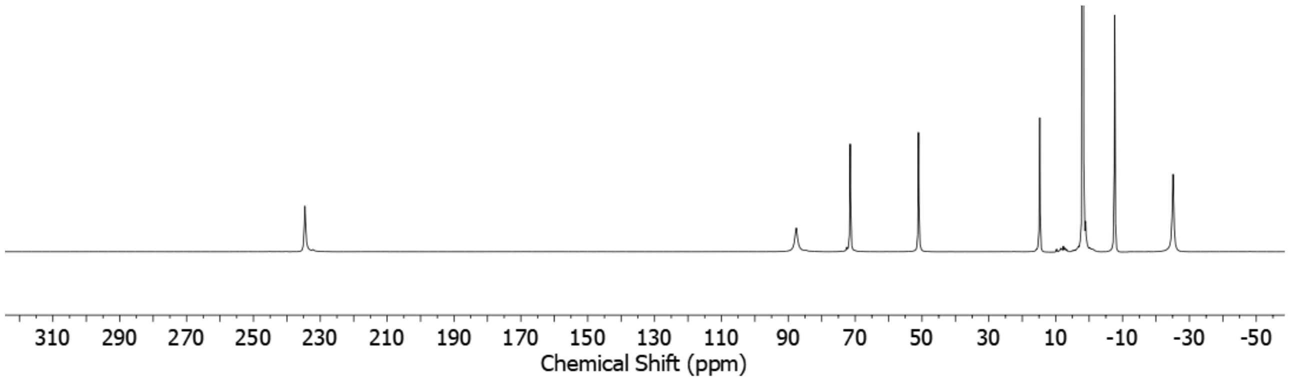

Figure S1. Wide sweep ^{1}H NMR spectrum (400 MHz, 298 K, CD_3CN) of **1**.

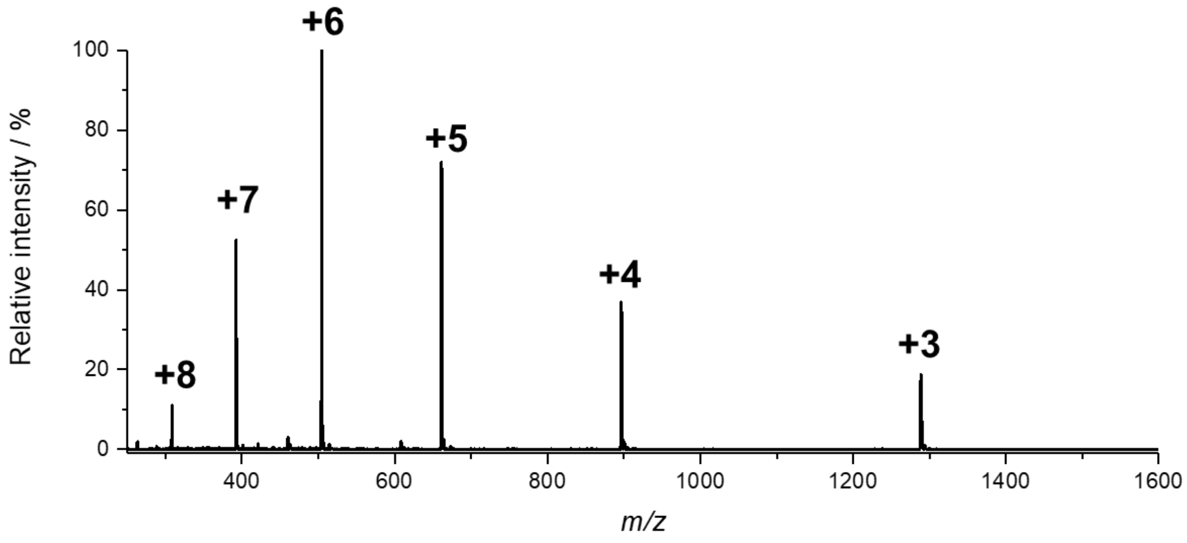

Figure S2. LR-ESI-MS mass spectrum of **1**$(NTf_2)_8$.

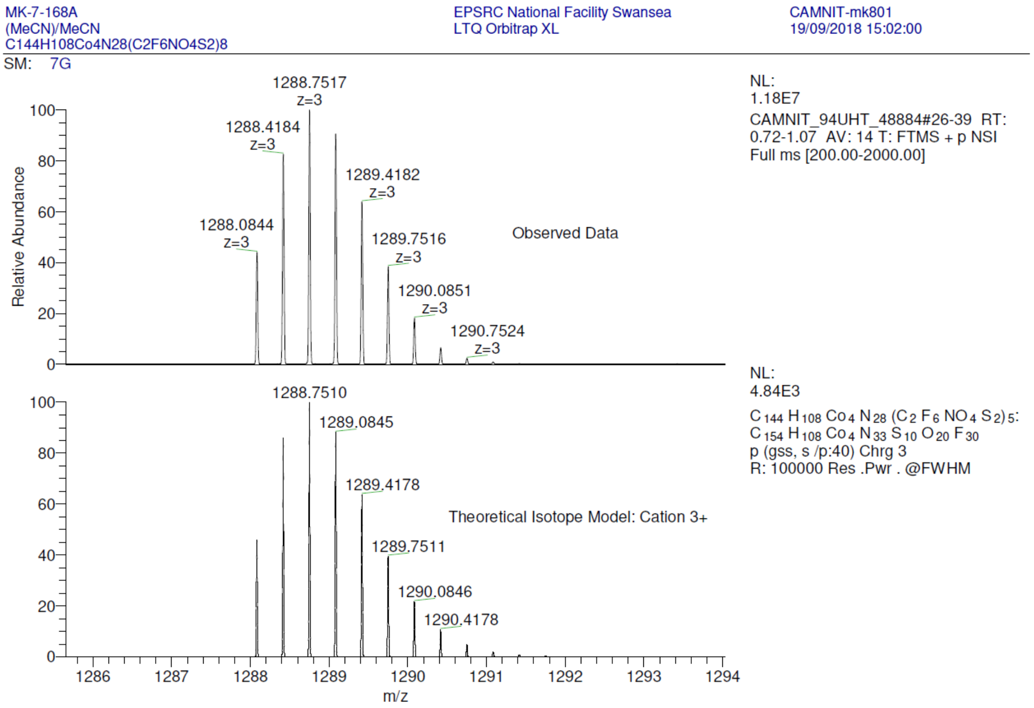

Figure S3. HR-ESI-Mass spectrum of **1**$(NTf_2)_8$ showing the observed z = +3 charge, (top) compared to the theoretical isotope pattern (bottom).

2.2) Cage 2

With $Co(NTf_2)_2$

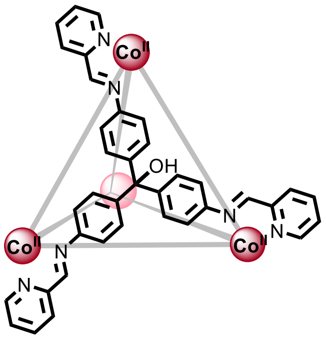

Pararosaniline **B** (9.0 mg, 31.1 µmol, 1.0 equiv.), 2-formylpyridine (10.0 mg, 93.3 µmol, 3.0 equiv.) and $Co(NTf_2)_2$ (21.5 mg, 31.1 µmol, 1.0 equiv.) were combined in CH_3CN or CD_3CN (2.5 mL) in a sealed 5 mL reaction tube. The solution was stirred and heated at 70 °C for 18 h. A dark red stock solution of cage **2** (3.11 mM) was obtained and was used without further purification.

A second minor set of signals was observed in the ^1H NMR spectrum which was attributed to the host-guest complex $CD_3CN\subset$**2** (See section 4.2 for more detail).

^{1}H NMR (400 MHz, 298K, CD_3CN) δ 240.1 (s, 12H), 236.4 (s, 12H, HG complex), 89.0 (s, 12H), 87.3 (s, 12H, HG complex), 73.6 (s, 24H), 52.0 (s, 24H), 16.2 (s, 24H), –4.666 (s, 48H), –23.8 (s, 48H). **^{19}F NMR** (376 MHz, 298K, CD_3CN) δ –79.56. **LR-ESI-MS** [charge, calculated mass]: *m/z* = 1308.9 [**2**$(NTf_2)_5^{3+}$, 1308.1], 911.7 [**2**$(NTf_2)_4^{4+}$, 911.0], 673.3 [**2**$(NTf_2)_3^{5+}$, 672.8], 514.4 [**2**$(NTf_2)_2^{6+}$, 514.1], 400.9 [**2**$(NTf_2)^{7+}$, 400.6], 315.9 [**2**$^{8+}$, 315.6].

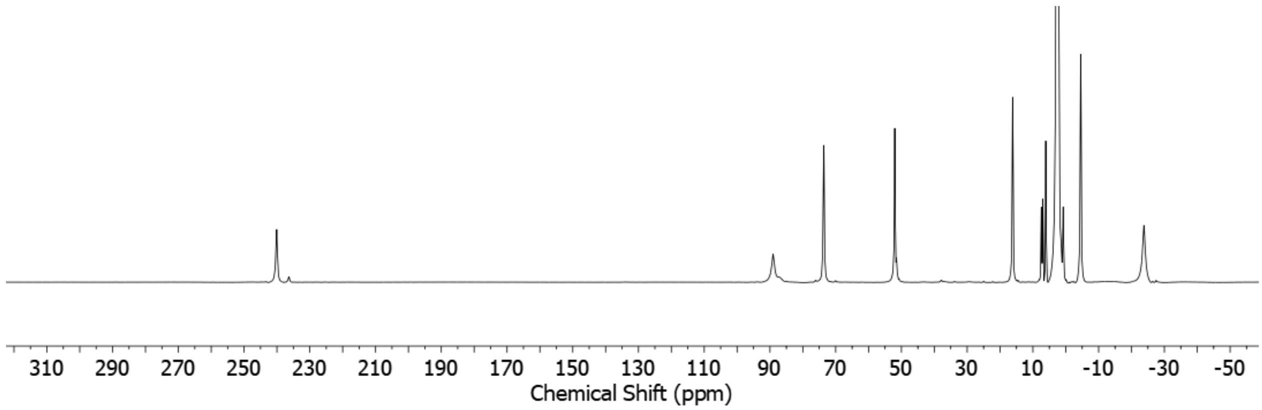

Figure S4. Wide sweep ^1H NMR spectrum (400 MHz, 298 K, CD_3CN) of **2**$(NTf_2)_8$.

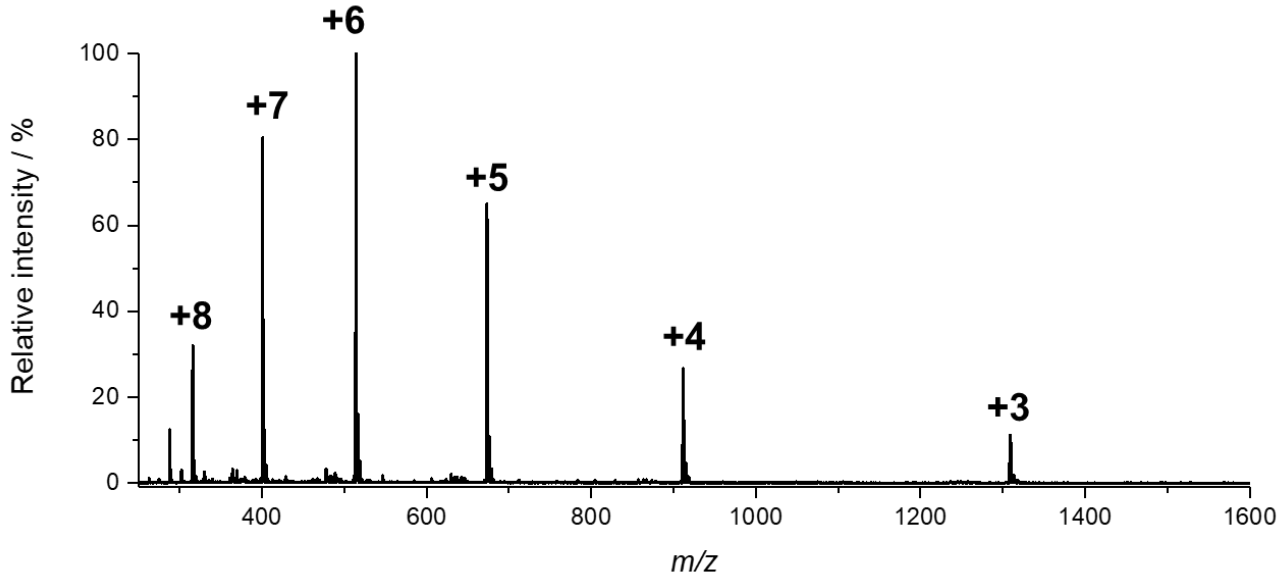

Figure S5. LR-ESI-Mass spectrum of **2**$(NTf_2)_8$.

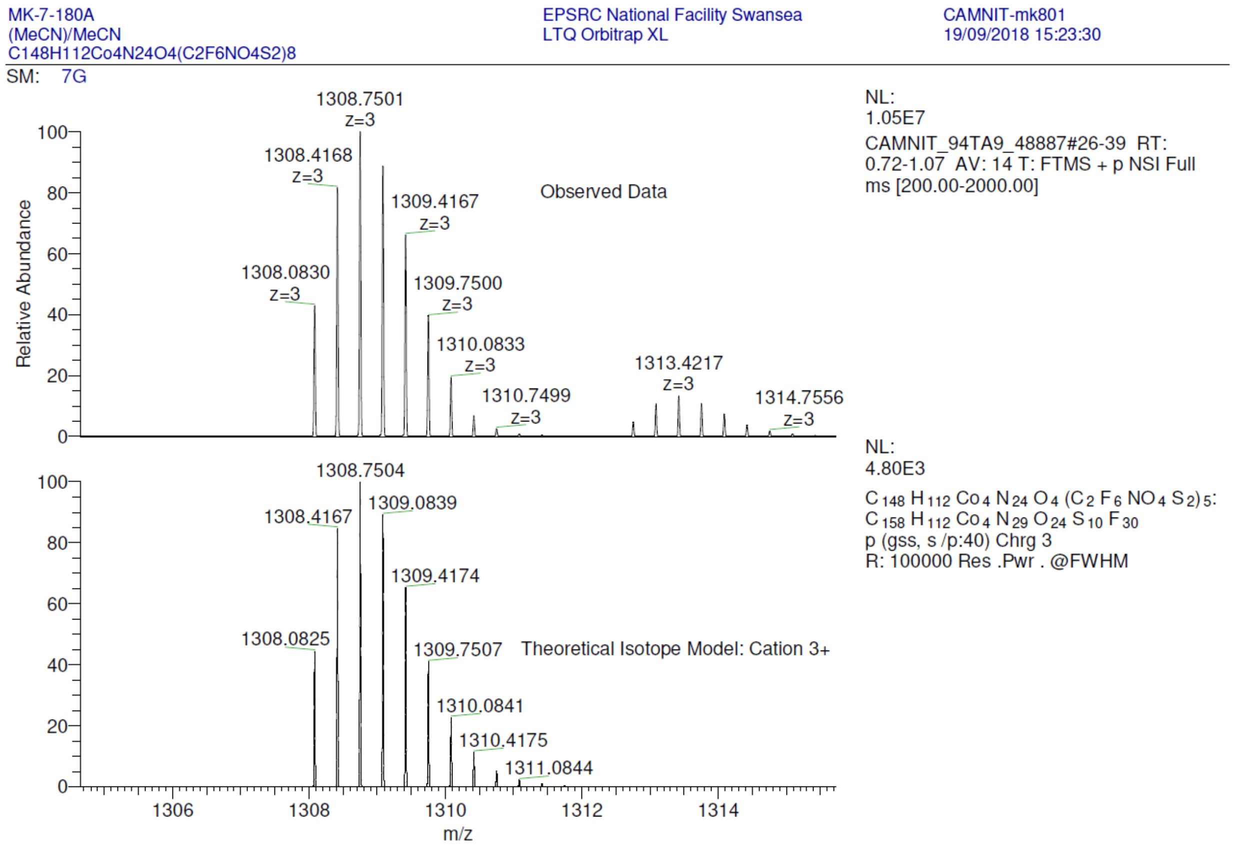

Figure S6. HR-ESI-Mass spectrum of **2**$(NTf_2)_8$ showing the observed z = +3 charge, (top) compared to the theoretical isotope pattern (bottom). The close agreement observed indicates that the **B** residues within **2** were not deprotonated.

With Co(BF$_4$)$_2$

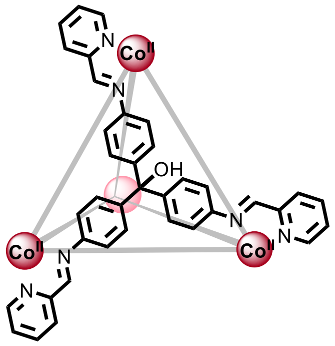

Pararosaniline **B** (9.0 mg, 31.1 µmol, 1.0 equiv.), 2-formylpyridine (10.0 mg, 93.3 µmol, 3.0 equiv.) and Co(BF$_4$)$_2$ (10.6 mg, 31.1 µmol, 1.0 equiv.) were combined in CH_3CN or CD_3CN (2.5 mL) in a sealed 5 mL reaction tube. The solution was stirred and heated at 70 °C for 18 h. A dark red stock solution of cage **2** (3.11 mM) was obtained and was used without further purification.

Three set of signals were observed in the ^{1}H NMR which were attributed to the empty cage, the host-guest complex $CD_3CN\subset$**2** and the host-guest complex $BF_4^-\subset$**2** (see section 4.2 for more detail). **^{1}H NMR** (400 MHz, 298K, CD_3CN) δ 239.7 (s, 1H, empty cage), 238.5 (s, 1H, $BF_4^-\subset$**2**), 236.1 (s, 1H, $CD_3CN\subset$**2**), 88.6 (bs, 1H, empty cage), 86.7 (bs, 1H, $CD_3CN\subset$**2**), 86.2 (bs, 1H, $BF_4^-\subset$**2**), 74.0 – 72.9 (m, 3H), 51.8 (s, 3H), 16.4 (s, 3H), –4.5 (s, 6H), –23.6 (bs, 2H, empty cage and $CD_3CN\subset$**2**), –25.7 (bs, 2H, $BF_4^-\subset$**2**). **^{19}F NMR** (376 MHz, 298K, CD_3CN) δ –143.45 (s, free BF_4^-), –200.02 (s, $BF_4^-\subset$**2**). **LR-ESI-MS** [charge, calculated mass]: *m/z* = 1523.4 [**6.10**(BF$_4$)$_6$$^{2+}$, 1523.24], 986.7 [**2**(BF$_4$)$_5$$^{3+}$, 986.5], 718.3 [**2**(BF$_4$)$_4$$^{4+}$, 718.1], 557.2 [**2**(BF$_4$)$_3$$^{5+}$, 557.1], 449.8 [**2**(BF$_4$)$_2$$^{6+}$, 449.7], 373.2 [**2**(BF$_4$)$^{7+}$, 373.1], 315.1 [**2**$^{8+}$, 315.6].

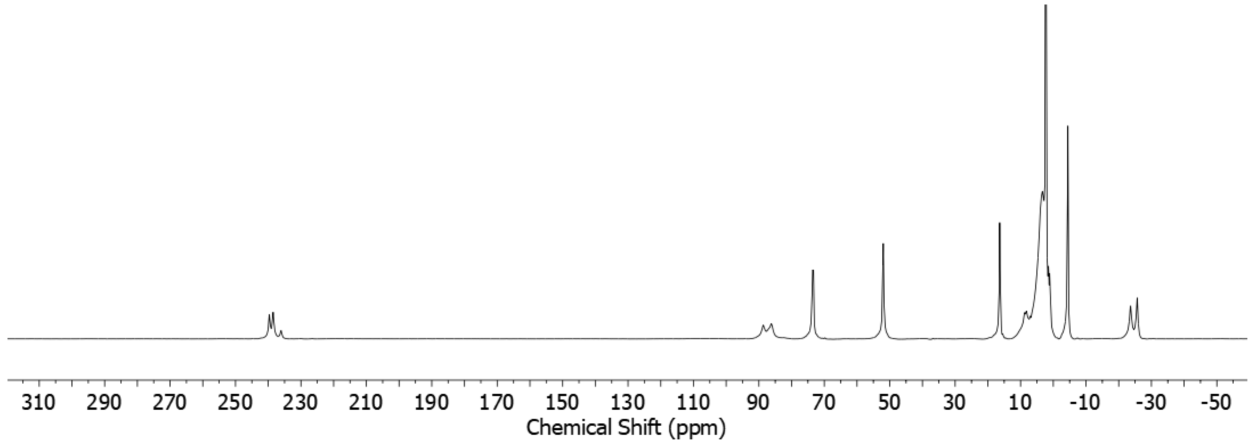

Figure S7. Wide sweep ^{1}H NMR spectrum (400 MHz, 298 K, CD_3CN) of **2**(BF$_4$)$_8$.

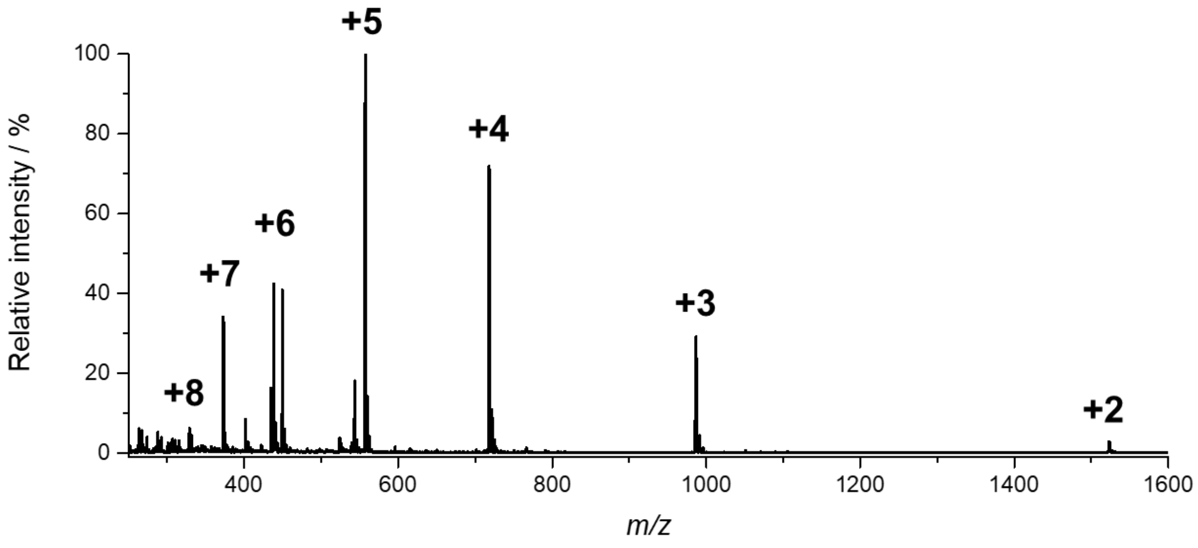

Figure S8. LR-ESI-MS mass spectrum of **2**(BF$_4$)$_8$.

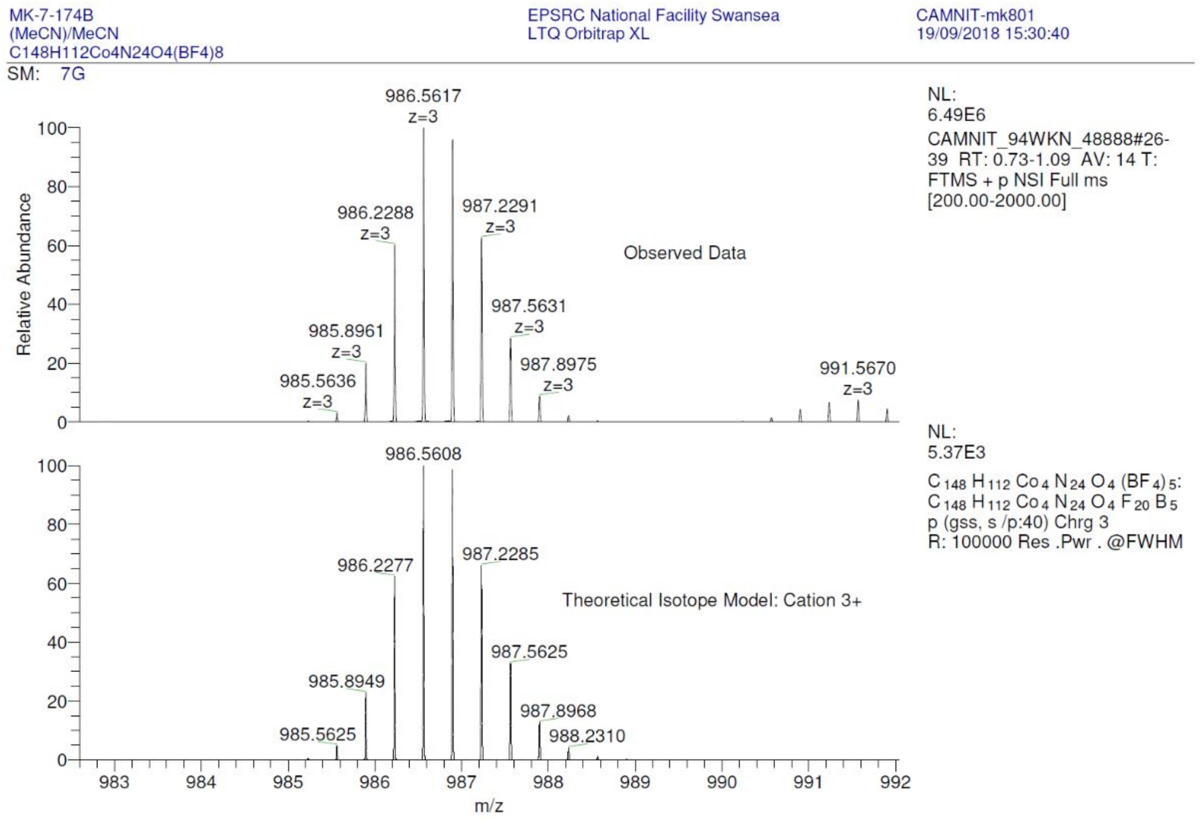

Figure S9. HR-ESI-MS mass spectrum of **2**(BF_4)$_8$ showing the observed z = +3 charge, (top) compared to the theoretical isotope pattern (bottom).

3) Crystallography

Data were collected either using a Nonius Kappa FR590 diffractometer employing graphite-monochromated Mo-K$_\alpha$ radiation generated from a sealed tube (0.71073 Å) with ω and ψ scans at 180(2) K,[3] at Beamline I19 of Diamond Light Source employing silicon double crystal monochromated synchrotron radiation (0.6889 Å) with ω scans at 100(2) K,[4] or by the UK National Crystallographic Service[5] using a Rigaku 007-HF Diffractometer employing confocal mirror monochromated Cu-K$_\alpha$ radiation generated from a rotating anode (1.5418 Å) with ω scans at 80(2) K.[4] Data integration and reduction were undertaken with HKL Denzo and Scalepack[6] or with CrystalClear.[4] Multi-scan empirical absorption corrections were applied to the data set using either SORTAV[7] or CrystalClear.[4] Subsequent computations were carried out using the WinGX-32 graphical user interface.[8] Structures were solved using SUPERFLIP[9] then refined and extended with SHELXL.[10] In general, non-hydrogen atoms with occupancies greater than 0.5 were refined anisotropically. Carbon-bound hydrogen atoms were included in idealised positions and refined using a riding model. Crystallographic data and specific details pertaining to the refinement are given below. Disorder was modelled using standard crystallographic methods including constraints, restraints and rigid bodies where necessary. Where required the SQUEEZE[11] function of PLATON[12] was employed to account for highly disordered solvent and/or anions.

3.1) Crystal structure of 1

The crystals employed in this study were both extremely small and extremely unstable. Rapid (<1 min) handling at dry ice temperatures prior to quenching in the cryostream and high intensity radiation were required to collect data. Even with these measures the diffraction was broad and weak. In addition, reflecting the poor diffraction quality of the crystal, there is a large area of smeared electron density present in the lattice. Despite many attempts to model this region of disorder as a anion molecules no reasonable fit could be found and accordingly this region was treated with the SQUEEZE[11] function of PLATON.[12]

$C_{144}H_{108}Cl_8Co_4N_{28}O_{32}$, *M* 3261.90, orthorhombic, space group I222 (#23), *a* 19.569(4), *b* 20.687(4), *c* 20.892(4) Å, V 8457(3) Å^3, D_c 1.281 g cm^{-3}, Z 2, crystal size 0.24 by 0.24 by 0.12 mm, colour Dark Brown, habit Rhombus, temperature 80(2) K, λ(CuK_α) 1.54187 Å, μ(CuK_α) 4.803 mm^{-1}, T(CRYSTALCLEAR)$_{min,max}$ 0.686, 1.000, 2θ$_{max}$ 138.20, hkl range -22 23, -25 24, -24 23, N 70261, N_{ind} 7771(R_{merge} 0.0953), N_{obs} 4940(I > 2σ(I)), N_{var} 406, residuals R1(F) 0.0984, wR2(F^2) 0.2789, GoF(all) 0.910, Δρ$_{min,max}$ -0.269, 0.274 e$^-$ Å^{-3}.

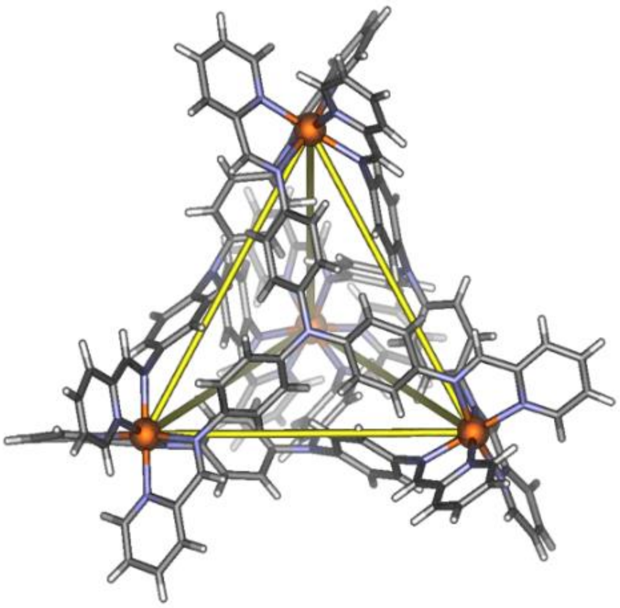

Figure S10. Schematic representation of the crystal structure of **1**(ClO_4)$_8$, anions removed for clarity.

3.2) Crystal structure of 2

The crystals employed in this study were extremely unstable to solvent loss. Rapid (<1 min) handling at dry ice temperatures prior to quenching in the cryostream was required to collect data. Even with these measures the diffraction was broad and weak. In addition, reflecting the poor diffraction quality of the crystal, the anions and solvent are significantly disordered and there is a large area of smeared electron density present in the lattice. Many of the anions and solvent that could be located were modelled with occupancies of < 1.0 (including the encapsulated perchlorate). The region of disorder that could not be reasonably modelled was treated with the SQUEEZE[11] function of PLATON.[12]

Formula $C_{166}H_{160.50}Cl_8Co_4N_{28.50}O_{42.50}$, *M* 3754.04, monoclinic, space group C2/c(#15), *a* 32.371(7), *b* 32.938(7), *c* 39.039(8) Å, *β* 110.19(3) °, *V* 39068(15) Å^3, D_c 1.276 g cm^{-3}, *Z* 8, crystal size 0.3 by 0.25 by 0.2 mm, colour orange, habit block, temperature 180(2) K, λ(MoK_α) 0.71073 Å, μ(MoK_α) 0.520 mm^{-1}, *T*(MULTI-SCAN)$_{min,max}$ 0.803, 0.982, 2θ_{max} 49.43, *hkl* range -36 38, -38 38, -45 45, *N* 146633, N_{ind} 33053(R_{merge} 0.1051), N_{obs} 12097(I > 2σ(I)), N_{var} 2111, residuals *R*1(*F*) 0.0944, *wR*2(F^2) 0.2886, GoF(all) 0.972, Δρ$_{min,max}$ -1.756, 2.163 e$^-$ Å^{-3}.

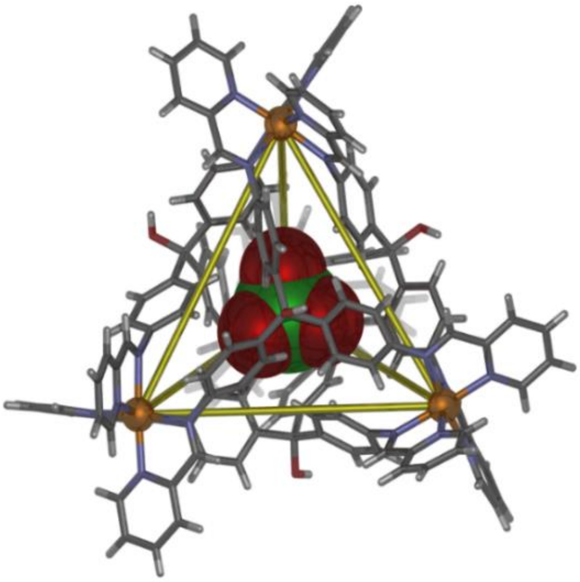

Figure S11. Schematic representation of the crystal structure of $[ClO_4\subset\mathbf{2}](ClO_4)_7$ showing the encapsulated perchlorate anion. Other anions and solvent removed for clarity.

3.3) Crystal structure of cage library

Slow diffusion of diethyl ether into an acetonitrile solution of the mixed ligand system yielded orange crystals. Despite appearing (at least visually) of good quality, the diffraction pattern obtained from these crystals with synchrotron radiation was broad. Structural analysis revealed that, although the complex crystallized in orthorhombic *Pcan* with half a tetrahedron in the asymmetric unit that two ligands A and B were present and disordered, with a 50 % occupancy each. The crystal structure is thus best described as a co-crystal composed of mixture of all possible complexes $\mathbf{L^A}_4$, $\mathbf{L^A}3$:$\mathbf{L^B}$, $\mathbf{L^A}_2$:$\mathbf{L^B}_2$, $\mathbf{L^A}$:$\mathbf{L^B}_3$ and $\mathbf{L^B}_4$. Such an observation is in good agreement with the broad diffraction pattern observed. This structure was therefore modelled using rigid bodies for each of the ligands. The ligand coordinates were taken from the crystal structures of **1** and **2**. In addition, the anions are significantly disordered and could not be suitably modelled. This region of disorder was treated with the SQUEEZE[11] function of PLATON.[12]

Formula $C_{146}H_{106}Cl_8Co_4N_{26}O_{34}$, *M* 3287.88, orthorhombic, space group Pcan (#60), *a* 19.089(7), *b* 30.403(11), *c* 36.359(11) Å, *V* 21102(12) Å^3, D_c 1.035 g cm$_{-3}$, *Z* 4, crystal size 0.05 by 0.05 by 0.05 mm, colour orange, habit block, temperature 100(2) K, λ(synchrotron) 0.6889 Å, μ(synchrotron) 0.429 mm^{-1}, T(CRYSTALCLEAR)$_{min,max}$ 0.695, 1.000, $2\theta_{max}$ 40.29, *hkl* range -19 19, -30 30, -34 36, *N* 123193, N_{ind} 10841(R_{merge} 0.1148), N_{obs} 8723(I > 2σ(I)), N_{var} 240, residuals $R1(F)$ 0.1931, $wR2(F^2)$ 0.4152, GoF(all) 1.035, $\Delta\rho_{min,max}$ -0.442, 0.490 e$^-$ Å^{-3}.

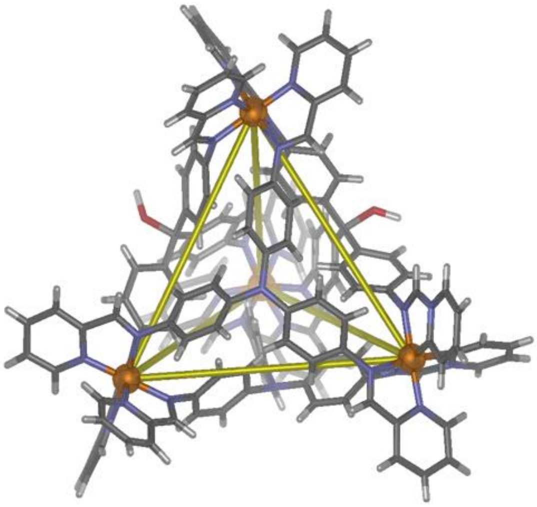

Figure S12. Schematic representation of the crystal structure of one of the [Co_4**L**$^A_2$**L**$^B_2$] cages present in the statistical mixture.

3.4) Ligand pyramidalization

Guest binding may be hindered by the lesser degree of pyramidalization in nitrogen-centered **L**^{A} (incorporated into **1**) than in hydroxymethyl-centered **L**^{B} (incorporated into **2**), as measured by the distances between the central C or N atom of the ligand and the plane defined by the three closest Co^{II} centers in **1** and **2** (1.9 Å and 2.4 Å, respectively, Table S1).

Cage 1

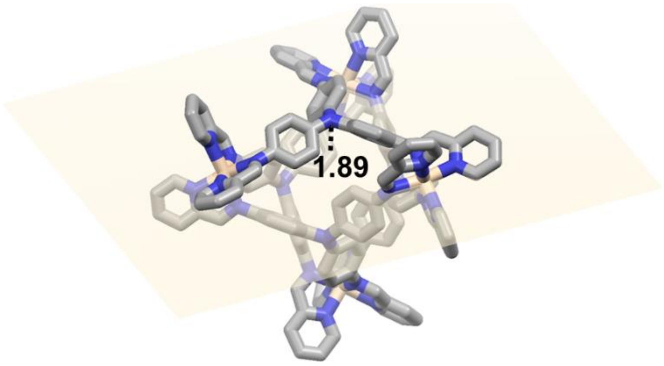

Figure S13. Distances between the central N of the ligands and the plane (in yellow) defined by three adjacent Co^{II} atoms in the crystal structures of **1**.

Cage 2

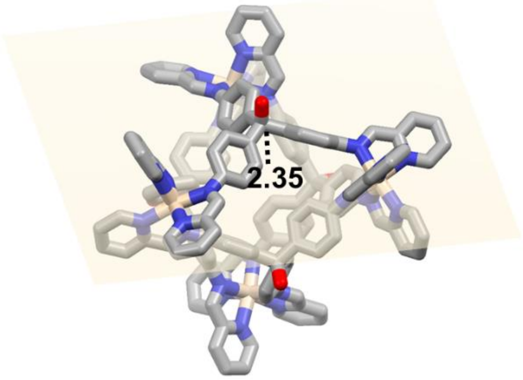

Figure S14. Distances between the central C of the ligands and the plane (in yellow) defined by three adjacent Co^{II} atoms in the crystal structures of **2**.

Table S1. Distances measured for **1** and **2**, with average and standard deviations.

Plane	Distances in 1 / Å	Average for 1 / Å	Standard Deviation for 1 / Å	Distances in 2 / Å
**1**	2.373	2.437	0.102	1.89
**2**	2.350			1.89
**3**	2.576			1.89
**4**	2.448			1.89

3.5) Cavity Volume Calculations

Cavity volumes of the crystal structures of **1** and **2** were calculated with VOIDOO software[13] using a probe radius of 1.4 Å. (Figure S15). The volumes were found to be 58.8 ± 0.8 $Å^3$ and 56.0 ± 0.8 $Å^3$ for **1** and **2**, respectively. Cage **2** was found to have a slightly *smaller* cavity as a consequence of its central phenylene rings adopting an orientation that protrudes more into the central cavity.

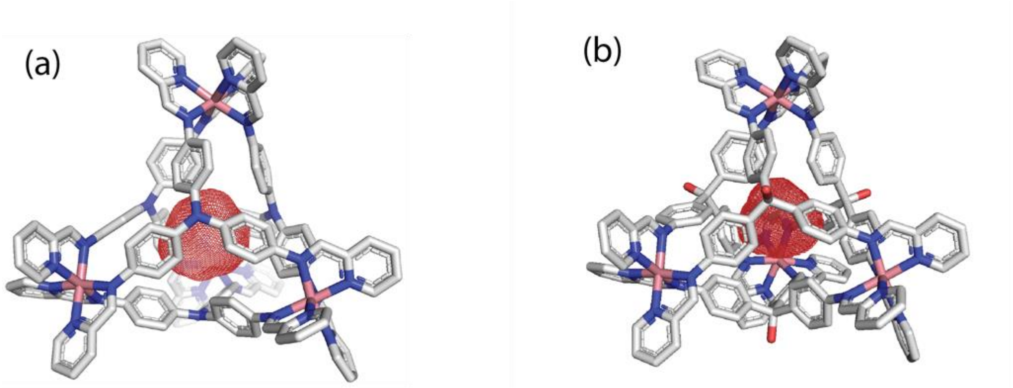

Figure S15. VOIDOO cavity spaces for cages a) **1** and b) **2** shown in red (probe radius 1.4Å).

VOIDOO calculations are run on rigid macromolecules, however, cages **1** and **2** are flexible with internal cavities that can shrink or expand slightly to accommodate guests as necessary through reorientation of the ligand phenyl rings. Thus, the cavity volumes calculated by this method are likely to be a lower bound.

Table S2. Guest volumes calculated with B3LYP/6-31G*. The volume here is taken as the space enclosed by the surface with the electron density equal to 0.002 electrons/$Å^3$, which typically covers about 99% of the electron density. Percent occupancy based on VOIDOO calculation volumes (supra vida).

Guest	Guest volume, /$Å^3$	% volume occupancy of 1, /$Å^3$	% volume occupancy of 2, /$Å^3$
Br^-	28.1	48	50
I^-	34.8	59	62
BF_4^-	54.8	93	98
ClO_4^-	55.4	94	99
CH_3CN	48.7	83	87
$(CF_3SO_2)_2N^-$	117.7	–	–

The volume of mononuclear species Br^- and I^- are about half of the calculated cavity volumes. Since cage **2** was observed to encapsulate these anions, it seems reasonable that cage **1** should as well. Encapsulation of BF_4^- and ClO_4^- approaches 100% and appears to violate Rebek Rule.[14] However, the original rule was quantified only with neutral guests and neutral host devoid of additional factors such as hydrogen bonding or coulombic attraction. The encapsulation of such large guests in **2** implies that cage **1** ought to be able to encapsulate them as well based solely on size considerations.

Acetonitrile has a volume smaller than that of perchlorate but does not bind well within **2** (K_b = 0.0072 ± 0.0004 M^{-1}) presumably because the linear shape of it does not fit the spherically shaped cavity. Moreover, its lack of negative charge likely decreases affinity, which would otherwise lead to stronger binding with the 8^+ charged host. The very weak binding of solvent to **2** is nevertheless observed due to the high concentration of acetonitrile (~20 M). Triflimide is too large to show any internal binding with either of these hosts.

3.6) Calculation of Molecular Orbitals and Electrostatic Potentials

We hypothesize that electronic effects may contribute to the absence of anion binding in **1**. The geometry, molecular orbitals (MOs), and electrostatic potentials (EP) of $\mathbf{L^A}$ and the corresponding cage **1** were calculated at the PM6 level of theory and visualized using the Spartan16 software (Figure S16). Since we are primarily interested in the qualitative pictorial representations that can offer insights into molecular interactions, this level of theory is justified, especially since the cage cannot be readily calculated at higher levels (e.g. DFT).

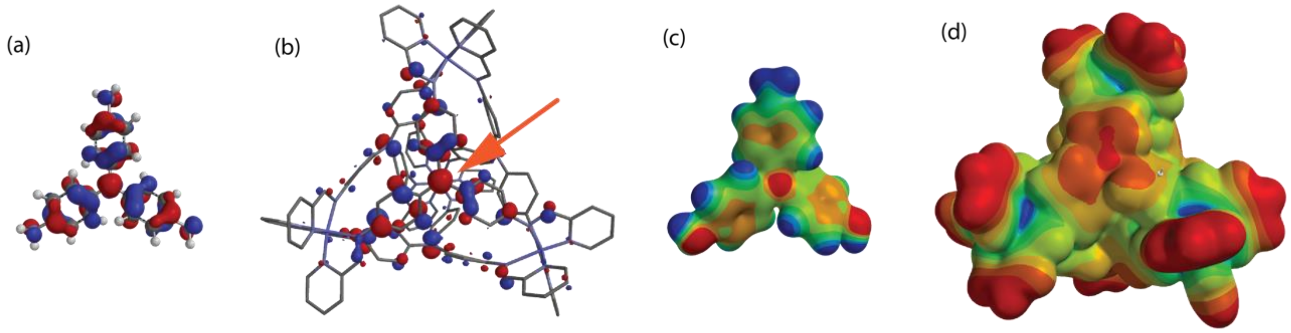

Figure S16. (a) Representation of the highest occupied molecular orbital for $\mathbf{L^A}$. (b) One of the four ligand-type MO for cage **1**. Orange arrow indicates the p orbital on the central nitrogen. (c) Electrostatic potential mapped onto the electron density surface for $\mathbf{L^A}$. (d) Electrostatic potential for cage **1**.

Several computational approaches can be used to gauge intermolecular interactions. First, we considered frontier orbitals, which typically focus on HOMO-LUMO interactions to predict chemical outcomes. In cage **1** however we were interested in identifying destabilizing HOMO-HOMO interactions that could prevent binding of anions. The HOMO for ligand $\mathbf{L^A}$ showed a large coefficient on the central N atom in addition to significant coefficients on the adjacent aryl rings (Figure S16 a).

The HOMO of cage **1** was more complicated. The top 16 occupied orbitals (not shown) are all located on the *d*-type orbitals at the four Co^{II} atoms. Those metal centers were inferred to be too remote to interact directly with guests. However, the first molecular orbitals located on the ligand (Figure S16 b) showed large coefficients at the central N with less delocalization to adjacent aryl rings due to the additional propeller-like twist enforced by steric constrains within the cage. Thus, interaction between anionic guests and the four ligand-type MOs predicts the destabilization of these guests within cage **1**.

Alternatively, one can gauge host-guest interactions through electrostatic potential maps. The electrostatic potential map of the electron density surfaces gave the energy of interaction with a positive point charge. Regions indicated in red attract positive charge and conversely repel anions. The large negative potential of the ligand's central N seen in red (Figure S16 c) indicated that the region of highest destabilization occurred directly at its center. In the case of cage **1** (Figure S16 d), anions occupying the central cavity would be destabilized by electrostatic repulsions from all four ligand faces. The central nitrogen atoms were nearly planar indicating that approximately half its electron density was directed toward the center of the cavity, thus creating an electron-rich microenvironment that disfavors binding of anions.

We also sought alternative explanations for the encapsulation pattern seen in **1** and **2**, namely that cage **2** displayed additional favorable host-guest interactions which were absent in cage **1**. For example, there could be a favorable HOMO-LUMO interaction between a filled orbital on the anion and an empty antibonding $\sigma_{C\text{–}O}$ orbital on the ligand. Figure S17 shows the LUMO for the free ligand $\mathbf{L^B}$.

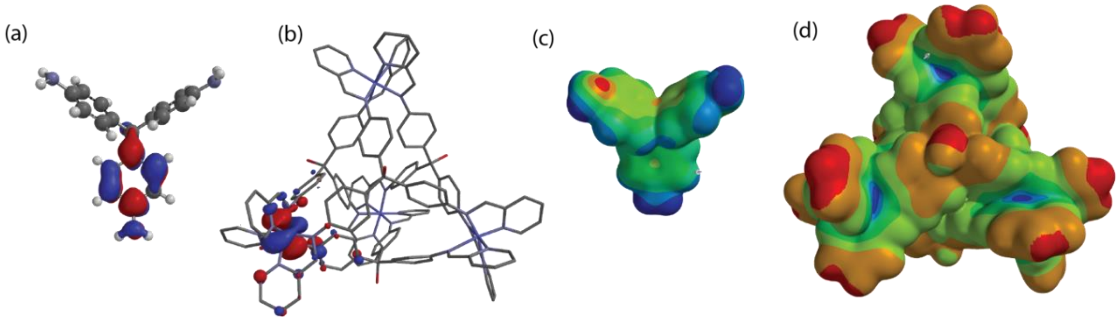

Figure S17. (a) Representation of the LUMO for $\mathbf{L^B}$. (b) The LUMO from one of the many unoccupied MO located on the metal d orbitals of cage **2**. (c) Electrostatic potential mapped onto the electron density surface for $\mathbf{L^B}$. (d) EP surface of cage **2**.

The LUMO of the ligand $\mathbf{L^B}$ is localized primarily on the phenyl ring rather than the central carbon (Figure S17 a), as is anticipated from the energies of the π* and σ* orbitals. The first ten LUMOs of cage **2** are centered on the Co^{II} atoms (Figure S17 b). Spartan software was not able to find a LUMO orbital involving the C–O σ*. This implies that the LUMO is energetically inaccessible and contributes little to any potential HOMO-LUMO interactions. The electrostatic potential on the corresponding ligand (Figure S17 c) shows that the central carbon atom is buried behind the C–H bonds of two adjacent phenyl rings. The corresponding σ* orbital is thus inaccessible. Finally, a strong interaction between the guest such as ClO_4^- and the center of the ligand would lead to the guest orienting its Cl–O bond toward the central carbon, i.e. in a head-to-tail arrangement of their dipoles. However, the crystal structure of **2** (Figure S11) indicates that the Cl–O bond is oriented toward the corners of the tetrahedron and not the faces. While there might be some non-traditional hydrogen bond interaction with the interior CHs, there is little computation evidence for any stabilizing interaction between anions and cage **2**.

Overall, computations suggest that the encapsulation or lack of thereof between **1** and **2** is neither due to a size effect, nor a special stabilization with **2**, but rather a destabilization due to the lone pair of the central N atom in cage **1**.

4) Host-guest studies

4.1) Guest binding in 1

The formation of host-guest complexes between cage **1** and different anions was studied when the guest anions were added following the self-assembly process. To do so, $TBABF_4$ (20 equiv.), $TBAClO_4$ (20 equiv.), TBAI (4 equiv.) or TBABr (2 equiv.) were added to a solution of **1 (**0.5 mM). Addition of more than 4 or 2 equiv. of TBAI or TBABr respectively resulted in precipitation of the samples. The samples were left to equilibrate for 4 days at 70 °C.
No shift of the cage's peak following interactions in fast exchange on the NMR time scale, nor appearance of a new set of signals corresponding to a host-guest complex in slow exchange was observed in the NMR, indicative of the lack of binding of these anions in the cage's cavity.

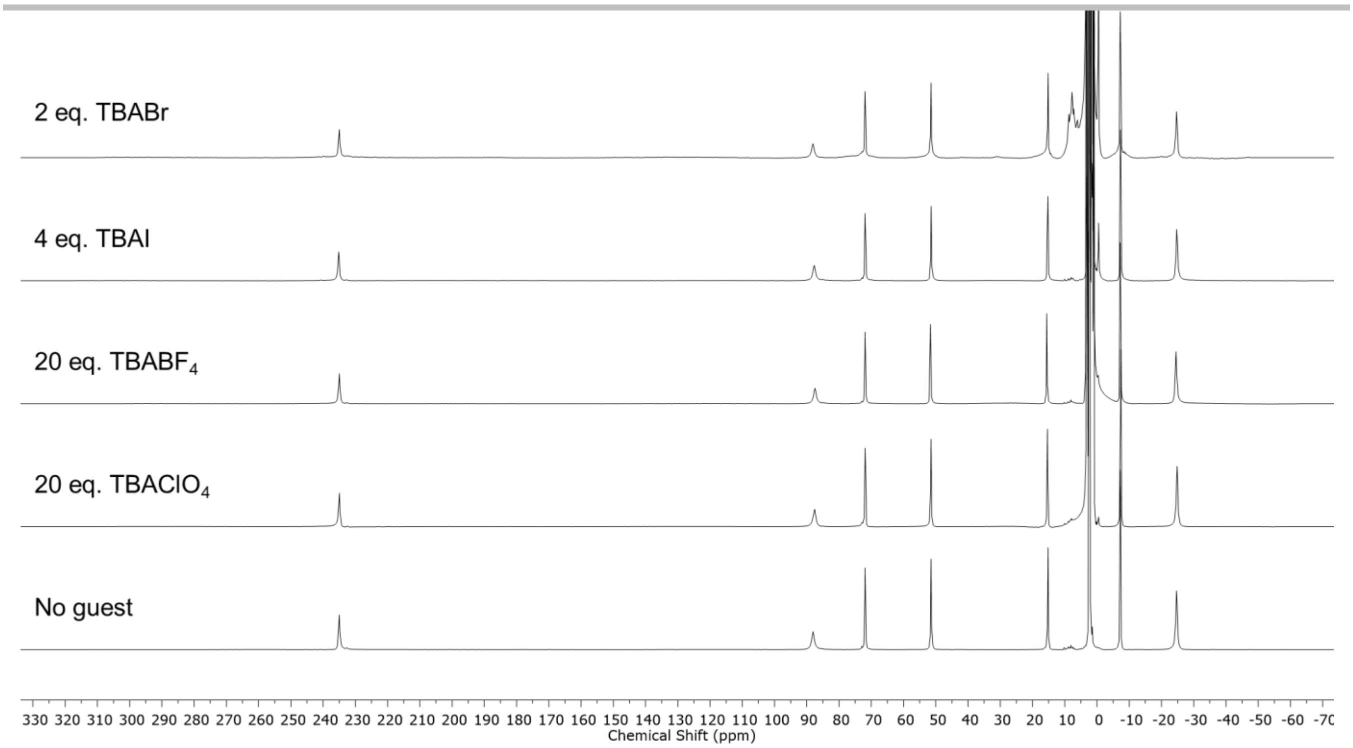

Figure S18. Wide sweep ^{1}H NMR spectra (400 MHz, 298 K, CD_3CN) of **1** after addition of $TBABF_4$ (20 equiv.), $TBAClO_4$ (20 equiv.), TBAI (4 equiv.) and TBABr (2 equiv.).

The formation of host-guest complexes between cage **1** and different anions was also studied when the guest anions were present during the self-assembly process. To do so, tris(4-aminophenyl)amine **A** (2.0 mg, 6.9 µmol, 1.0 equiv.), 2-formylpyridine (2.2 mg, 20.7 µmol, 3.0 equiv.) and $Co(X_2)_2$ (6.9 µmol, 1.0 equiv.) were combined in CD_3CN (0.5 mL) in a sealed 2 mL reaction tube. The solution was stirred and heated to 70 °C for 18 h. The samples were then analyzed by ^{1}H and ^{19}F NMR.

For X^{-}= NTf_2^{-}, BF_4^{-} or ClO_4^{-}, no differences between the corresponding ^{1}H NMR spectra were observed, consistent with the absence of fast guest exchange on the NMR timescale. Furthermore, no additional signals corresponding to a host-guest complex in the ^{1}H NMR spectra, nor peaks corresponding to encapsulated guest in the ^{19}F NMR spectra, were observed.

In the cases where X^{-}= Br^{-} or I^{-}, no signals corresponding to cage complexes were observed in the ^{1}H NMR and only small signals for subcomponents in the diamagnetic region were observed. Solids were also observed in the samples. We inferred that any complexes formed were insoluble in acetonitrile.

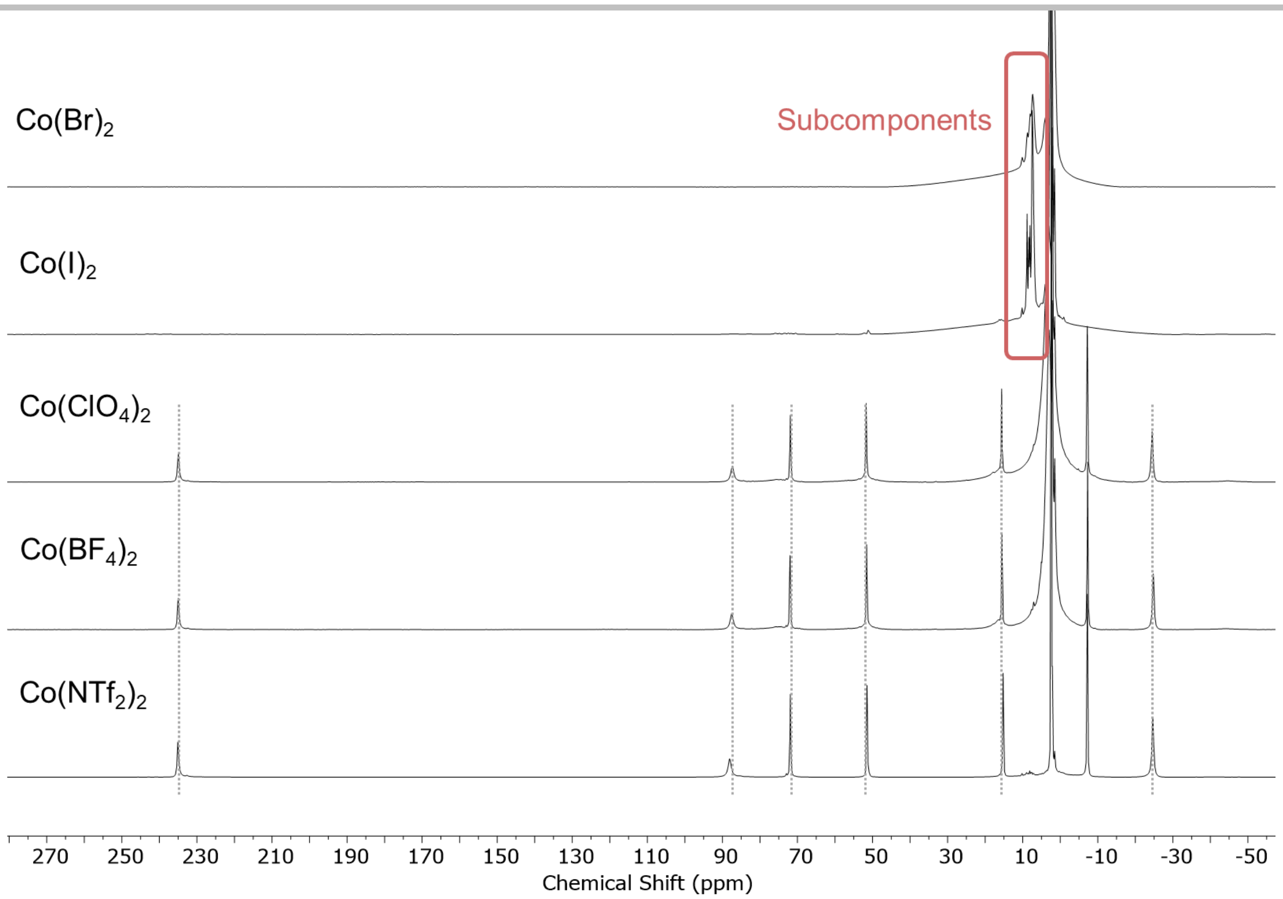

Figure S19. Wide sweep ^{1}H NMR spectra (400 MHz, 298 K, CD_3CN) of **1** formed from $Co(NTf_2)_2$, $Co(BF_4)_2$, $Co(ClO_4)_2$, and the corresponding products formed from CoI_2 and $CoBr_2$ (bottom to top).

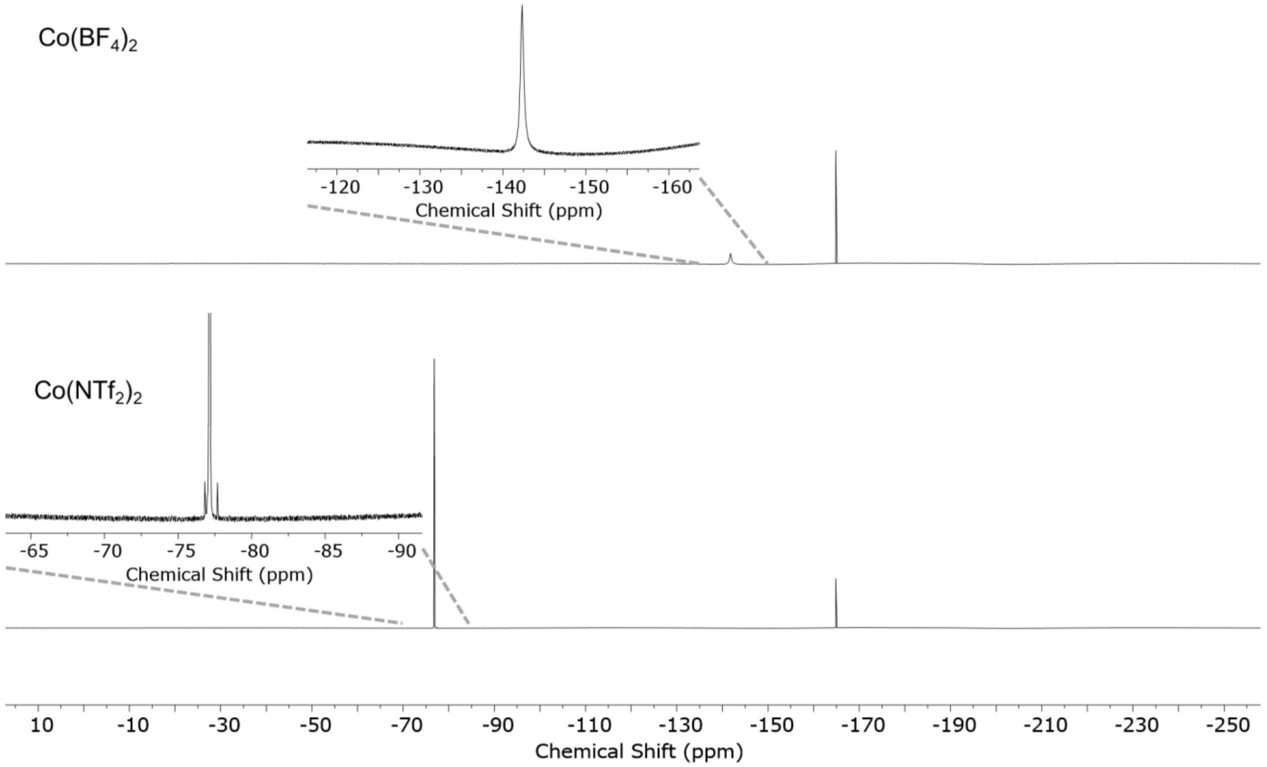

Figure S20. Wide sweep ^{19}F NMR spectra (376 MHz, 298K, CD_3CN) of **1** formed from $Co(NTf_2)_2$ or $Co(BF_4)_2$ with inserts showing the regions corresponding to signals for BF_4^- or NTf_2^-. The spectra were referenced using a capillary containing C_6F_6 in CD_3CN ($\delta = -164.90$ ppm).

4.2) Guest binding in 2

Binding of acetonitrile

A solution of cage **2** (3 mM) was prepared in CH_3CN according to the procedure described in section S2.2. A capillary of CD_3CN was added to the NMR tube containing **2** in CH_3CN in order to allow locking.

The ^{1}H NMR spectrum of **2** in CH_3CN was compared to that of the same cage in CD_3CN. Solvent suppression (single presaturation at 2.03ppm) was used to observe the region close to the solvent peak, however, due to the paramagnetic character of the sample, this led to a strong decrease in the proton signals of **2**. Both spectra had identical resonances, except for an additional peak at –0.9 ppm in CH_3CN which was attributed to the encapsulated CH_3CN.

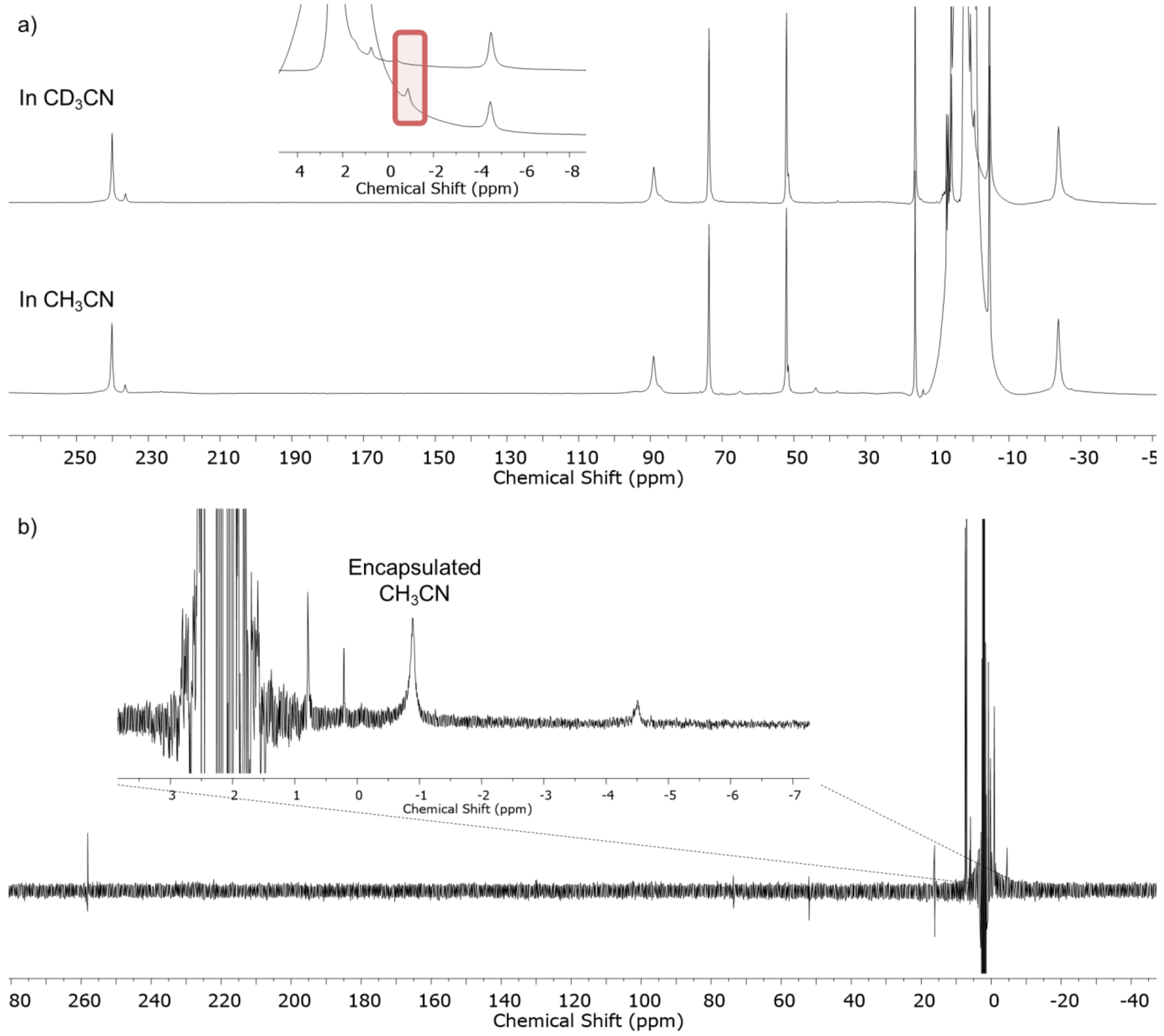

Figure S21. a) Wide sweep ^{1}H NMR spectra (400 MHz, 298 K) of **2** in CD_3CN (top) and CH_3CN (bottom) showing the additional peak at 0.9 ppm in the second spectrum. b) Wide sweep ^{1}H NMR spectrum (400 MHz, 298 K, CH_3CN) after solvent suppression.

Binding of ClO_4^-

TBAClO_4 was titrated into a solution of cage **2** (1.0 mM). The samples were left to equilibrate for 24 h at 70 °C after each addition of TBAClO_4 before collecting the spectrum.

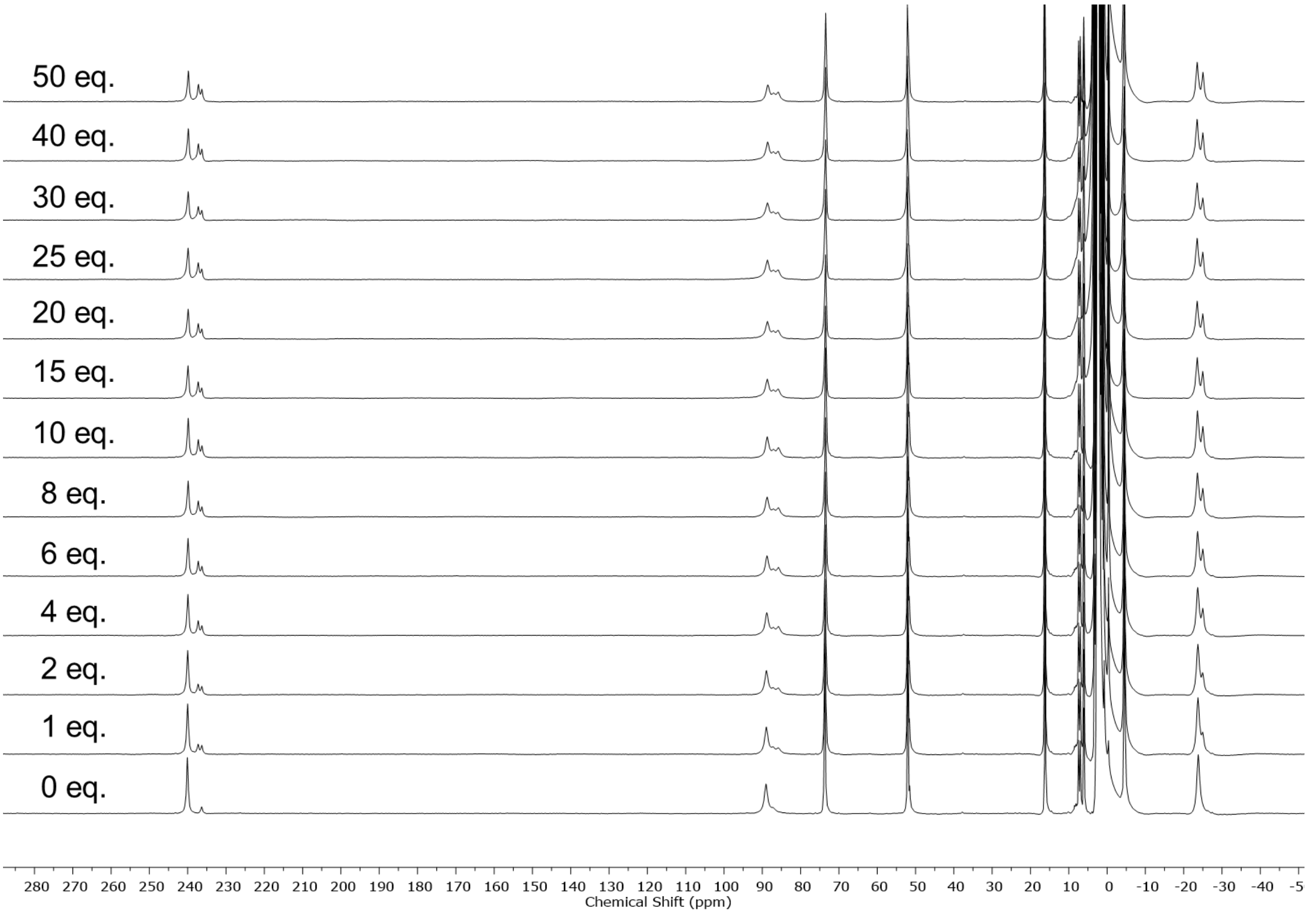

Figure S22. Wide sweep ^{1}H NMR spectra (400 MHz, 298 K, CD_3CN) of cage **2** after titration of TBAClO_4 (equivalents of anions labelled on the spectra)

See sections 5.2 and 5.3 for more details on the calculation of the binding constants.

Binding of BF_4^-

TBABF_4 was titrated into a solution of cage **2** (1.0 mM). The samples were left to equilibrate for 24 h at 70 °C after each addition of TBABF_4 before collecting the spectrum.

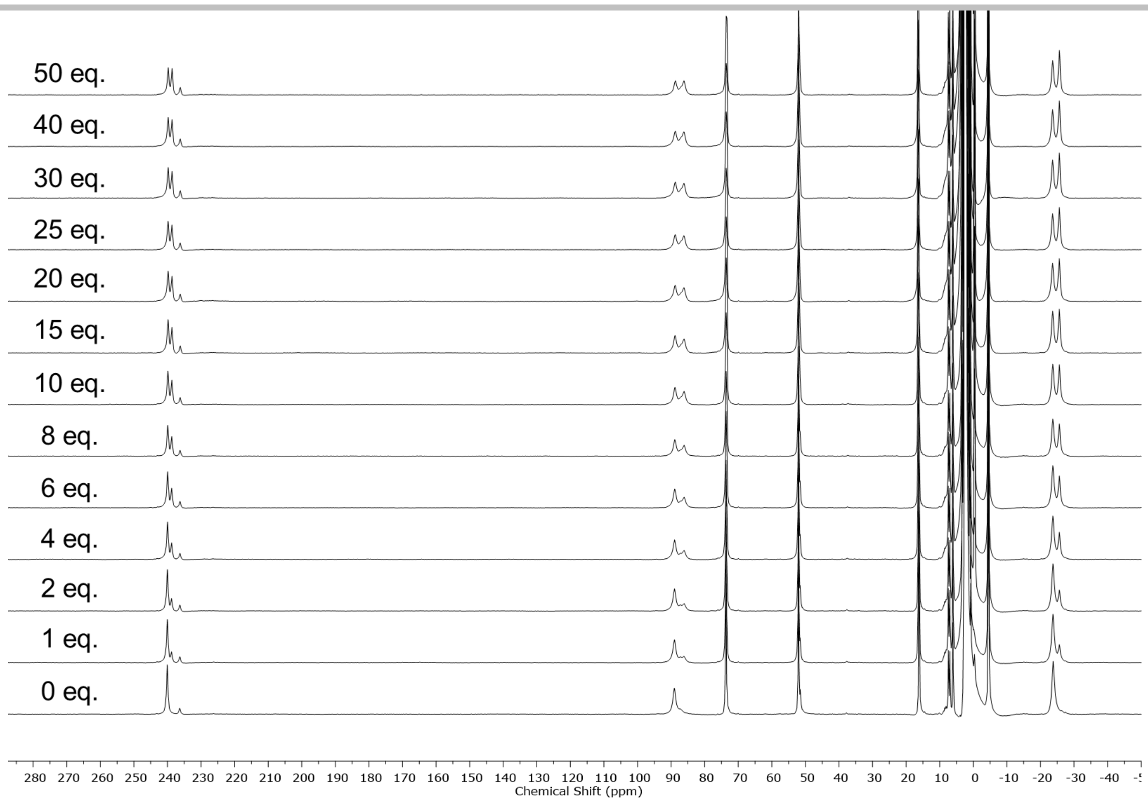

Figure S23. Wide sweep ^{1}H NMR spectra (400 MHz, 298 K, CD_3CN) of cage **2** after titration of $TBABF_4$ (equivalents of anions labelled on the spectra)

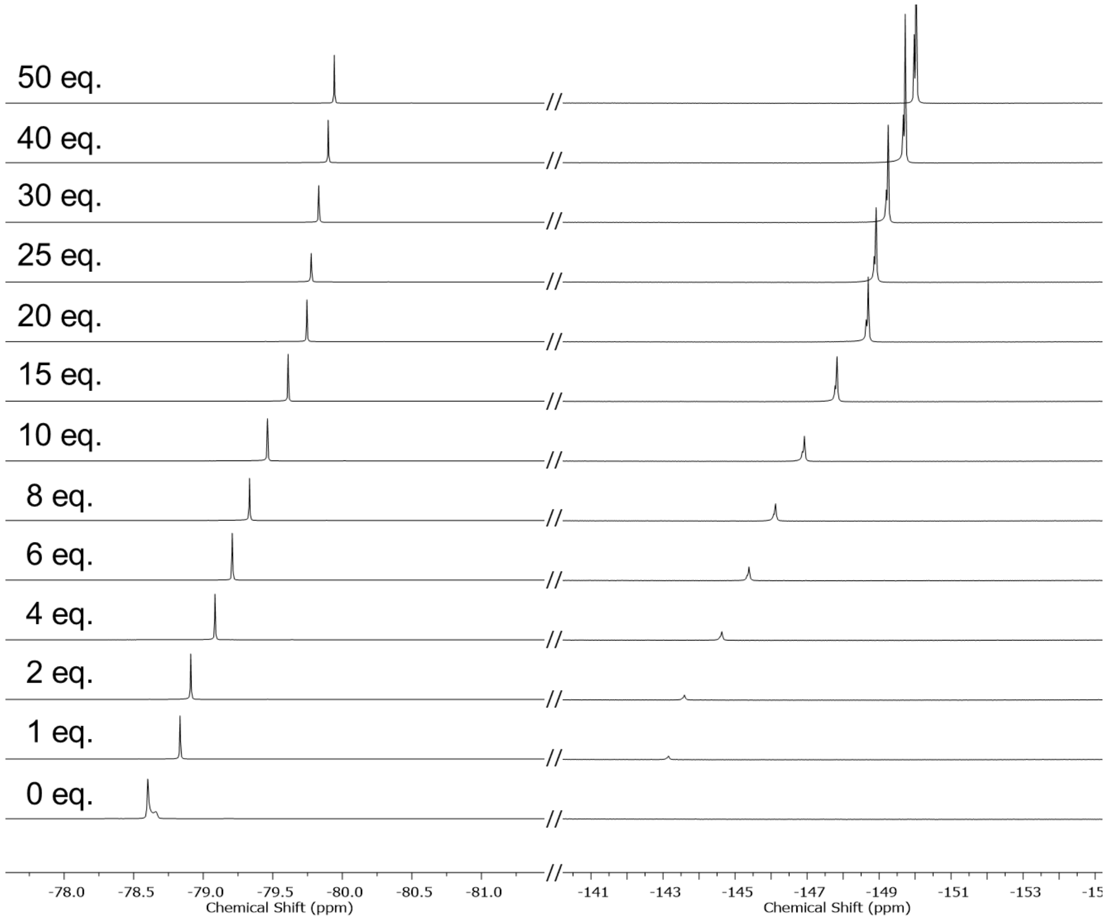

Figure S24. ^{19}F spectra (376 MHz, 298 K, CD_3CN) of cage **2** after titration of $TBABF_4$ (equivalents of anions labelled on the spectra) showing the shift of the NTf_2^- and BF_4^- signals.

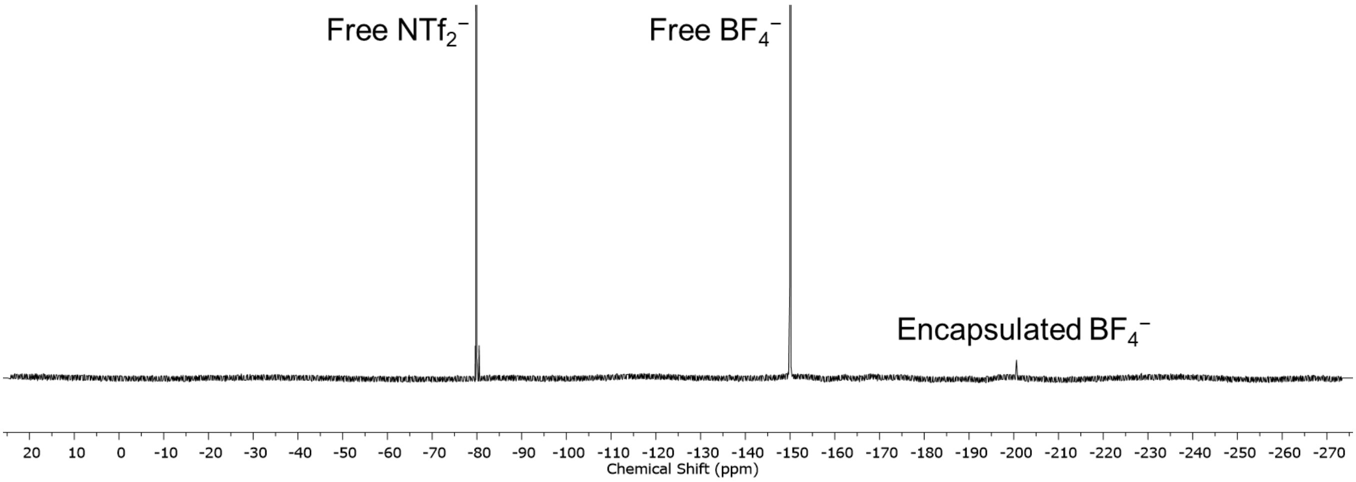

Figure S25. ^{19}F spectrum (376 MHz, 298 K, CD_3CN) of cage **2** after addition of $TBABF_4$ (50 equiv.).

The free NTf_2^- and BF_4^- peaks were observed to shift, which was attributed to changes in the iconicity of the solution. The shift of the signal for the encapsulated BF_4^- did not show any changes.
See sections 5.2 and 5.3 for more details on the calculation of the binding constants.

Binding of I⁻

TBAI was titrated into a solution of cage **2** (1.0 mM). The samples were left to equilibrate for 24 h at 70 °C after each addition of TBAI before collecting the spectrum.

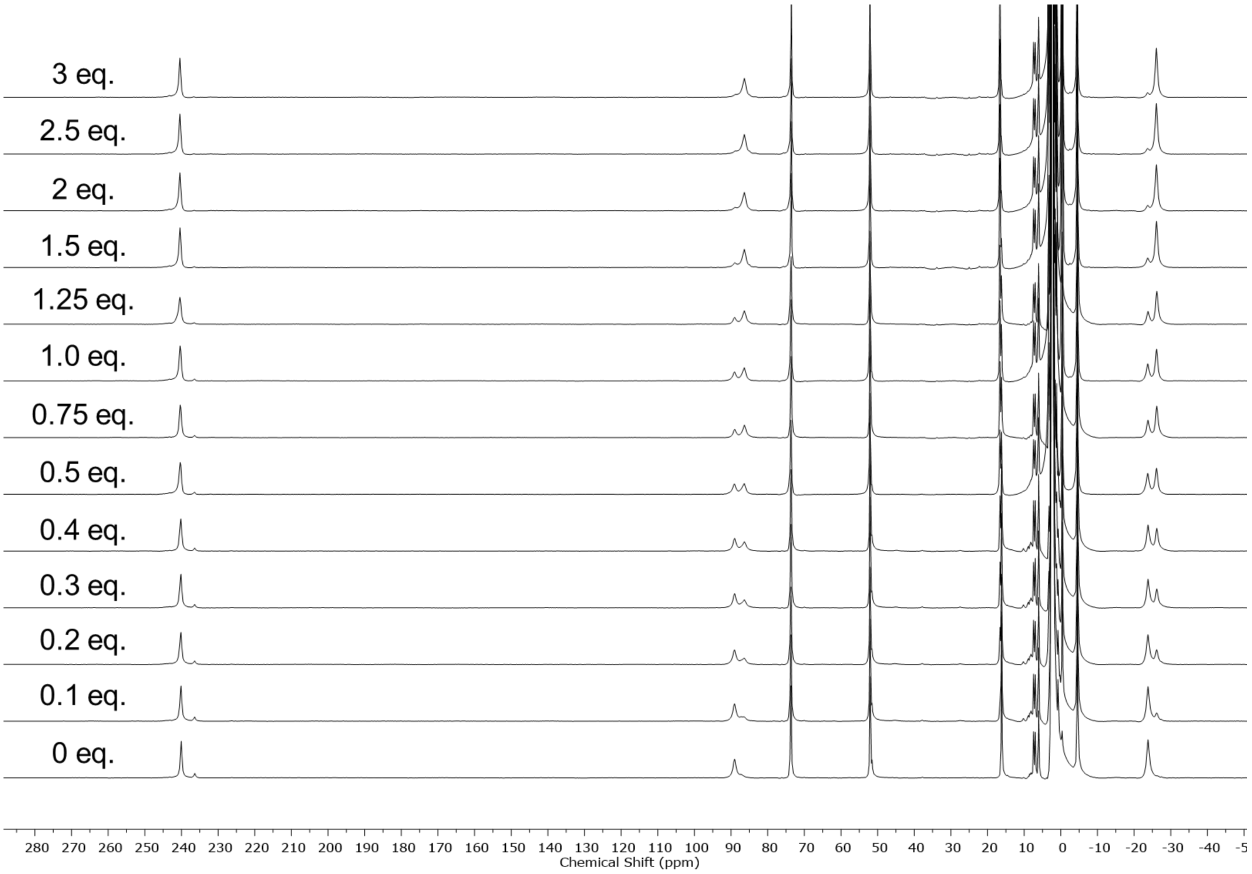

Figure S26. Wide sweep ^{1}H NMR spectra (400 MHz, 298 K, CD_3CN) of cage **2** after titration of TBAI (equivalents of anions labelled on the spectra)

See sections 5.2 and 5.3 for more details on the calculation of the binding constants.

Binding of Br⁻

TBABr was titrated into a solution of cage **6.10** (1.0 mM). The samples were left to equilibrate for 24 h at 70 °C after each addition of TBABr before collecting the spectrum.

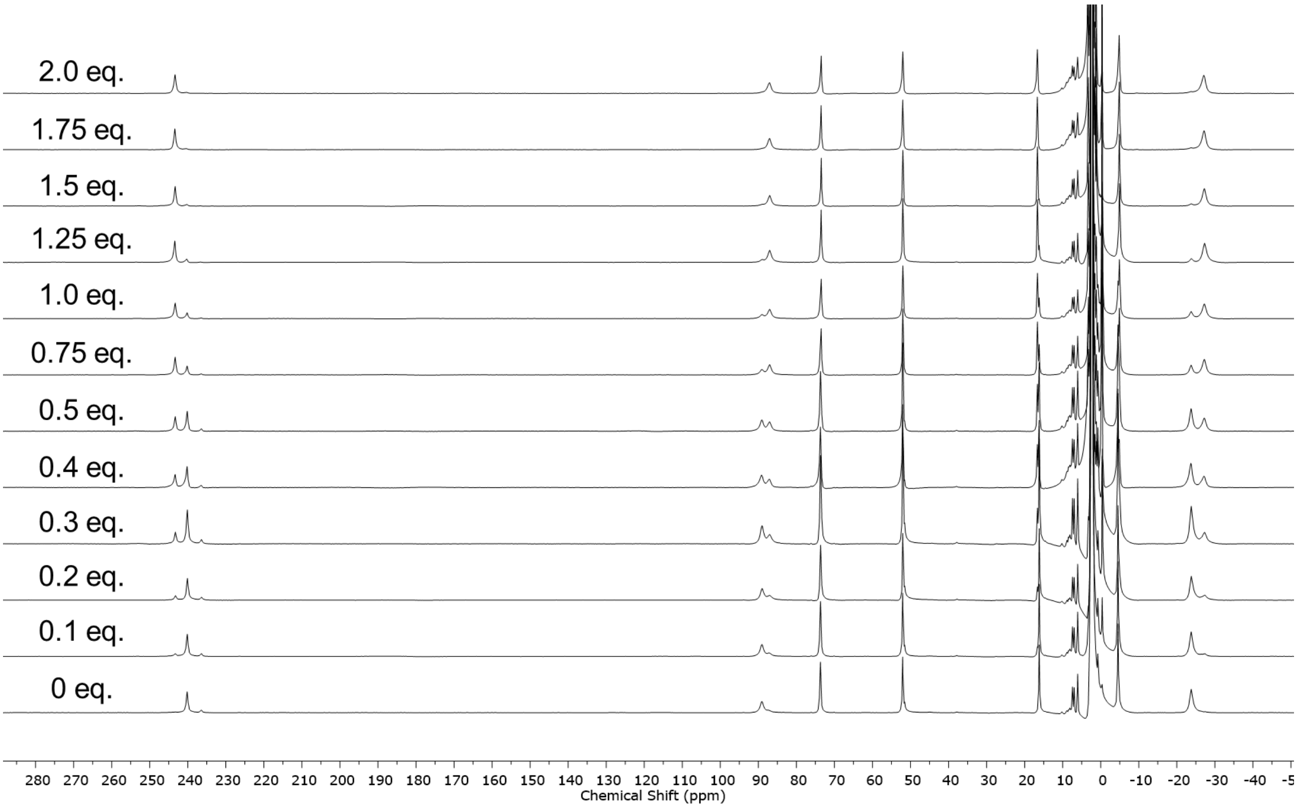

Figure S27. Wide sweep ^{1}H NMR spectra (400 MHz, 298 K, CD_3CN) of cage **2** after titration of TBABr (equivalents of anions labelled on the spectra).

See sections 5.2 and 5.3 for more details on the calculation of the binding constants.

Competitive binding of anions

In order to establish the relative strength of binding of anions, competitive binding was performed. $TBAClO_4$, $TBABF_4$, TBAI and TBABr (1 equiv. each) were added subsequently to a solution of cage 2 (1 mM).

The association strength of the different anions was inversely correlated to their size, ie. $Br^{-} > I^{-} >> BF_4^{-} \geq ClO_4^{-}$.

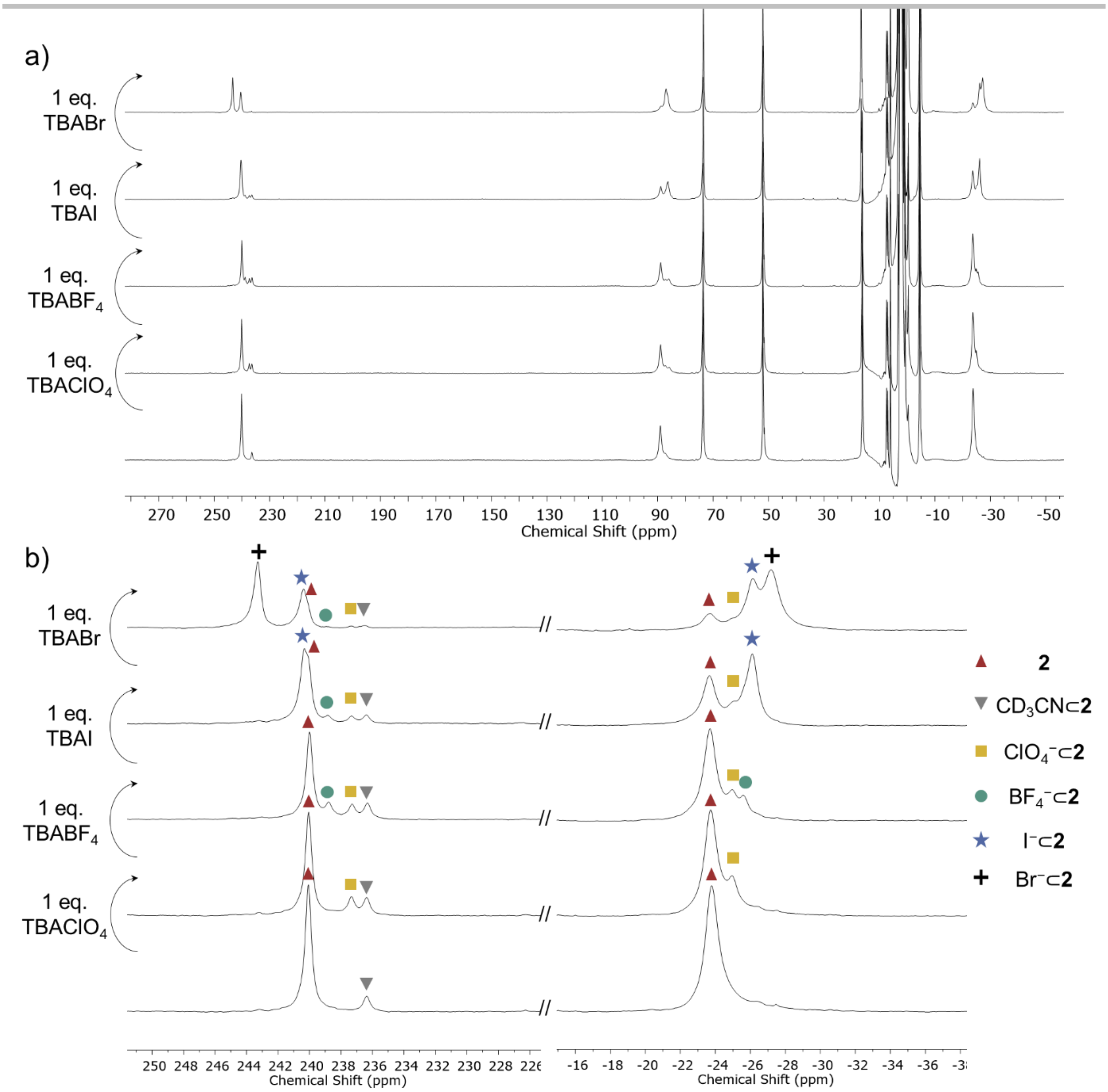

Figure S28. a) Wide sweep ^{1}H NMR spectra (400 MHz, 298 K, CD_3CN) of **2** after addition of TBAI, TBABr, $TBABF_4$ or $TBAClO_4$ (1 equiv.) showing new sets of peaks for the host-guest complexes. b) Zoom into the 250 to 230 ppm and –16 to –36 ppm region of the ^{1}H NMR spectra and assignment of the signals for **2** (red triangle), $CD_3CN{\subset}$**2** (inverted grey triangle), $ClO_4^-{\subset}$**2** (yellow triangle), $BF_4^-{\subset}$**2** (green circle), $I^-{\subset}$**2** (blue star) and $Br^-{\subset}$**2** (black cross).

5) Binding modes in cage 2

5.1) Role of TBA^+

Cage **2**$(BF_4)_8$ was synthesized directly from $Co(BF_4)_2$ according to the procedure described in section 2.2 and $TBABF_4$ (8 equiv.) was added to **2**$(NTf_2)_8$, giving two different solutions of **2**, containing equal amounts of BF_4^- but different amounts of TBA^+ and NTf_2^- (respectively 0 equiv. and 8 equiv.).

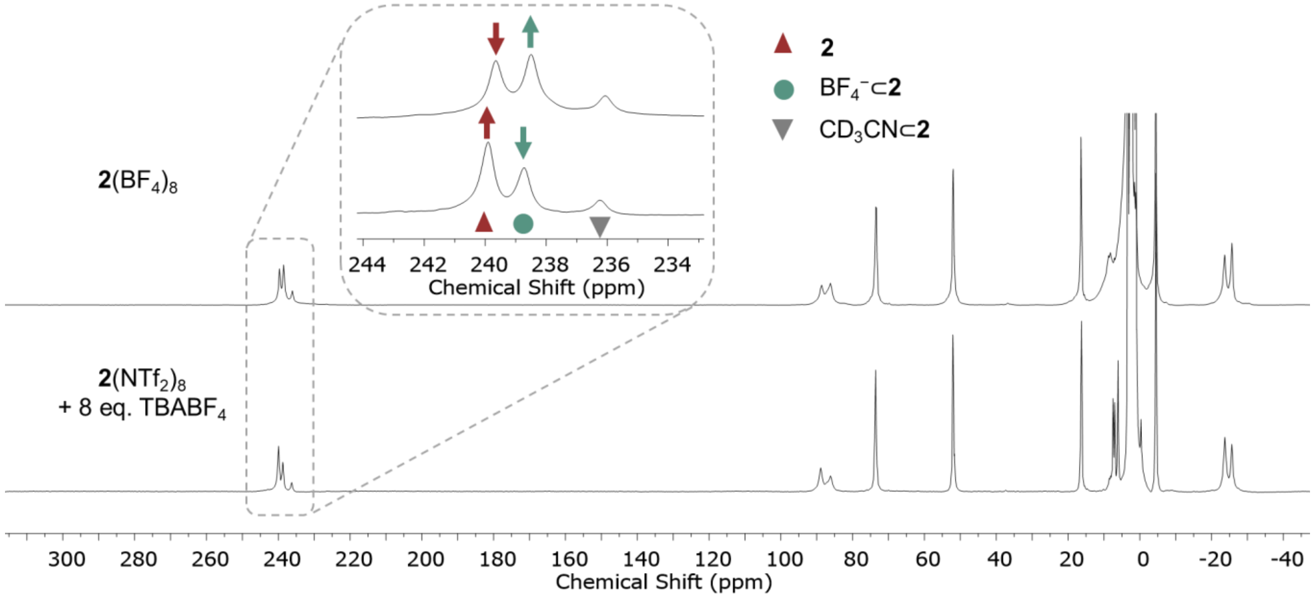

Figure S29. Wide sweep ^{1}H NMR spectra (400 MHz, 298 K, CD_3CN) of **2**$(NTf_2)_8$ to which was added 8 equiv. of $TBABF_4$ (bottom) and **2**$(BF_4)_8$ (top).

Close examination of the ^{1}H NMR spectra revealed that the amount of encapsulated BF_4^- was greater in the case where neither TBA^+ nor NTf_2^- were present.

Either $TBANTf_2$ (50 equiv.) or $TBABF_4$ (50 equiv.) were added to a solution of **2**$(BF_4)_8$.

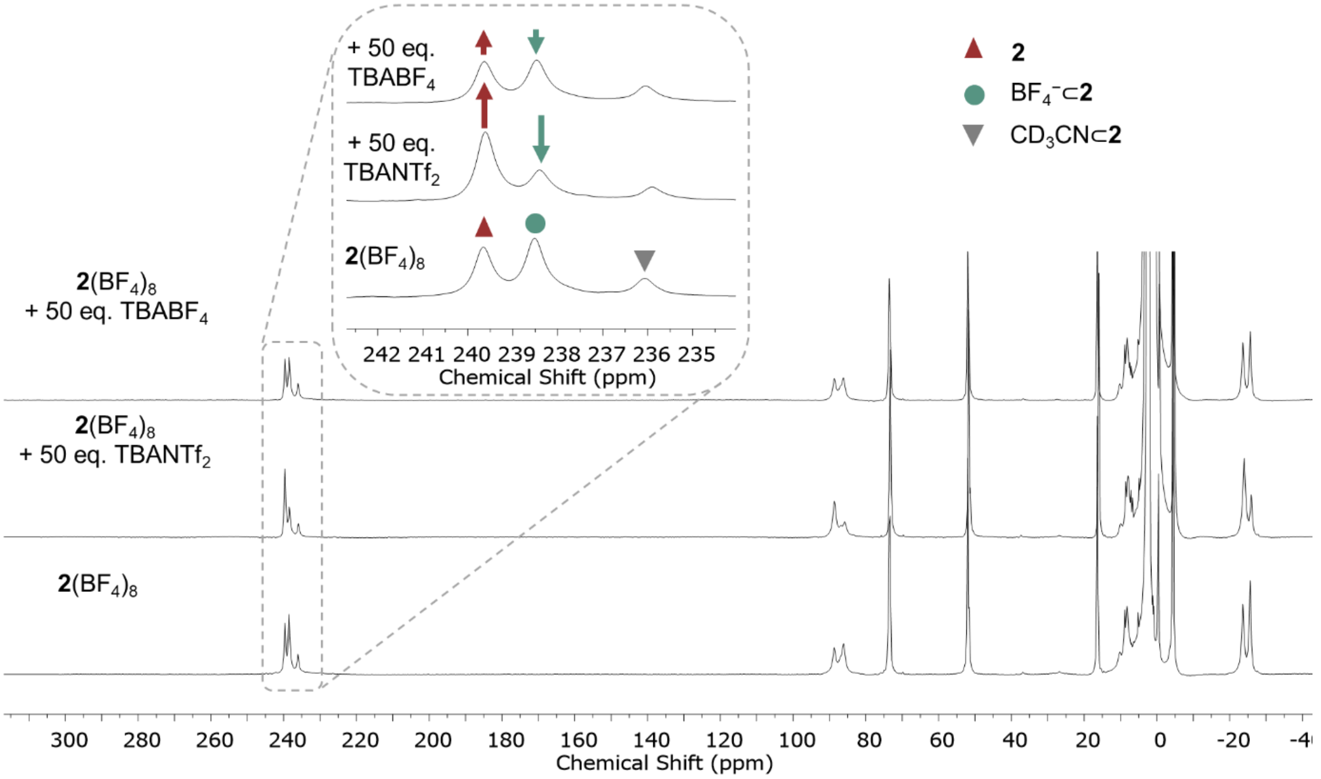

Figure S30. Wide sweep ^{1}H NMR spectra (400 MHz, 298 K, CD_3CN) of **2**$(BF_4)_8$ before (bottom) and after addition of either 50 equiv. of $TBANTf_2$ (middle) or $TBABF_4$ (top).

A drastic decrease in the encapsulated BF_4^- was observed when $TBANTf_2$ (50 equiv.) was added to **2**$(BF_4)_8$. When $TBABF_4$ (50 equiv.) was added to **2**$(BF_4)_8$, the amount of host-guest complex (BF_4^-⊂**2**) decreased as well, despite the large increase in guest concentration. We thus inferred that the association of the TBA^+ cation with the anionic guest limit the formation of the host-guest complex X^-⊂**2**.

5.2) Binding curves and fitting

The changes in the concentration of the host-guest complex ($X^-⊂$**2**, abbreviated HG), host-solvent complex ($CH_3CN⊂$**2**, abbreviated HS), and empty cage (**2**, abbreviated H) as a function of the added guest were described by sets of equations. Based on the different behaviors observed in the titrations, two scenario were defined:

- When the affinity of the guest for cage **2** was superior to the affinity of the guest for TBA^+, the concentration of host-guest complex [HG] converged to unity and the concentration of host-solvent [HS] converged to zero. This was observed when strongly binding anions (TBABr or TBAI) were titrated into a CD_3CN solution of **2**. In this case, the system was described by Equation 1 – 3.

Equation 1

$$[HG] = \frac{[H_t] + [G_t] + \frac{(1 + K_{HS}[S_t])}{K_{HG}} - \sqrt{\left([H_t] + [G_t] + \frac{(1 + K_{HS}[S_t])}{K_{HG}}\right)^2 - 4[H_t][G_t]}}{2}$$

Equation 2

$$[H] = \frac{[H_t] - \frac{1}{2}\left([H_t] + [G_t] + \frac{(1 + K_{HS}[S_t])}{K_{HG}} - \sqrt{\left([H_t] + [G_t] + \frac{(1 + K_{HS}[S_t])}{K_{HG}}\right)^2 - 4[H_t][G_t]}\right)}{(1 + K_{HS}[S_t])}$$

Equation 3

$$[HS] = \frac{K_S[S_t]}{1 + K_S[S_t]}\left([H_t] - \frac{1}{2}\left([H_t] + [G_t] + \frac{(1 + K_{HS}[S_t])}{K_{HG}} - \sqrt{\left([H_t] + [G_t] + \frac{(1 + K_{HS}[S_t])}{K_{HG}}\right)^2 - 4[H_t][G_t]}\right)\right)$$

- When the affinity of the guest for cage **2** was inferior to the affinity of the guest for TBA^+, the concentration of host-guest complex [HG] converged to a value inferior to one and the concentration of host-solvent [HS] remained constant. In this case, the [HS] was fitted to a straight line. A scaling factor (C) was introduced to account for the pairing between TBA and X^-. This was observed when weakly binding anions ($TBABF_4$ or $TBAClO_4$) were titrated into a CD_3CN solution of **2**. The system was described by Equation 4 – 5.

Equation 4

$$[HG] = C\frac{[H_t] - [HS] + [G_t] + \frac{1}{K_{HG}} - \sqrt{\left([H_t] + [G_t] + \frac{(1 + K_{HS}[S_t])}{K_{HG}}\right)^2 - 4[H_t][G_t]}}{2}$$

Equation 5

$$[H] = [H_t] - [HS] - \frac{C}{2}\left([H_t] + [G_t] + \frac{(1 + K_{HS}[S_t])}{K_{HG}} - \sqrt{\left([H_t] + [G_t] + \frac{(1 + K_{HS}[S_t])}{K_{HG}}\right)^2 - 4[H_t][G_t]}\right)$$

Equation 6

$$[HS] = m[G_t] - b$$

where $[S_t]$ represents the total solvent concentration, $[H_t]$ the total host concentration, $[G_t]$ the total guest concentration, K_{HS} the equilibrium constant for solvent binding, K_{HG} the equilibrium constant for guest binding and m and b are constants. C represented the scaling factor, which was added to account for the contribution of the TBA^+ pairing with the different anions.

Fittings of the NMR titration data to Equations 1 – 3 or 4 – 6 were performed simultaneously rather than individually using a Mathematica program, allowing association constants for the different anions to be calculated. More information on the mathematical models is given in Section 5.3.

The Mathematica program is attached as supplementary information.

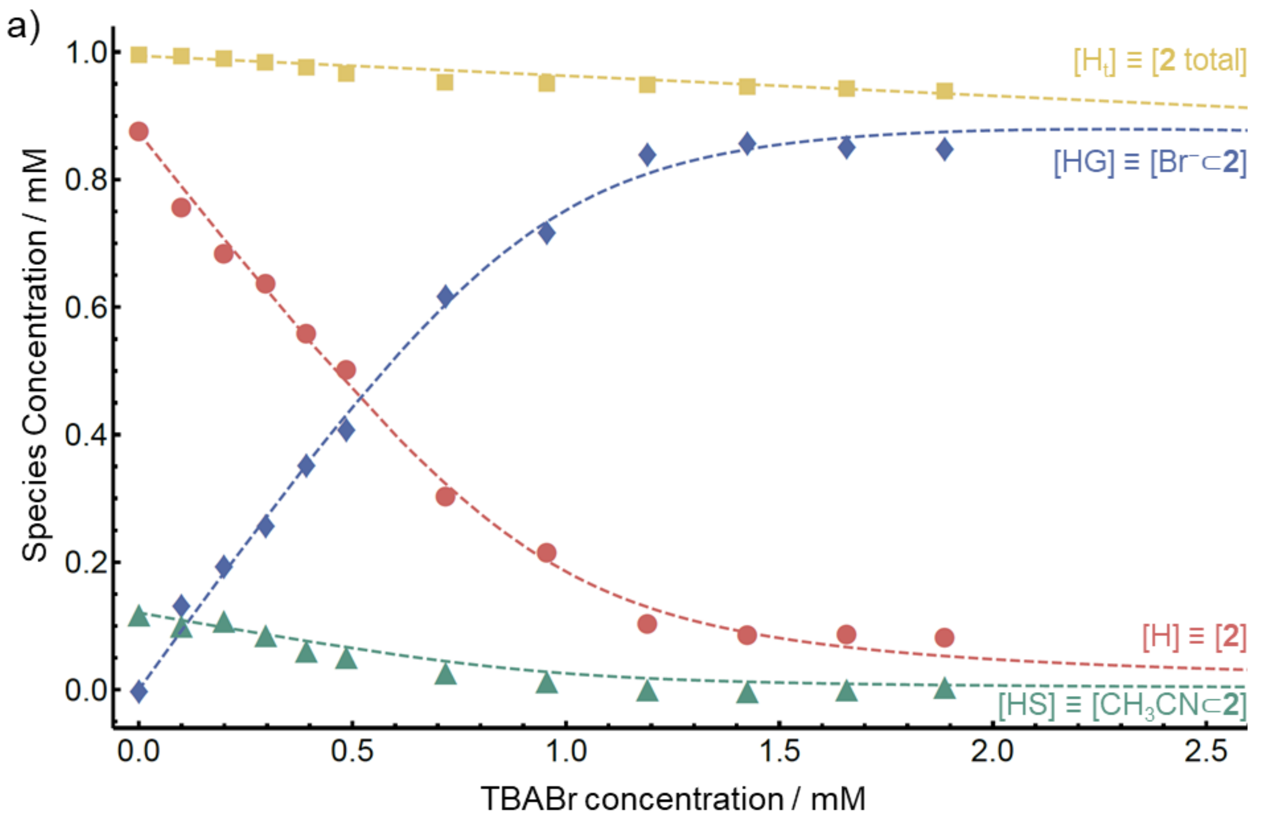

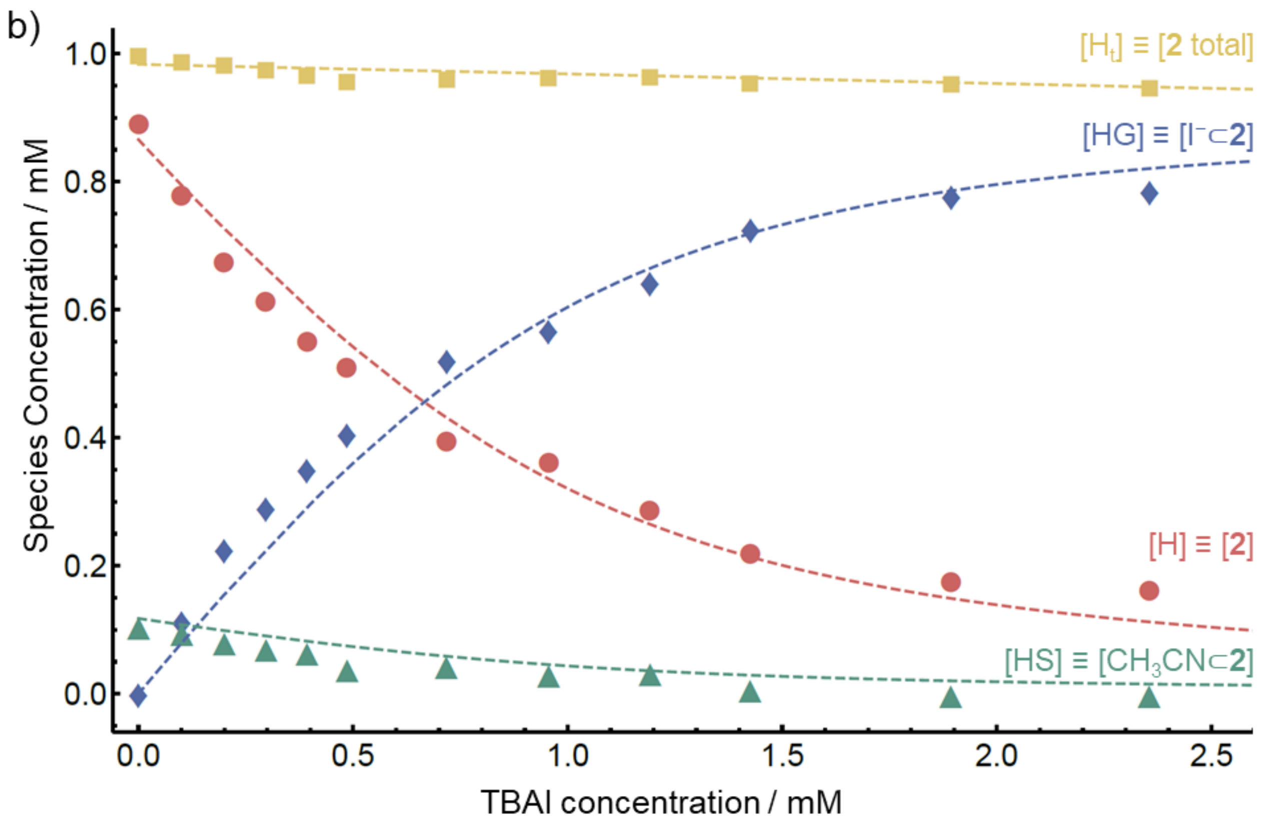

Figure S31. Plot of the concentration of total host (**2** total in yellow), free host (**2** free in red), host-guest complex (X^-⊂**2** in blue) and solvent-host complex (CH_3CN⊂**2** in green) as a function of the concentration of guest (TBAX) for a) X = Br^- and b) I^-. The experimental data obtained from titrations is given by the points (triangles, circles, squares and diamonds) and the fitting obtained is represented by the dotted lines.

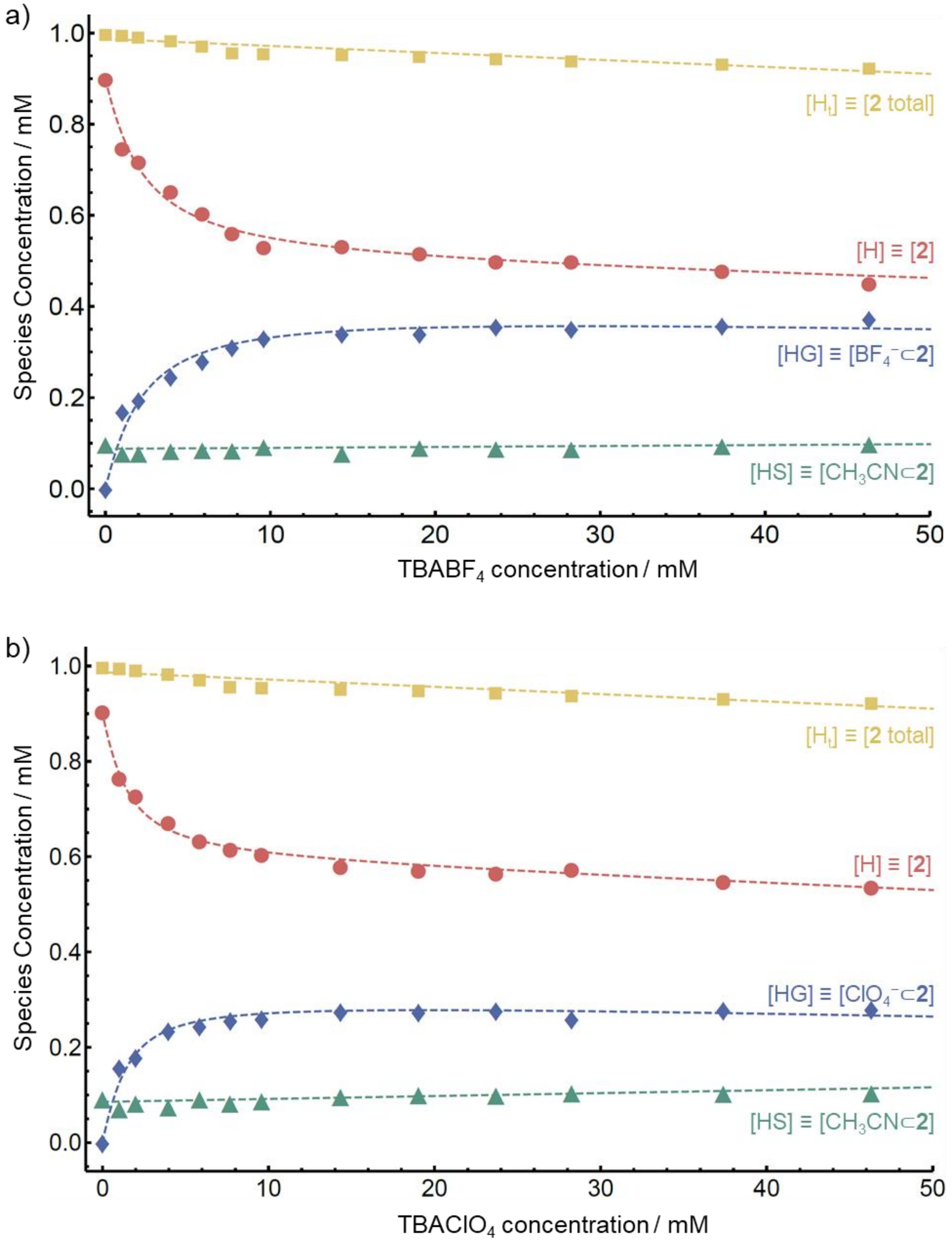

Figure S32. Plot of the concentration of total host (**2** total in yellow), free host (**2** free in red), host-guest complex (X^-⊂**2** in blue) and solvent-host complex (CH_3CN⊂**2** in green) as a function of the concentration of guest (TBAX) for a) X = BF_4^- and b) ClO_4^-. The experimental data obtained from titrations is given by the points (triangles, circles, squares and diamonds) and the fitting obtained is represented by the dotted lines.

Due to dilution effects resulting from the addition of the guest solution during titration, the total host concentration [H_t] decreased and was modelled as a straight line (yellow line). Thus, the host-guest concentration [HG] increased asymptotically to the dilution curve (blue line). Furthermore, unity was not reached in the case of weakly binding anions (BF_4^- and ClO_4^-) due to the competition of TBA^+ for the guest, which was introduced into the system in same amount as the guest. The scaling factor C was thus introduced in the equations to account for the TBA pairing; C was equal to one in the case of Br^- and I^- and corresponded to the saturation concentration, i.e. the asymptote of the [HG] curve for BF_4^- and ClO_4^-. For larger guests (BF_4^- and ClO_4^-), the concentration of encapsulated solvent [HS], did not decrease asymptotically to zero as more guest was added, but remained at 10% occupancy and the binding curves for [HS] were thus fitted to a straight line. This was explained by the weaker binding affinity of these guests which could not displace the solvent, present in large excess. Smaller guests (Br^- and I^-) with stronger binding expelled the weakly bound solvent.
The following values for the association constants of the guests in **2** in the presence of TBA^+ were obtained:

$$K_{Br} = 16000 \pm 1000\ M^{-1}$$
$$K_{I} = 4800 \pm 400\ M^{-1}$$
$$K_{BF_4} = 580 \pm 50\ M^{-1}$$
$$K_{ClO_4} = 1100 \pm 100\ M^{-1}$$
$$K_{CH_3CN} = 0.0072 \pm 0.0004\ M^{-1}$$

These values take into account the non-specific interaction of TBA^+ and the anion and thus do not represent the actual binding affinities of the anion within **2**. The contribution of each phenomenon individually (internal binding and ion pairing) could not be quantified as the system was underdetermined.

5.3) Equations and mathematical model

Equation for [HG], [HS] and [H]

Cage **2** was found to undergo 1:1 binding with two types of guests: anions such as Br^-, Cl^-, BF_4^-, and ClO_4^- and acetonitrile.

Each of these binding events can be expressed by the following equilibrium constants:

$$H + G \rightleftharpoons HG \qquad\qquad H + S \rightleftharpoons HS$$

$$K_{HG} = \frac{[HG]}{[H][G]}$$ (Equation 7)

$$K_{HS} = \frac{[HS]}{[H][S]}$$ (Equation 8)

where [*H*], [*G*], [*S*] are the concentrations of the unbound species and [*HG*] and [*HS*] represent the bound species. Given that the solvent concentration (~20,000 mM) is orders of magnitude greater than that of either the host or guest (~1 mM), the change in concentration upon encapsulation of solvent is very small, so that [S_{free}] ≈ [S_{total}].
From mass balance:

$$[G_t] = [G] + [HG]$$ (Equation 9)

$$[H_t] = [H] + [HG] + [HS] = [H] + [HG] + K_{HS}[H][S_t] = (1 + K_{HS}[S_t])[H] + [HG]$$ (Equation 10)

The free host concentration *H* is thus:

$$[H]=\frac{[H_t]-[HG]}{\left(1+K_{HS}[S_t]\right)}$$

(Equation 11)

Substitution into the equilibrium constant expressions gives:

$$K_{HG}=\frac{[HG]}{[H][G]}=\frac{\left(1+K_{HS}[S_t]\right)[HG]}{\left([H_t]-[HG]\right)\left([G_t]-[HG]\right)}$$

(Equation 12)

Rearranging terms, followed by division with K_{HG} gives the following quadratic equation:

$$[HG]^2-\left([H_t]+[G_t]+\frac{\left(1+K_{HS}[S_t]\right)}{K_{HG}}\right)[HG]+[H_t][G_t]=0$$

(Equation 13)

Solving for the host-guest complex [*HG*] using the quadratic formula gives the following hyperbola:

$$[HG]=\frac{\left([H_t]+[G_t]+\frac{\left(1+K_{HS}[S_t]\right)}{K_{HG}}\right)-\sqrt{\left([H_t]+[G_t]+\frac{\left(1+K_{HS}[S_t]\right)}{K_{HG}}\right)^2-4[H_t][G_t]}}{2}$$

(Equation 1)

The root with the negative radical rather than the positive radical was used in order to satisfy boundary conditions, namely that at $[G_t]$ = 0, [*HG*] must equal zero.

Likewise, an analytical function can be written for free host [*H*] as a function of $[G_t]$. According to equation 11, *H* can be expressed in terms of *HG.* Substituting the host-guest complex concentration defined by equation 1 gives:

$$[H]=\frac{[H_t]-\left(\frac{1}{2}\left([H_t]+[G_t]+\frac{\left(1+K_{HS}[S_t]\right)}{K_{HG}}-\sqrt{\left([H_t]+[G_t]+\frac{\left(1+K_{HS}[S_t]\right)}{K_{HG}}\right)^2-4[H_t][G_t]}\right)\right)}{\left(1+K_{HS}[S_t]\right)}$$

(Equation 2)

Likewise, an analytical function can be written for [*HS*] as a function of $[G_t]$. Equation 10 can be rewritten to give bound host-solvent complex as:

$$[HS]=\left([H_t]-[HG]\right)\left(\frac{K_{HS}[S_t]}{1+K_{HS}[S_t]}\right)$$

(Equation 14)

Substituting the definition of [*HG*] in equation 1 gives:

$$[HS]=\frac{K_{HS}[S_t]}{1+K_{HS}[S_t]}\left([H_t]-\frac{\left([H_t]+[G_t]+\frac{\left(1+K_{HS}[S_t]\right)}{K_{HG}}\right)-\sqrt{\left([H_t]+[G_t]+\frac{\left(1+K_{HS}[S_t]\right)}{K_{HG}}\right)^2-4[H_t][G_t]}}{2}\right)$$

(Equation 3)

Equation 1, 2 and 3 were used for the case of TBABr and TBAI.

A scaling factor C was added to Equation 1, 2 and 3 to account for the contribution of TBA^+. Furthermore, giving that [*HS*] did not change required elimination of the terms related to the otherwise competitive binding of solvent, namely 1 + K_{HS} [*S*] term with concomitant addition of the term –[*HS*] to keep the proper mass balance. Finally, although [*HS*] is essentially constant throughout the titration with larger guests, small variations were modelled as a straight line, giving Equation 4, 5 and 6 described in Section 5.2.

The Merit Function χ^2

For Gaussian distribution of measurements with individual standard deviations σ_i, the probability that the model parameters fit the N data points is

$$P \propto \prod_{i=1}^{N} e^{-\left(y_i^{obs} - y_i^{calc}\right)^2 / 2\sigma_i^2}$$ (Equation 15)

where y_i^{obs} is the range of the *ith* data point and y_i^{calc} is the range calculated for the corresponding *ith* data point according to equations 1 – 6. Taking the log of the probability allows one to work with sums rather than products, which are computationally more readily handled than exponents:

$$\ln(P) \propto -\sum_{i=1}^{N} \frac{\left(y_i^{obs} - y_i^{calc}\right)^2}{\sigma_i^2}$$ (Equation 16)

The quantity to be maximized, ln(*P*), is equivalent to minimizing –ln(*P*)

$$-\ln(P) \propto \sum_{i=1}^{N} \frac{\left(y_i^{obs} - y_i^{calc}\right)^2}{\sigma_i^2}$$ (Equation 17)

Thus, a "merit" function, or fitting function, χ^2 was defined, which is the function to be minimized during the best fit determination of the desired parameters.

$$\chi^2 = \sum_{i=1}^{N} \frac{\left(y_i^{obs} - y_i^{calc}\right)^2}{\sigma_i^2}$$ (Equation 18)

Some data points were more reliable than others and therefore each data point's contribution to the fit was weighted by $1/\sigma_i^2$, where σ_i^2 is the variance. The term y_i^{calc} is a function of the variable [*Gt*] and the binding constants K_{HG} and K_{HS}, which are the parameters to be fitted during the minimization process. Because all three curves are dependent on the parameters K_{HG} and K_{HS}, the best fit is obtained by fitting all three functions simultaneously rather than individually. Thus, we define the merit function:

$$\chi^2 = \chi^2_{HG} + \chi^2_{H} + \chi^2_{HS}$$ (Equation 19)

A Mathematica program was written to implement this. Each data points is to be fitted to only one of the equations 1 – 6, that is to only one of the χ^2 terms in equation 18. To sort the data to the appropriate term, each data point was assigned an index (1, 2, or 3), corresponding to equations 1 – 3 or 4 – 6. Thus, the N data points to be fitted were represented as an N by 3 matrix, where each row consisted of [$index_i$, x_i, y_i]. Then the Kronecker delta was used to associate individual data points to the appropriate fitting function:

$$\chi^2 = \delta_{ij}\chi^2_{HG} + \delta_{ij}\chi^2_{H} + \delta_{ij}\chi^2_{HS}$$ (Equation 20)

where $\delta_{ij} = 1$ when $i = j$ and $\delta_{ij} = 0$ when $i \neq j$.

Minimization Routine and Error Propagation

The binding constants were calculated by minimizing merit function (Equation 20) with respect to the fitted parameters, K_{HG} and K_{HS}:

$$\frac{\partial \chi^2}{\partial K_{HG}} = 0$$ (Equation 21a)

$$\frac{\partial \chi^2}{\partial K_{HS}} = 0$$

(Equation 22b)

The roots to equations 2 or 5 were found numerically using the Newton-Raphson method as implemented Mathematica's built-in NonlinearModelFit function. In this iterative method a new guess at the root x_{n+1} is derived from an initial guess (x_n), the value of the function at x_n and its derivative:

$$x_{n+1} = x_n - \frac{f(x_n)}{f'(x_n)}$$

(Equation 23)

In some cases, better convergence was achieved with the Levenberg-Marquardt minimization procedure. The standard deviations ("errors") in the fitted parameters were determined by the usual propagation of errors:

$$dK_{HG} = \sqrt{\left(\frac{\partial K_{HG}}{\partial HG}\right)_{HS}^2 dHG^2}$$

(Equation 24)

$$dK_{HS} = \sqrt{\left(\frac{\partial K_{HS}}{\partial HS}\right)_{HG}^2 dHS^2}$$

(Equation 25)

where *dHG* and *dHS* is the uncertainty of [HG] and [HS], respectively, as estimated from their residuals.

6) Characterization of libraries of heteroleptic cages

6.1) $\mathbf{Lib_{NTf_2}}$

Tris(4-aminophenyl)amine **A** (4.5 mg, 15.6 µmol, 1.0 equiv.), pararosaniline **B** (4.75 mg, 15.6 µmol, 1.0 equiv.), 2-formylpyridine (10.0 mg, 93.3 µmol, 6.0 equiv.) and $Co(NTf_2)_2$ (21.5 mg, 31.1 µmol, 2.0 equiv.) were combined in CH_3CN or CD_3CN (2.5 mL) in a sealed 5 mL reaction tube. The solution was stirred and heated at 70 °C for 18 h. A dark red stock solution of $\mathbf{Lib_{NTf_2}}$ (3.11 mM) was obtained and was used without further purification.

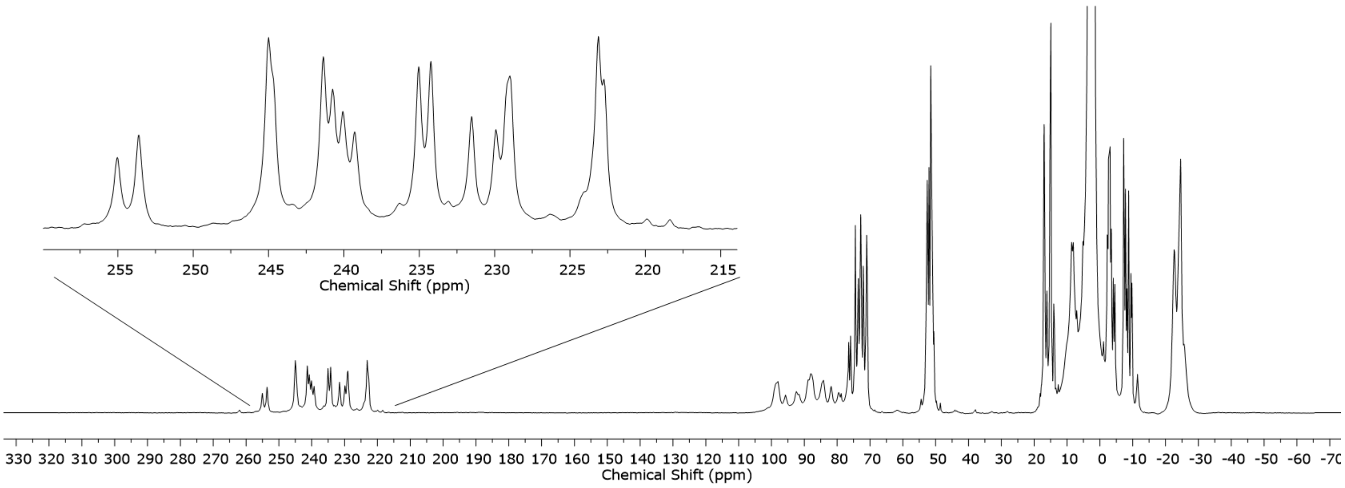

Figure S33. Wide sweep ^1H NMR spectrum (400 MHz, 298 K, CD_3CN) of $\mathbf{Lib_{NTf_2}}$

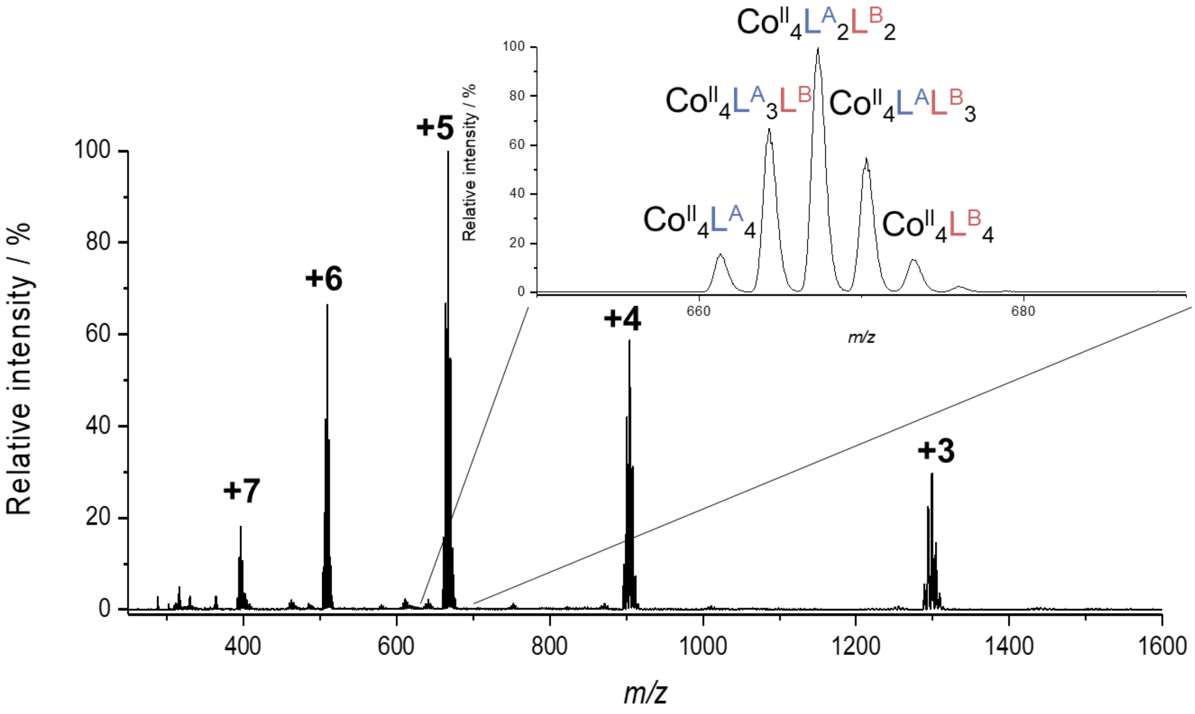

Figure S34. LR ESI-MS mass spectrum of **Lib$_{NTf_2}$**. Inset showing the z = +5 charged peaks.

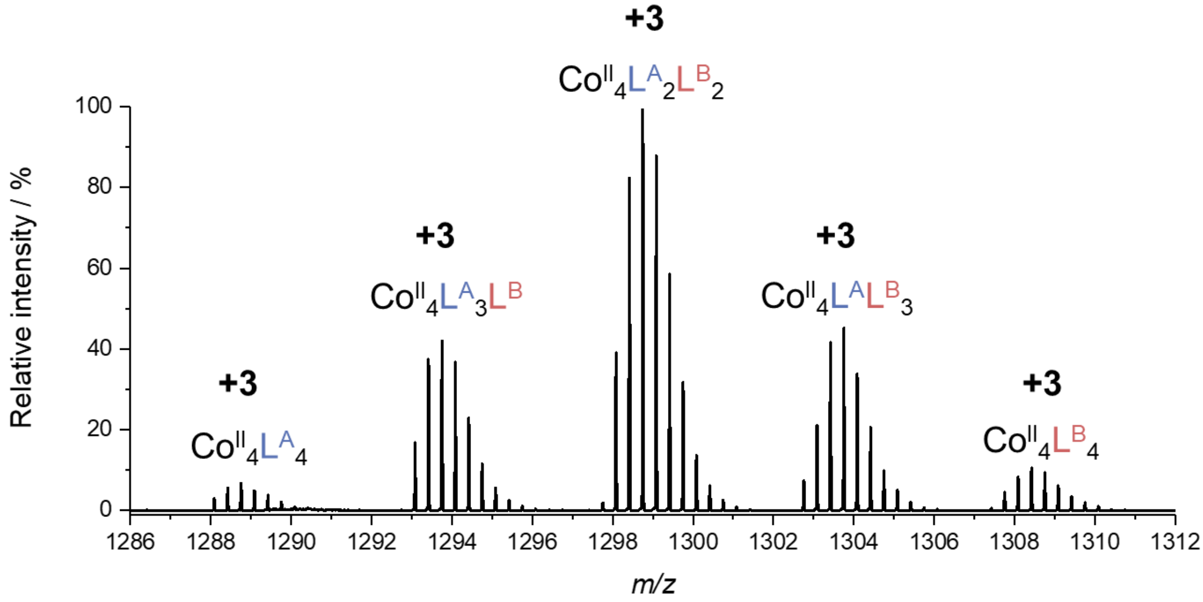

Figure S35: HR ESI-MS mass spectrum of the **Lib$_{NTf_2}$** showing the observed z = +3 charge.

6.2) Lib'$_{NTf_2}$

Tris(4-aminophenyl)amine **A** (4.5 mg, 15.6 µmol, 1.0 equiv.), pararosaniline **B** (4.75 mg, 15.6 µmol, 1.0 equiv.), 2-formylpyridine (5.0 mg, 46.6 µmol, 3.0 equiv.) and $Co(NTf_2)_2$ (21.5 mg, 31.1 µmol, 2.0 equiv.) were combined in CD_3CN (2.5 mL) in a sealed 5 mL reaction tube. The solution was stirred and heated at 70 °C for 18 h. A dark red stock solution of the library of cages (3.11 mM) was obtained and was used without further purification.

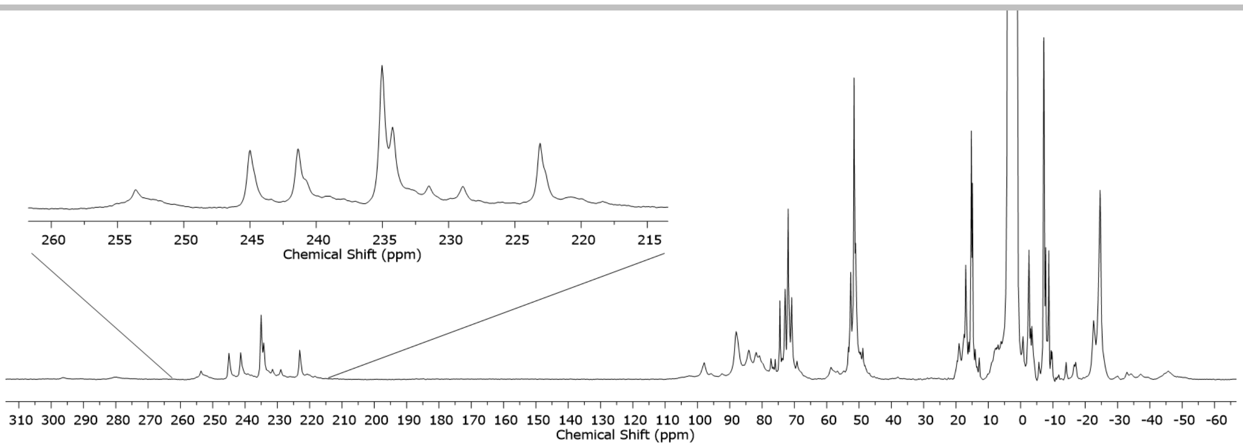

Figure S36. Wide sweep ^{1}H NMR spectrum (400 MHz, 298 K, CD_3CN) of the off-stoichiometry library of cages **Lib'**$_{\mathbf{NTf_2}}$.

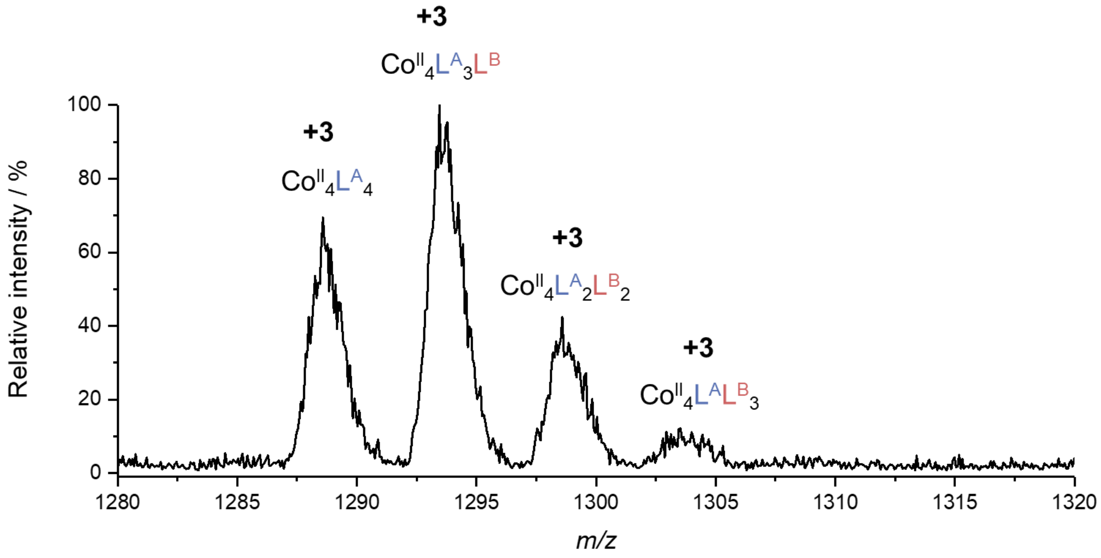

Figure S37. LR ESI-MS mass spectrum of the off-stoichiometry library of cages **Lib'**$_{\mathbf{NTf_2}}$ showing the observed z = +3 charge. The predominant species are those incorporating high numbers of **L$^{\mathbf{A}}$**.

ESI-MS revealed the presence of congeners of stoichiometry Co$^{II}_4$**L$^{\mathbf{A}}$**$_x$**L$^{\mathbf{B}}$**$_{(4-x)}$ with $2 \leq x \leq 4$ predominantly. Three sets of signals containing six, four, and one resonance each were observed in the imine region of the ^{1}H NMR which were attributed to Co$^{II}_4$**L$^{\mathbf{A}}$**$_4$ (*T* symmetry, one signal per imine proton), Co$^{II}_4$**L$^{\mathbf{A}}$L$^{\mathbf{B}}$**$_3$ (C_3 symmetric, four signals) and Co$^{II}_4$**L$^{\mathbf{A}}$**$_2$**L$^{\mathbf{B}}$**$_2$ (S_4 symmetric, six signals).[15]

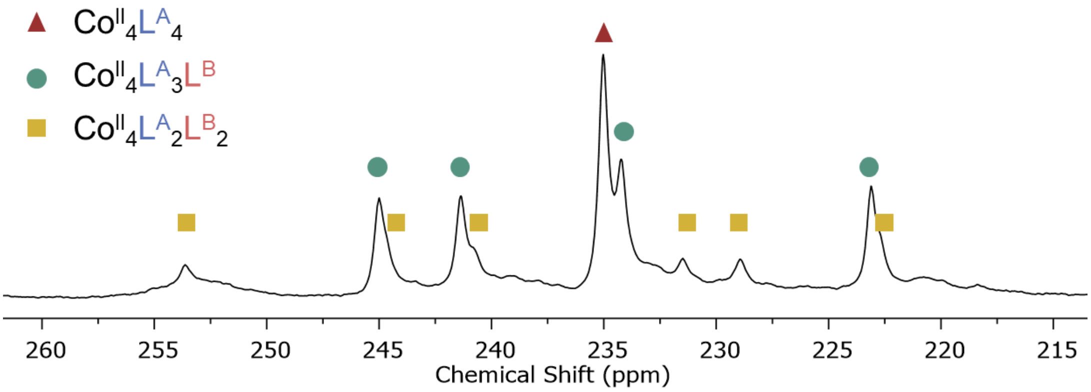

Figure S38. Wide sweep ^{1}H NMR spectrum (400 MHz, 298 K, CD_3CN) zoomed in the 260-215 ppm region of the off- stoichiometry library of cages **Lib'**$_{\mathbf{NTf_2}}$. Assignment of the resonance corresponding to each species with the signals corresponding to Co$^{II}_4$**L$^{\mathbf{A}}$**$_4$ marked by a red triangle, Co$^{II}_4$**L$^{\mathbf{A}}$L$^{\mathbf{B}}$**$_3$ by a green circle and Co$^{II}_4$**L$^{\mathbf{A}}$**$_2$**L$^{\mathbf{B}}$**$_2$ by a yellow square.

Based on this assignment, each signal observed in the imine region of the ^{1}H NMR spectrum of **Lib$_{NTf_2}$** was attributed to one of the congeners present.

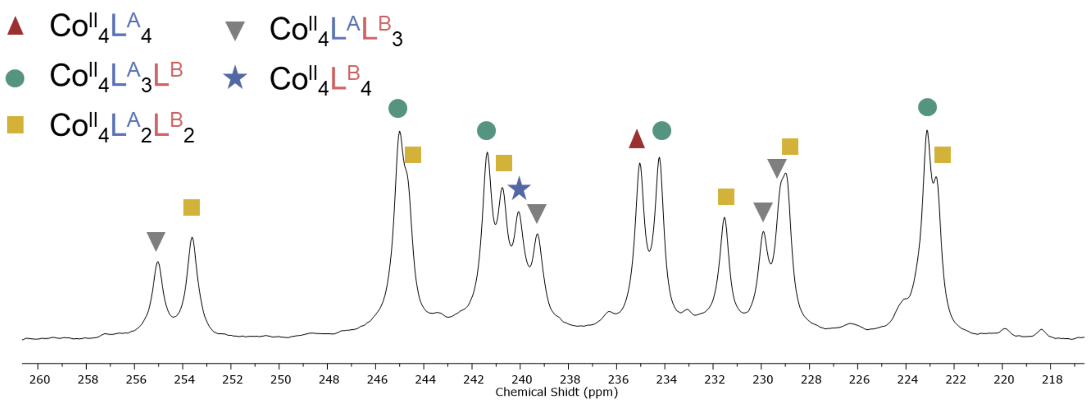

Figure S39. Wide sweep ^{1}H NMR spectrum (400 MHz, 298 K, CD$_3$CN) zoomed in the 260-215 ppm region of **Lib$_{NTf_2}$**. Assignment of the resonance corresponding to each species with the signals corresponding to Co$^{II}_4$**L$^A_4$** marked by a red triangle, Co$^{II}_4$**L^{A}L$^B_3$** by a green circle, Co$^{II}_4$**L$^A_2$L$^B_2$** by a yellow triangle, Co$^{II}_4$**L^{A}L$^B_3$** by a grey inverted triangle and Co$^{II}_4$**L$^B_4$** by a blue star.

6.3) Kinetic study of the formation of Lib$_{NTf_2}$

The kinetics of the formation of the library of cages in the absence of templating anions (**Lib$_{NTf_2}$**) was studied by NMR.

Tris(4-aminophenyl)amine **A** (1.0 mg, 3.5 µmol, 1.0 equiv.), pararosaniline **B** (1.05 mg, 3.5 µmol, 1.0 equiv.), 2-formylpyridine (2.2 mg, 20.7 µmol, 6.0 equiv.) and Co(NTf$_2$)$_2$ (4.7 mg, 7.0 µmol, 2.0 equiv.) were combined in CD$_3$CN (0.5 mL) in a sealed J-Young NMR tube. The solution was first sonicated at room temperature for 30 min, then heated at 50 °C for 30 min, 60 min, 120 min, 180 min and 18 h. The sample tube was then heated at 70 °C for 18 h, 44 h and 72 h. Each time, a ^{1}H NMR spectrum was acquired.

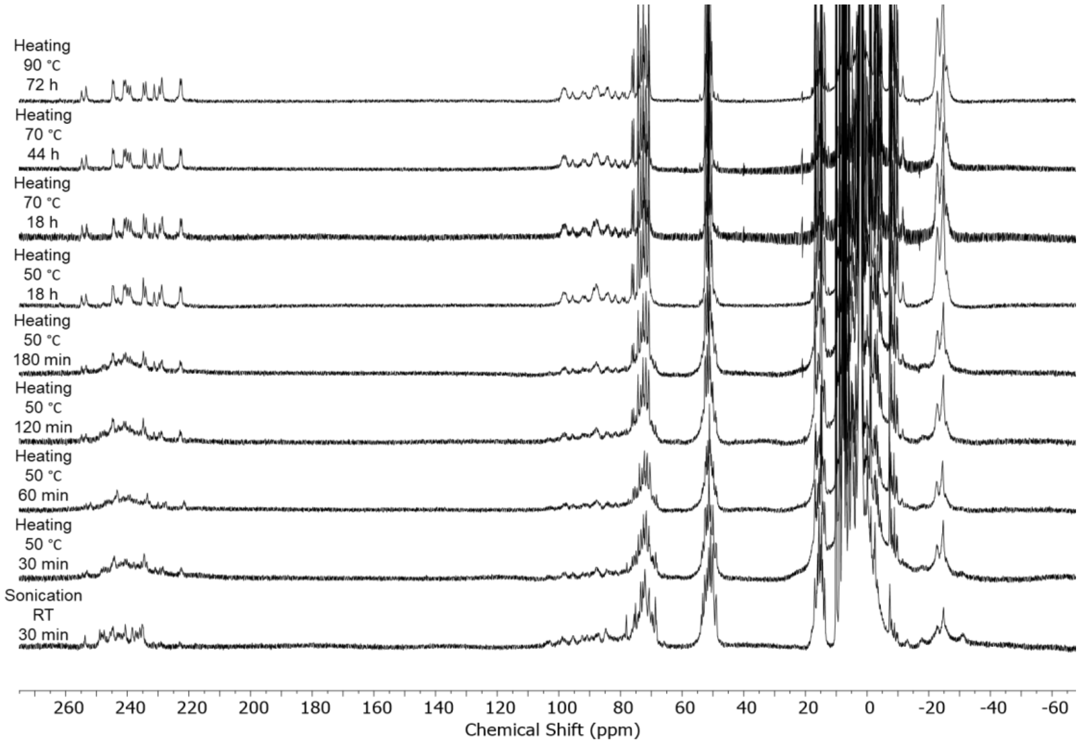

Figure S40. Wide sweep ^{1}H NMR spectra (400 MHz, 298 K, CD$_3$CN) of **Lib$_{NTf_2}$** after sonication at room temperature for 30 min, heating at 50 °C for 30 min, 60 min, 120 min, 180 min and 18 h and heating at 70 °C for 18 h, 44 h and 72 h (bottom to top).

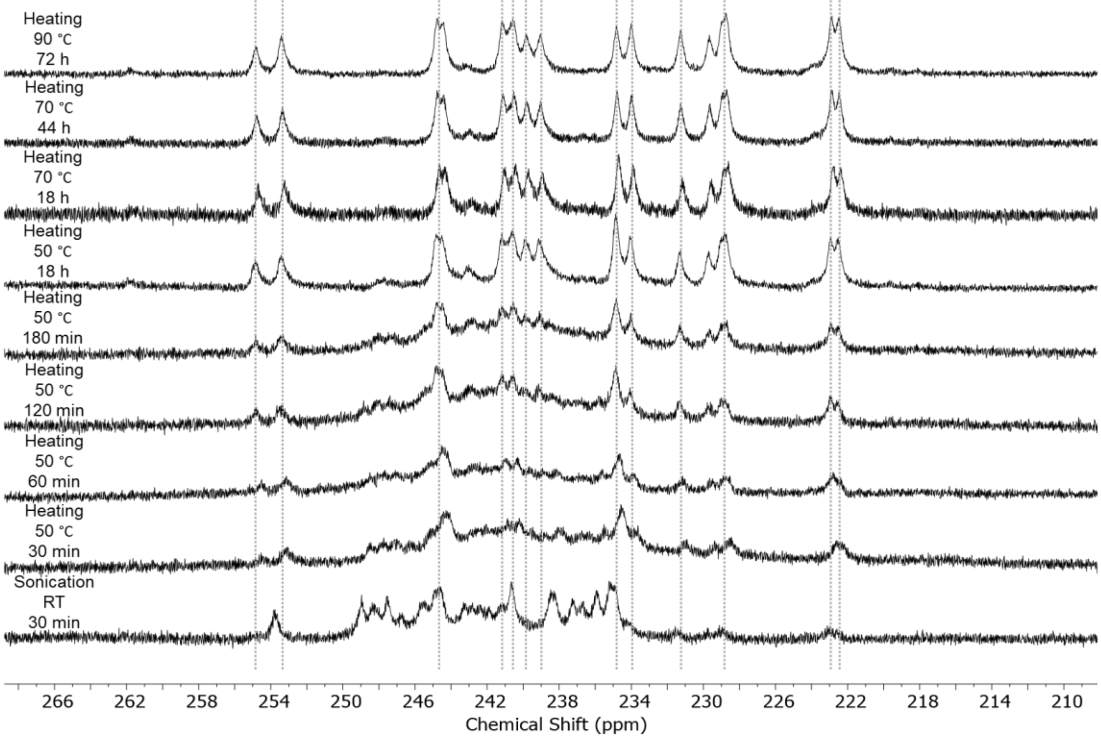

Figure S41. Zoom into the 210 – 266 ppm region of the wide sweep ^{1}H NMR spectra (400 MHz, 298 K, CD_3CN) of $\mathbf{Lib_{NTf_2}}$. The dotted line highlights the position of the signal for the members of the library in its final state.

Intermediate species were observed at first, which could not be assigned to any of the members of the final library. Broad, complex signals, which are consistent with the presence of a dynamic combinatorial library (DCL) made of complex, low symmetry species were also observed below 18 h. After heating at 50 °C for 2 h, signals corresponding to the final members of the library were visible. Heating at 70 °C for 18 h resulted in conversion of most of the DCL into discrete species, with no apparent changes upon heating for longer periods.

6.4) $\mathbf{Lib_{ClO_4}}$ and $\mathbf{Lib_{BF_4}}$

Tris(4-aminophenyl)amine **A** (4.5 mg, 15.60 µmol, 1.0 equiv.), Pararosaniline **B** (4.75 mg, 15.60 µmol, 1.0 equiv.), 2-formylpyridine (10.0 mg, 93.30 µmol, 6.0 equiv.), $Co(NTf_2)_2$ (21.5 mg, 31.10 µmol, 2.0 equiv.) and $TBAClO_4$ or $TBABF_4$ (7.80 µmol, 0.5 equiv.) were combined in CH_3CN or CD_3CN (2.5 mL) in a sealed 5 mL reaction tube. The solutions were stirred and heated at 70 °C for 72 h. Bark red stock solution of $\mathbf{Lib_{ClO_4}}$ or $\mathbf{Lib_{BF_4}}$ (3.11 mM) were obtained and were used without further purification.

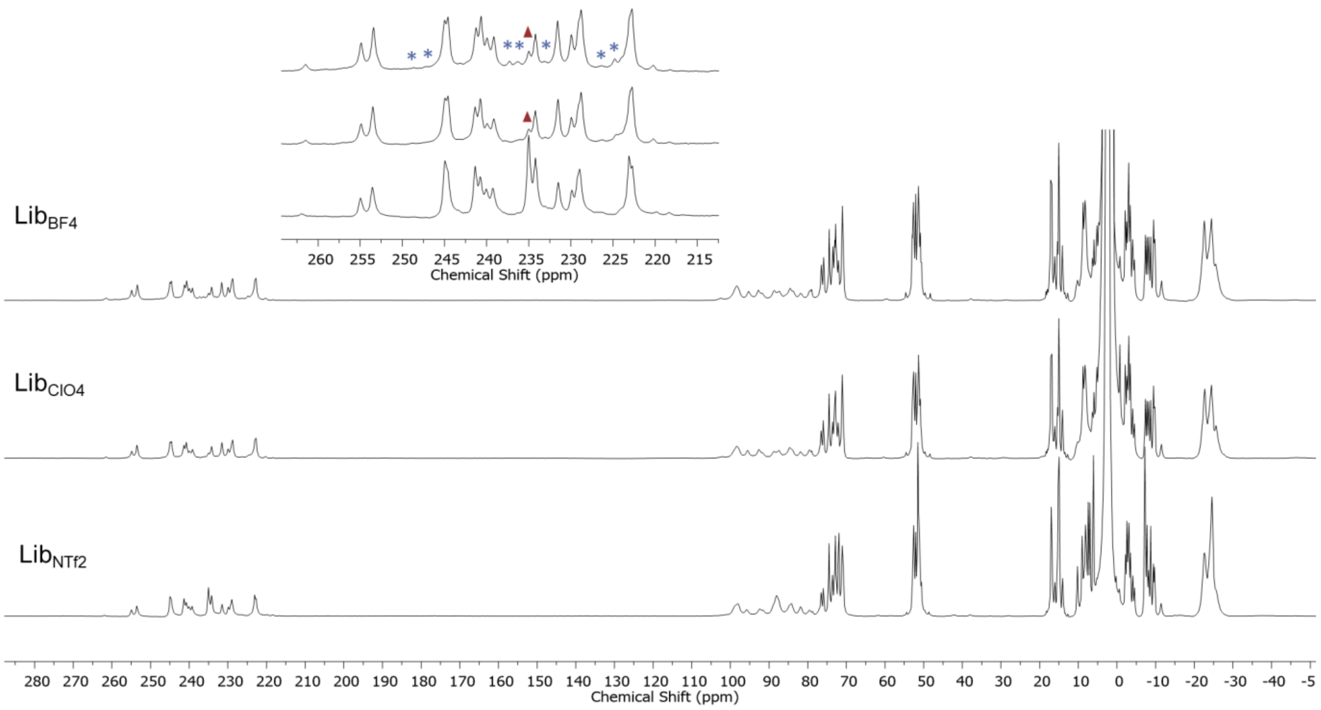

Figure S42. Wide sweep ^{1}H NMR spectrum (400 MHz, 298 K, CD_3CN) of $\mathbf{Lib_{NTf_2}}$ (bottom) and $\mathbf{Lib_{ClO_4}}$ (middle) and $\mathbf{Lib_{BF_4}}$ (top). The new peaks corresponding to the encapsulated guests within the library are marked by blue stars. The peak corresponding to **1** is marked by a red triangle.

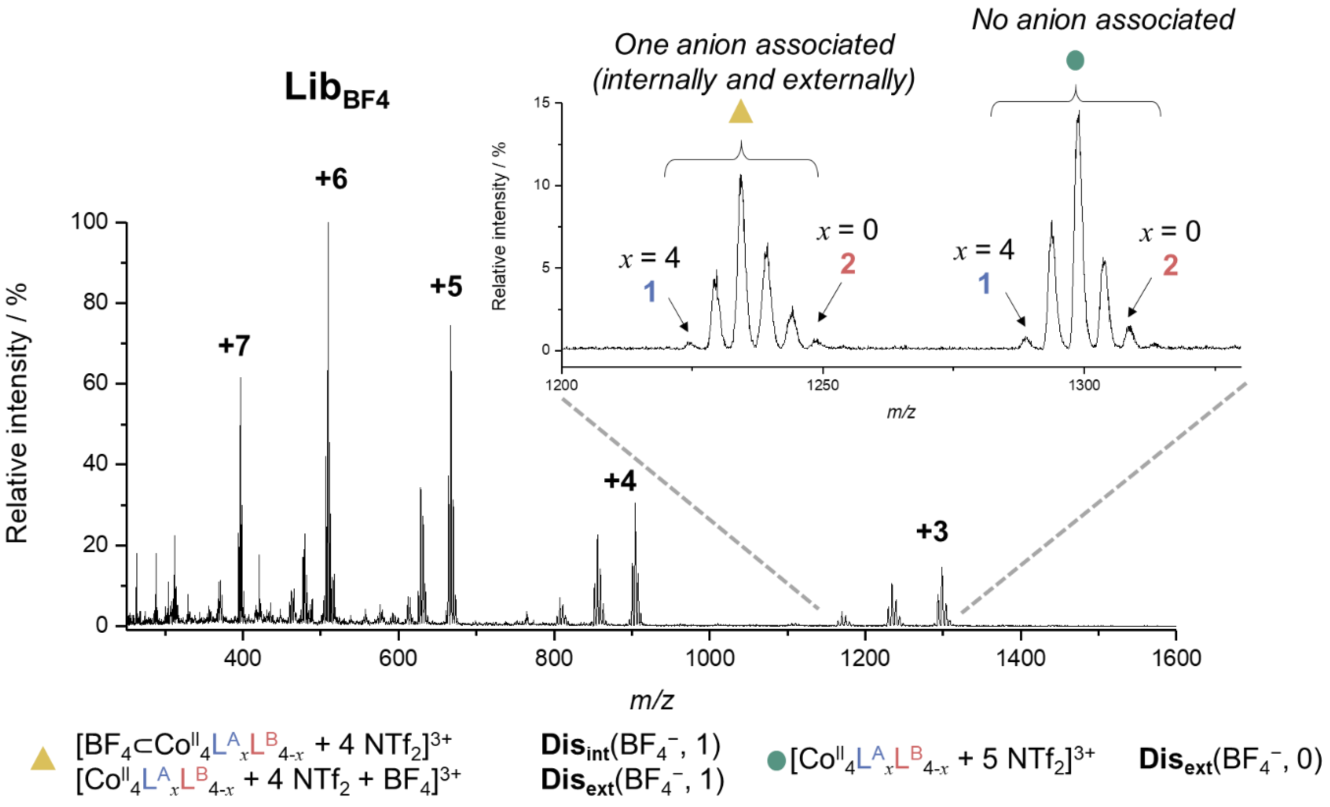

Figure S43. LR-ESI-MS of $\mathbf{Lib_{BF_4}}$ obtained after addition of $TBABF_4$ (2 equiv.) to $\mathbf{Lib_{NTf_2}}$. Zoom in the +3 region of the mass spectra showing the clusters corresponding to cages with no BF_4^- associated (green circle) and cages with one BF_4^- associated either internally or externally (yellow triangle).

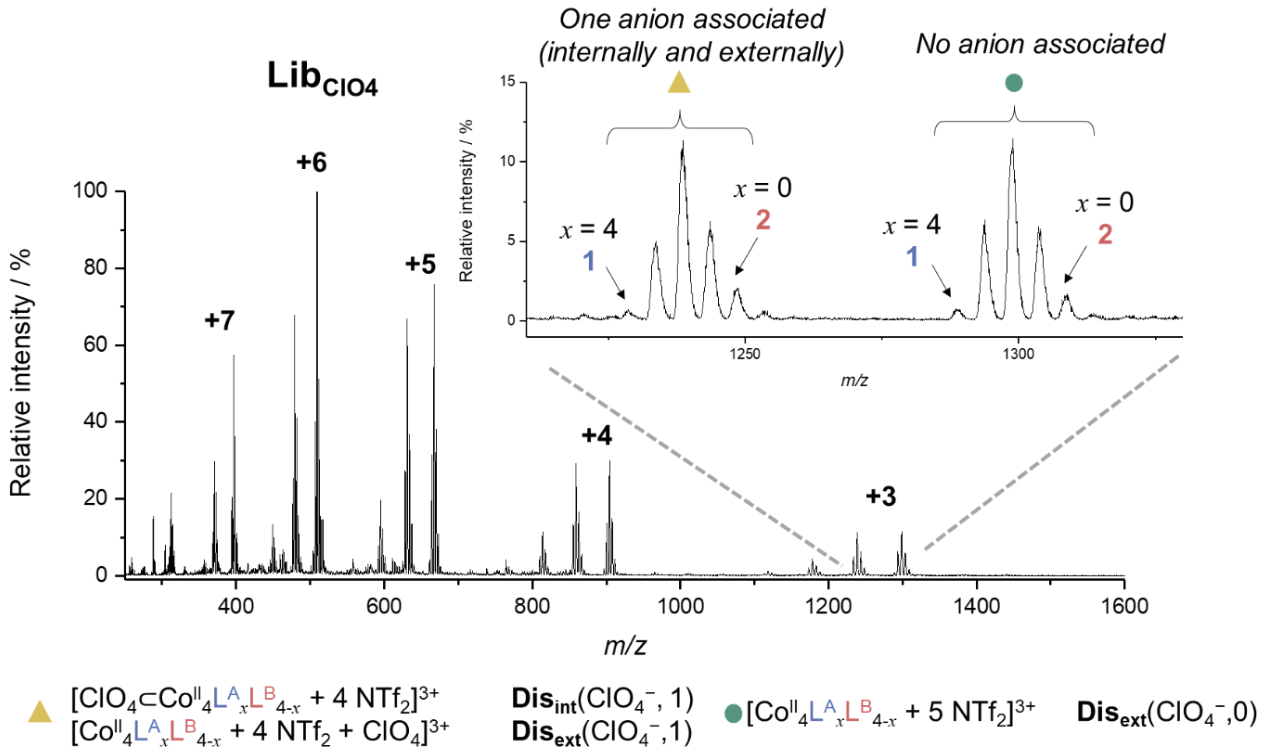

Figure S44. LR-ESI-MS of **Lib$_{ClO_4}$** obtained after addition of TBAClO$_4$ (2 equiv.) to **Lib$_{NTf_2}$**. Zoom in the +3 region of the mass spectra showing the clusters corresponding to cages with no ClO$_4^-$ associated (green circle) and cages with one ClO$_4^-$ associated either internally or externally (yellow triangle).

6.5) Lib$_I$ and Lib$_{Br}$.

TBAI or TBABr (2 equiv.) were added to a solution of **Lib$_{NTf_2}$** (3.11 mM). The solutions were stirred and heated at 70 °C for 72 h. Dark red solution of the **Lib$_I$** or **Lib$_{Br}$** (3.11 mM) were obtained and was used without further purification.

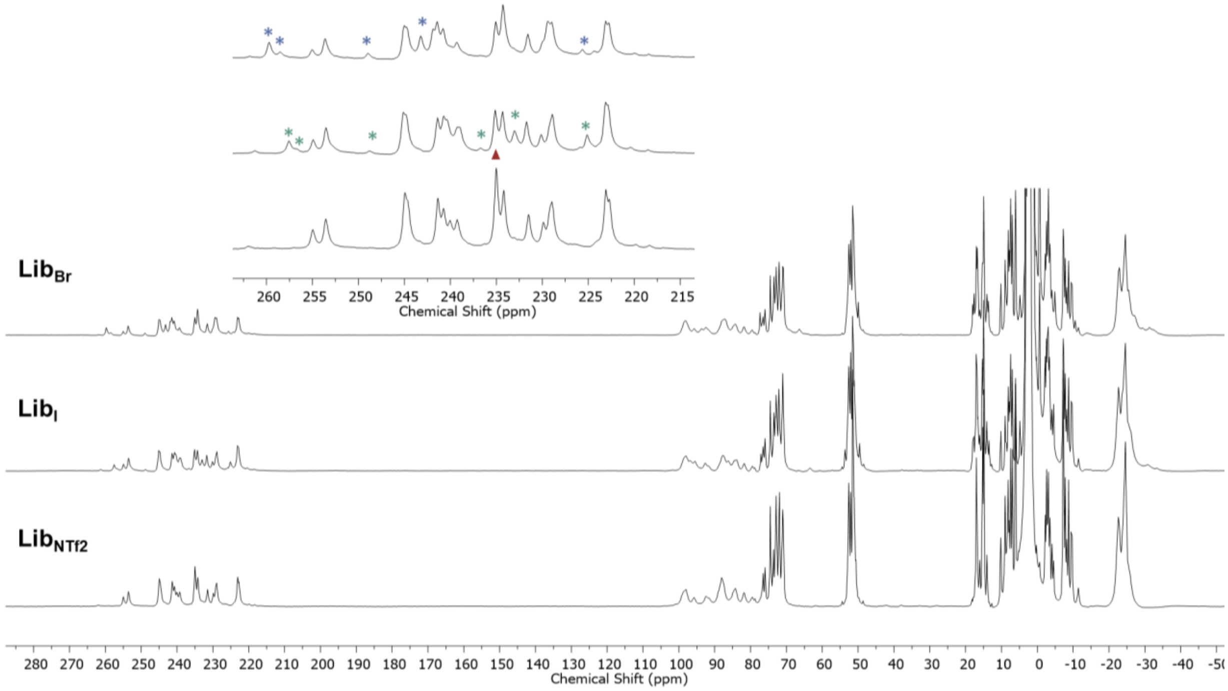

Figure S45. Wide sweep ^{1}H NMR spectrum (400 MHz, 298 K, CD$_3$CN) of **Lib$_{NTf_2}$** (bottom) and **Lib$_I$** (middle) and **Lib$_{Br}$** (top). The new peaks corresponding to the encapsulated guests within the library are marked by blue stars. The peak corresponding to **1** is marked by a red triangle.

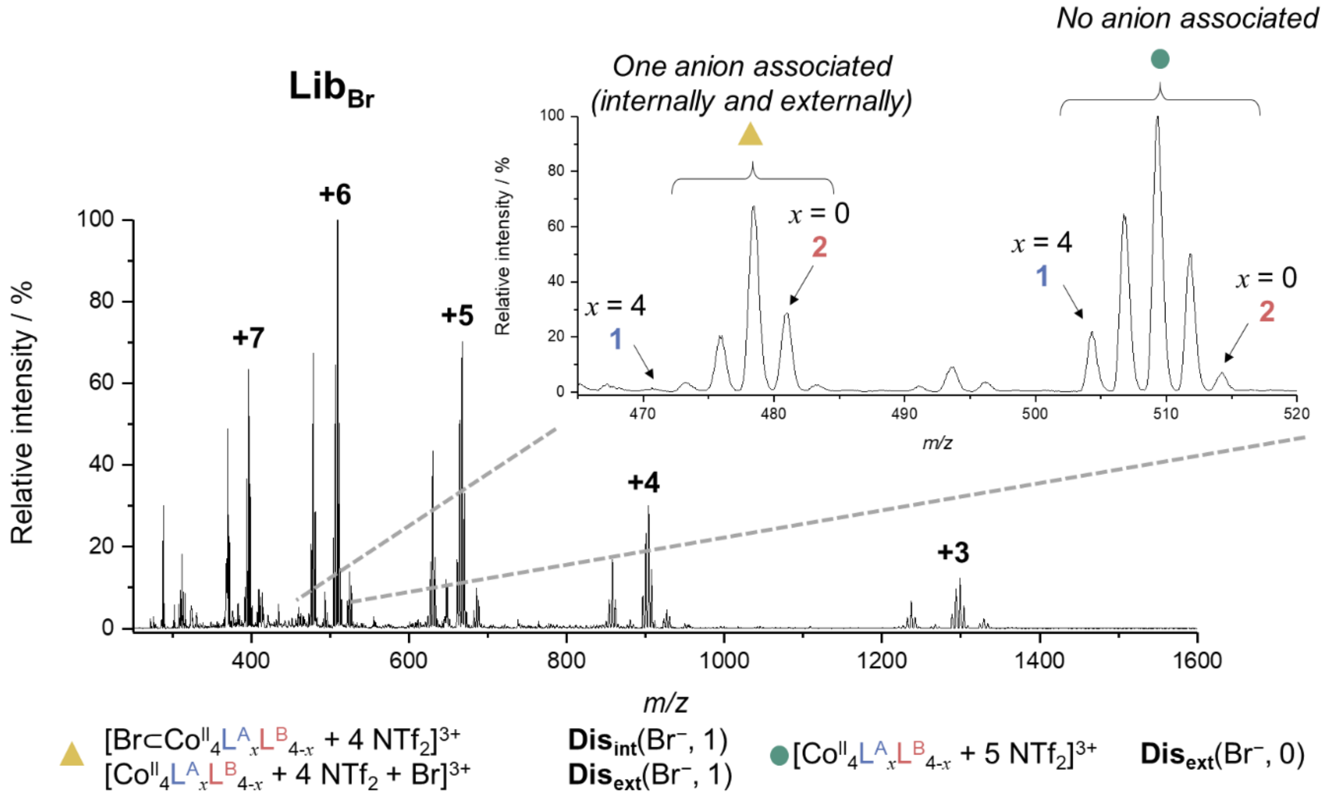

Figure S46. LR-ESI-MS of **Lib$_{Br}$** obtained after addition of TBABr (2 equiv.) to **Lib$_{NTf_2}$**. Zoom in the +6 region of the mass spectra showing the clusters corresponding to cages with no Br⁻ associated (green circle) and cages with one Br⁻ associated either internally or externally (yellow triangle).

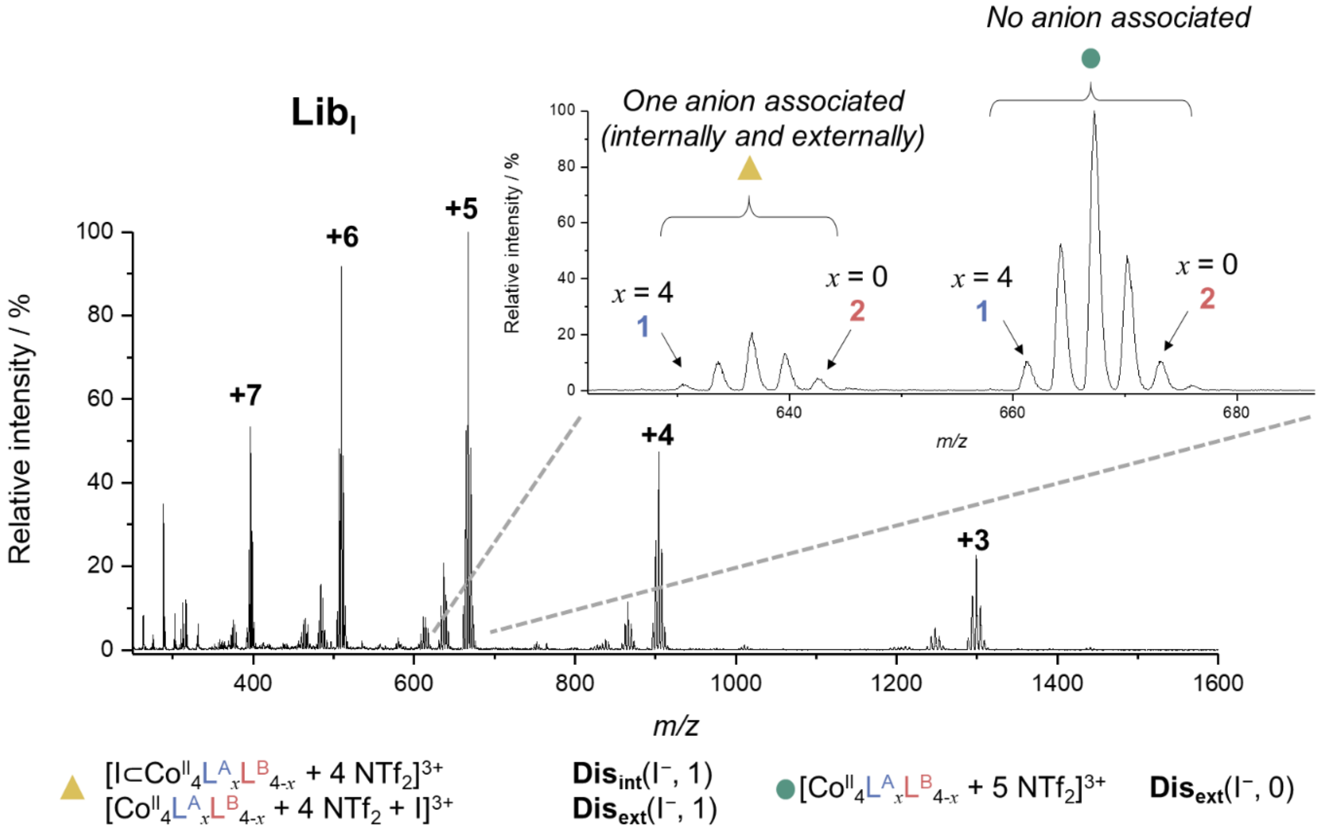

Figure S47. LR-ESI-MS of **Lib$_I$** obtained after addition of TBAI (2 equiv.) to **Lib$_{NTf_2}$**. Zoom in the +5 region of the mass spectra showing the clusters corresponding to cages with no I⁻ associated (green circle) and cages with one I⁻ associated either internally or externally (yellow triangle).

7) Distributions and energies calculations

7.1) Response factor of 1 and 2

An equimolar mixture of **1** and **2** (1 mM each) was freshly prepared and quickly analysed by ESI-MS before scrambling of the ligands could occur. The relative integrals of the peaks for **1** (0.49 ± 0.042) and **2** (0.51 ± 0.042) did not deviate significantly from the expected 1:1 ratio when averaged across the observable charge states of +3 to +7. It was therefore hypothesised that the heteroleptic congeners of the library have similar response factors and that the integrals of the signals observed for each species are proportional to the species concentration in solution.

Table S3. Normalised integrals of **1** and **2** for each charge state peaks. Average and standard deviation across charge states.

**Charge state**	**7**	**6**	**5**	**4**	**3**	**average**	**SD**
1	0.45	0.51	0.44	0.50	0.55	0.49	0.042
2	0.55	0.49	0.56	0.50	0.45	0.51	0.042

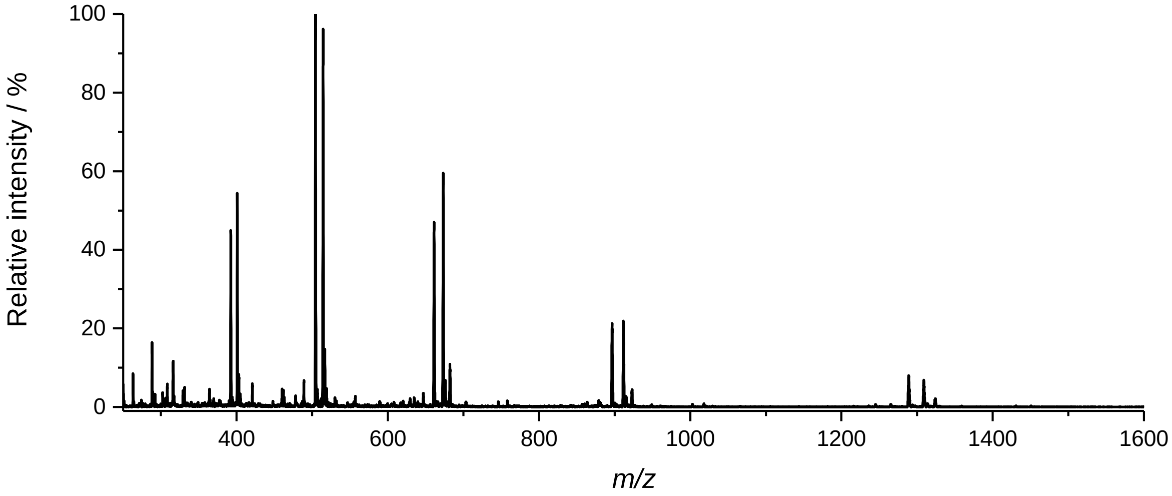

Figure S48. LR-ESI-MS mass spectrum of a freshly combined equimolar mixture of **1** and **2** showing similar intensities for **1** and **2** within each charge state.

7.2) $\mathbf{Lib_{NTf_2}}$

<u>*By ESI-MS*</u>

In the case of $\mathbf{Lib_{NTf_2}}$ (with NTf_2 in presence only), within each charge state observed in the mass spectra, the integrals of the *m/z* peaks were normalised and the values obtained were averaged across the +7 to +3 charge states. Experiments were repeated three times to minimise the error. The distributions obtained were compared to the statistical distribution obtained by normalizing to 1 the binomial distribution (Pascal's triangle with n = 4).

Each structure type was identified by the number of ligand $\mathbf{L^A}$ they possess. The relative energy of each could be determined by using the following formula (Blotzman equation):[16]

$$\Delta E_{rN} = -RTln(K_N), \qquad with\ K_N = \frac{\left(\frac{I_N}{I_{statN}}\right)}{I_0}$$

where I_N represented the normalized integrals of the MS peak for the structure incorporating N $\mathbf{L^A}$, I_{statN} was the corresponding normalized binomial coefficient (expected normalized integral for the structure incorporating N $\mathbf{L^A}$), and I_0 was the normalized integral

of the MS peak for the original structure, here **2** (N = 0). Once averaged across the 3 repeats of the experiment, the following values were obtained for **Lib**$_{\mathbf{NTf_2}}$:

Table S4. TOP: Normalised integrals of **Lib**$_{\mathbf{NTf_2}}$ for each charged state (averaged over three repeats of the experiment). BOTTOM: Relative energies (kJ.mol^{-1}) for the **Lib**$_{\mathbf{NTf_2}}$ for each charge state (averaged over three repeats of the experiment). Average and standard deviation across all charge states.

	Charge	7	6	5	4	3	Average	SD
**Number of L^A Integrals**	0	0.057	0.055	0.061	0.067	0.074	0.054	0.02
	1	0.228	0.245	0.263	0.277	0.29	0.208	0.015
	2	0.374	0.401	0.398	0.394	0.391	0.394	0.009
	3	0.236	0.233	0.22	0.208	0.196	0.277	0.022
	4	0.104	0.065	0.058	0.054	0.05	0.067	0.007
**Number of L^A Relative energies (kJ.mol^{-1})**	0	0.000	0.000	0.000	0.000	0.000	0.000	0.000
	1	1.400	0.279	0.140	0.078	0.070	0.393	0.509
	2	1.267	-0.057	-0.322	-0.501	-0.640	-0.051	0.687
	3	1.486	0.154	-0.300	-0.629	-0.904	-0.039	0.840
	4	1.493	0.417	-0.120	-0.550	-0.941	0.060	0.847

The relative Gibbs energies showed no significant deviation from the null hypothesis by Fisher's test as the p-value was above the significance threshold of 0.05 ($F = 0.262$ and $p = 0.660$). The distribution of congeners within **Lib**$_{\mathbf{NTf_2}}$ was thus inferred to not significantly deviate from the binomial distribution. The values obtained for the **Lib**$_{\mathbf{NTf_2}}$ were therefore used in the following calculation as the "statistical" distribution.

By NMR

The imine signals in the ^{1}H NMR spectra of **Lib**$_{\mathbf{NTf_2}}$ were each assigned to a cage species within the library. The signals were deconvoluted and integrated using MestReNova 12.0.4-22023, giving the absolute integrals (Figure S49).

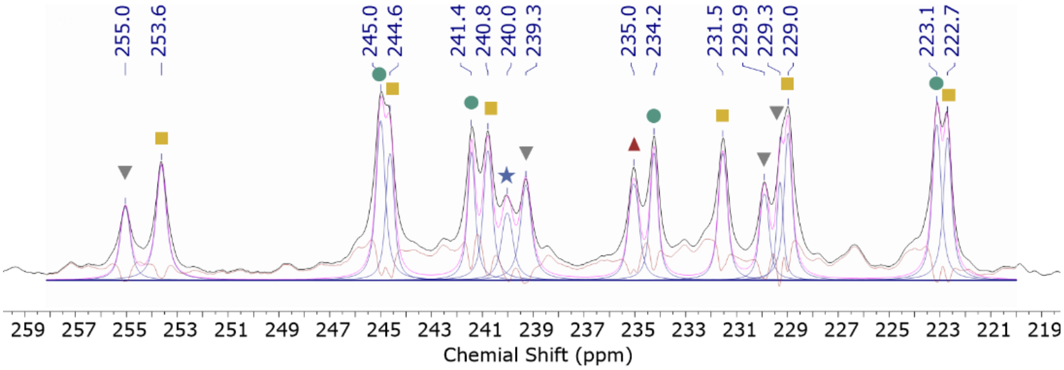

Symbol	Congener	Number of L$_A$	Absolute Integral
★	$Co^{II}_4L^B_4$	0	21435.6
▼	$Co^{II}_4L^AL^B_3$	1	90259.66
■	$Co^{II}_4L^A_2L^B_2$	2	197985.3
●	$Co^{II}_4L^A_3L^B$	3	137074.4
▲	$Co^{II}_4L^A_4$	4	27839.1

Figure S49. ^{1}H NMR spectrum (imine region, 219 – 259 ppm) of **Lib**$_{\mathbf{NTf_2}}$ showing the deconvolution applied to the signals (top) and a table giving the absolute integrals thus obtained (bottom).

These values are in close agreement to those obtained using ESI-MS (Figure S50). This observation suggests the ESI-MS spectra reflect the relative concentrations of cage species in solution.

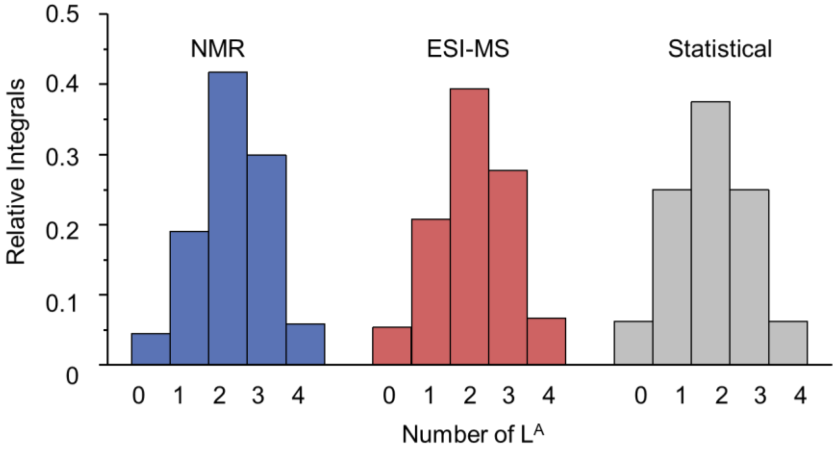

Congener	Number of L_A	Normalised Integral by NMR	Normalised Integral by ESI-MS	Statistical distribution
$Co^{II}_4L^B_4$	0	0.045	0.054	0.0625
$Co^{II}_4L^A_3L^B$	1	0.190	0.208	0.25
$Co^{II}_4L^A_2L^B_2$	2	0.417	0.394	0.375
$Co^{II}_4L^AL^B_3$	3	0.299	0.277	0.25
$Co^{II}_4L^A_4$	4	0.059	0.067	0.0625

Figure S50. Plot of the relative integrals for each member of $\mathbf{Lib}_{NTf_2}$ obtained by ESI-MS and NMR compared to the statistical distribution (top) and table giving the normalized integrals obtained in both cases (bottom).

7.3) $\mathbf{Lib}_{ClO_4}$, $\mathbf{Lib}_{BF_4}$, $\mathbf{Lib}_{I}$ and $\mathbf{Lib}_{Br}$.

In all cases, the ESI-MS spectra displayed clusters of peaks indicative of the $Co^{II}_4\mathbf{L^A}_x\mathbf{L^B}_{(4-x)}$ cages associated with zero, one or more X^- (Figures S43, S44, S46, S47). The presence of a signal for cages with no X^- associated ($Co^{II}_4\mathbf{L^A}_x\mathbf{L^B}_{4-x}$ + $mNTf_2$) along with signals for **1** (which does not bind X^-) associated with X^- indicated that X^- can either be associated externally or internally with the cages. The species within the library $\mathbf{Lib}_X$ which do not have an encapsulated X^- were defined as belonging to a distribution named $\mathbf{Dis}_{ext}(X^-, n)$ with n the number of anions X^- externally associated (0≤n<8). The species within $\mathbf{Lib}_X$ which do have an encapsulated X^- were defined as belonging to a distribution named $\mathbf{Dis}_{int}(X^-, n)$ with n the overall number of anions X^- associated (internally and externally, 1≤n<8). The new library $\mathbf{Lib}_X$ thus consists of a distribution of species with:

- no X^- associated ([$Co^{II}_4\mathbf{L^A}_x\mathbf{L^B}_{4-x}$ + $mNTf_2$], 0<m<8) which is referred to as $\mathbf{Dis}_{ext}(X^-,0)$)
- X^- externally associated ([$Co^{II}_4\mathbf{L^A}_x\mathbf{L^B}_{4-x}$ + nX^- + $(m–n)NTf_2$], 1≤n<8, n<m<8) which is referred to as $\mathbf{Dis}_{ext}(X^-,n)$
- X^- internally associated ([$X^-\subset Co^{II}_4\mathbf{L^A}_x\mathbf{L^B}_{4-x}$ + $(n–1)X^-$ + $(m–n)NTf_2$], 1≤n<8, n<m<8) which is referred to as $\mathbf{Dis}_{int}(X^-,n)$)

Information on the impact of anion X^- on the library was obtained by analyzing the distribution of congeners $X^-\subset Co^{II}_4\mathbf{L^A}_x\mathbf{L^B}_{4-x}$ of the library $\mathbf{Lib}_X$, which are represented by $\mathbf{Dis}_{int}(X^-,n)$. However, the peaks corresponding to $\mathbf{Dis}_{int}(X^-,n)$ overlap with those of $\mathbf{Dis}_{ext}(X^-,n)$ in the mass spectra as they represent complexes of the same *m/z* ratios (Figures S43, S44, S46, S47, yellow triangle). The integrals of the *m/z* peaks in the ESI-MS were calculated for each congener within each charge state observed for the cluster with zero and one anion associated (Figures S43, S44, S46, S47, yellow triangle and green circle) and are given in Table S5. Due to similarity of the response factors for **1** and **2** across charge states, the integrals of the *m/z* peaks were assumed to be proportional to the concentration of each species in solution.

Table S5. Normalized integrals for **$\mathrm{Dis_{int}}$**(X^-,1) + **$\mathrm{Dis_{ext}}$**(X^-,1) (overlapping, demarked by a yellow tringle in the ESI-MS spectra - Figures S43, S44, S46, S47) and **$\mathrm{Dis_{ext}}$**(X^-,0) (demarked by a green circle in the ESI-MS) given for each observable charge state for three repeats of the experiments. The average and the standard deviation of these integrals across all charge states and repeats are given in the last two columns.

$\mathrm{Dis_{ext}}$(ClO_4^-,1) + $\mathrm{Dis_{int}}$(ClO_4^-,1)	Repeat 1					Repeat 2				
Number of **L**^{A}	+7 charge	+6 charge	+5 charge	+4 charge	+3 charge	+7 charge	+6 charge	+5 charge	+4 charge	+3 charge
4	0.025	0.024	0.030	0.029	0.029	0.044	0.034	0.032	0.037	0.034
3	0.167	0.169	0.184	0.199	0.199	0.167	0.164	0.182	0.186	0.185
2	0.394	0.423	0.423	0.441	0.440	0.372	0.397	0.418	0.420	0.411
1	0.291	0.274	0.273	0.246	0.245	0.313	0.315	0.302	0.292	0.302
0	0.124	0.110	0.090	0.085	0.088	0.103	0.091	0.066	0.065	0.069
$\mathrm{Dis_{ext}}$(ClO_4^-,1) + $\mathrm{Dis_{int}}$(ClO_4^-,1)	Repeat 3					Combined				
Number of **L**^{A}	+7 charge	+6 charge	+5 charge	+4 charge	+3 charge	Mean	ESD			
4	0.068	0.047	0.028	0.041	0.033	0.036	0.011			
3	0.149	0.125	0.132	0.136	0.129	0.165	0.024			
2	0.311	0.317	0.354	0.352	0.362	0.389	0.041			
1	0.337	0.360	0.388	0.364	0.373	0.312	0.043			
0	0.135	0.150	0.097	0.107	0.103	0.099	0.024			

$\mathrm{Dis_{ext}}$(ClO_4^-,0)	Repeat 1					Repeat 2				
Number of **L**^{A}	+7 charge	+6 charge	+5 charge	+4 charge	+3 charge	+7 charge	+6 charge	+5 charge	+4 charge	+3 charge
4	0.025	0.033	0.026	0.034	0.031	0.084	0.058	0.036	0.038	0.037
3	0.160	0.183	0.201	0.212	0.242	0.150	0.173	0.191	0.193	0.204
2	0.435	0.465	0.463	0.451	0.437	0.358	0.412	0.435	0.427	0.430
1	0.281	0.242	0.238	0.228	0.219	0.320	0.277	0.251	0.277	0.273
0	0.099	0.077	0.072	0.076	0.070	0.087	0.079	0.087	0.066	0.056
$\mathrm{Dis_{ext}}$(ClO_4^-,0)	Repeat 3					Combined				
Number of **L**^{A}	+7 charge	+6 charge	+5 charge	+4 charge	+3 charge	Mean	ESD			
4	0.083	0.052	0.032	0.041	0.036	0.043	0.018			
3	0.099	0.124	0.134	0.136	0.148	0.170	0.037			
2	0.244	0.335	0.374	0.376	0.389	0.402	0.057			
1	0.427	0.333	0.322	0.343	0.340	0.291	0.054			
0	0.147	0.155	0.139	0.104	0.087	0.094	0.029			

$\mathrm{Dis_{ext}}$(BF_4^-,1) + $\mathrm{Dis_{int}}$(BF_4^-,1)	Repeat 1					Repeat 2				
Number of **L**^{A}	+7 charge	+6 charge	+5 charge	+4 charge	+3 charge	+7 charge	+6 charge	+5 charge	+4 charge	+3 charge
4	0.071	0.038	0.022	0.026	0.022	0.139	0.032	0.009	0.025	0.021
3	0.126	0.079	0.098	0.179	0.174	0.094	0.069	0.100	0.131	0.128
2	0.289	0.288	0.394	0.404	0.419	0.234	0.277	0.344	0.342	0.355

1	0.311	0.387	0.343	0.284	0.276	0.316	0.409	0.378	0.347	0.349
0	0.203	0.208	0.143	0.107	0.108	0.216	0.212	0.168	0.155	0.147

$Dis_{ext}(BF_4^-,1)$ + $Dis_{int}(BF_4^-,1)$	Repeat 3					Combined	
Number of **L**$^{\mathbf{A}}$	+7 charge	+6 charge	+5 charge	+4 charge	+3 charge	Mean	ESD
4	0.141	0.090	0.028	0.040	0.032	0.049	0.041
3	0.090	0.098	0.097	0.129	0.118	0.114	0.030
2	0.202	0.299	0.380	0.388	0.411	0.335	0.065
1	0.282	0.367	0.341	0.324	0.335	0.337	0.038
0	0.285	0.146	0.154	0.119	0.104	0.165	0.049

$Dis_{ext}(BF_4^-,0)$	Repeat 1					Repeat 2				
Number of **L**$^{\mathbf{A}}$	+7 charge	+6 charge	+5 charge	+4 charge	+3 charge	+7 charge	+6 charge	+5 charge	+4 charge	+3 charge
4	0.033	0.043	0.025	0.033	0.028	0.054	0.053	0.031	0.031	0.033
3	0.174	0.203	0.230	0.234	0.242	0.152	0.174	0.191	0.190	0.203
2	0.470	0.478	0.481	0.480	0.478	0.391	0.429	0.444	0.443	0.448
1	0.241	0.212	0.206	0.191	0.196	0.313	0.277	0.276	0.271	0.259
0	0.082	0.064	0.057	0.062	0.055	0.090	0.066	0.058	0.065	0.057

$Dis_{ext}(BF_4^-,0)$	Repeat 3					Combined	
Number of **L**$^{\mathbf{A}}$	+7 charge	+6 charge	+5 charge	+4 charge	+3 charge	Mean	ESD
4	0.061	0.089	0.025	0.031	0.025	0.040	0.017
3	0.119	0.139	0.138	0.131	0.141	0.177	0.039
2	0.333	0.404	0.457	0.459	0.469	0.444	0.040
1	0.361	0.291	0.311	0.297	0.299	0.267	0.048
0	0.127	0.077	0.069	0.083	0.066	0.072	0.018

$Dis_{ext}(I^-,1)$ + $Dis_{int}(I^-,1)$	Repeat 1					Repeat 2				
Number of **L**$^{\mathbf{A}}$	+7 charge	+6 charge	+5 charge	+4 charge	+3 charge	+7 charge	+6 charge	+5 charge	+4 charge	+3 charge
4	0.039	0.036	0.049	0.087	0.085	0.102	0.110	0.129	0.144	0.152
3	0.120	0.124	0.158	0.236	0.244	0.145	0.161	0.187	0.247	0.237
2	0.267	0.288	0.316	0.311	0.329	0.291	0.310	0.334	0.300	0.295
1	0.418	0.408	0.364	0.285	0.271	0.344	0.308	0.267	0.208	0.208
0	0.157	0.143	0.113	0.081	0.072	0.117	0.110	0.083	0.101	0.108

$Dis_{ext}(I^-,1)$ + $Dis_{int}(I^-,1)$	Repeat 3					Combined	
Number of **L**$^{\mathbf{A}}$	+7 charge	+6 charge	+5 charge	+4 charge	+3 charge	Mean	ESD
4	0.057	0.058	0.077	0.114	0.115	0.090	0.036
3	0.147	0.165	0.190	0.255	0.251	0.191	0.048
2	0.333	0.338	0.371	0.339	0.344	0.318	0.026
1	0.357	0.339	0.287	0.218	0.208	0.299	0.069

0	0.106	0.100	0.075	0.075	0.082	0.101	0.024

$Dis_{ext}(I^-,0)$	Repeat 1					Repeat 2				
Number of **L^A**	+7 charge	+6 charge	+5 charge	+4 charge	+3 charge	+7 charge	+6 charge	+5 charge	+4 charge	+3 charge
4	0.074	0.104	0.098	0.109	0.114	0.061	0.093	0.089	0.108	0.109
3	0.282	0.300	0.329	0.327	0.346	0.266	0.286	0.311	0.294	0.331
2	0.403	0.400	0.387	0.384	0.374	0.463	0.450	0.424	0.408	0.392
1	0.205	0.170	0.164	0.158	0.146	0.176	0.143	0.144	0.152	0.128
0	0.037	0.025	0.021	0.022	0.020	0.034	0.028	0.032	0.038	0.039

$Dis_{ext}(I^-,0)$	Repeat 3					Combined	
Number of **L^A**	+7 charge	+6 charge	+5 charge	+4 charge	+3 charge	Mean	ESD
4	0.066	0.106	0.087	0.118	0.110	0.096	0.017
3	0.254	0.267	0.300	0.284	0.318	0.300	0.026
2	0.454	0.450	0.424	0.407	0.399	0.415	0.027
1	0.186	0.147	0.153	0.155	0.134	0.157	0.019
0	0.040	0.030	0.036	0.036	0.038	0.032	0.007

$Dis_{ext}(Br^-,1)$ + $Dis_{int}(Br^-,1)$	Repeat 1					Repeat 2				
Number of **L^A**	+7 charge	+6 charge	+5 charge	+4 charge	+3 charge	+7 charge	+6 charge	+5 charge	+4 charge	+3 charge
4	0.011	0.017	0.026	0.043	0.045	0.039	0.044	0.061	0.074	0.075
3	0.035	0.036	0.051	0.067	0.069	0.070	0.069	0.104	0.103	0.103
2	0.250	0.171	0.200	0.200	0.201	0.123	0.192	0.251	0.242	0.239
1	0.485	0.536	0.503	0.486	0.480	0.558	0.481	0.428	0.411	0.401
0	0.218	0.240	0.219	0.204	0.205	0.210	0.214	0.156	0.171	0.182

$Dis_{ext}(Br^-,1)$ + $Dis_{int}(Br^-,1)$	Repeat 3					Combined	
Number of **L^A**	+7 charge	+6 charge	+5 charge	+4 charge	+3 charge	Mean	ESD
4	0.026	0.037	0.044	0.072	0.069	0.046	0.020
3	0.060	0.055	0.092	0.100	0.103	0.074	0.024
2	0.119	0.185	0.245	0.243	0.244	0.207	0.043
1	0.583	0.510	0.462	0.424	0.417	0.478	0.053
0	0.211	0.213	0.157	0.162	0.167	0.195	0.026

$Dis_{ext}(Br^-,0)$	Repeat 1					Repeat 2				
Number of **L^A**	+7 charge	+6 charge	+5 charge	+4 charge	+3 charge	+7 charge	+6 charge	+5 charge	+4 charge	+3 charge
4	0.082	0.092	0.102	0.086	0.108	0.067	0.070	0.073	0.084	0.087
3	0.242	0.263	0.297	0.297	0.306	0.221	0.241	0.272	0.263	0.289
2	0.402	0.404	0.388	0.398	0.386	0.435	0.440	0.424	0.406	0.419
1	0.232	0.206	0.187	0.189	0.176	0.244	0.211	0.199	0.202	0.179

0	0.043	0.035	0.027	0.029	0.024	0.032	0.037	0.032	0.045	0.026
$Dis_{ext}(Br^{-},0)$	Repeat 3					Combined				
Number of **L**^{A}	+7 charge	+6 charge	+5 charge	+4 charge	+3 charge	Mean	ESD			
4	0.057	0.066	0.069	0.077	0.083	0.080	0.013			
3	0.206	0.244	0.281	0.264	0.294	0.265	0.029			
2	0.450	0.445	0.426	0.405	0.414	0.416	0.020			
1	0.253	0.208	0.197	0.207	0.182	0.205	0.022			
0	0.033	0.038	0.027	0.047	0.028	0.034	0.007			

Dis$_{\mathbf{ext}}$(X^{-},1) is made of the same cages, as **Dis**$_{\mathbf{ext}}$(X^{-},0) (empty cages Co^{II}_{4}**L**$^{A}_{x}$**L**$^{B}_{4\text{-}x}$) and only differ by the number of anions X^{-} externally associated. The number of externally associated anions observed is only dependent on the ionisation of the mass spectrometer. Therefore the ratio of congeners within each clusters are identical and the distributions only differ by a scaling factor. As **1** can not encapsulate any anions (as observed in the NMR, Figure S18, S19 and S20), the peak corresponding to this cage (marked by x = 4 on the yellow triangle cluster in Figures S43, S44, S46, S47) is representative of **Dis**$_{\mathbf{ext}}$(X^{-},1) exclusively. The scaling factor between **Dis**$_{\mathbf{ext}}$(X^{-},1) and **Dis**$_{\mathbf{ext}}$(X^{-},0) could thus be obtained by comparing the integrals of the peak for **1** in the two adjacent cluster (Figures S43, S44, S46, S47, yellow triangle and green circle). It was thus possible to obtain the values of the integrals for the congeners within **Dis**$_{\mathbf{int}}$(X^{-},1) by subtracting **Dis**$_{\mathbf{ext}}$(X^{-},1) from the values observed in the cluster incorporating both of these distributions (Figures S43, S44, S46, S47, yellow triangle).

The values obtained for the congeners within **Dis**$_{\mathbf{int}}$(X^{-},1) state were normalized to unity and averaged across all charge states observed. The distributions obtained deviated strongly from the near binomial distribution observed for **Lib**$_{\mathbf{NTf_2}}$. Structures incorporating high numbers of **L**$^{\mathbf{B}}$ were favoured for all anions tested, with greater deviations for smaller anions. This correlated with the trends observed for binding of the anions in **2**, ie. $Br^{-} > I^{-} >> BF_4^{-} \geq ClO_4$.

Table S6. Normalised integrals for **Lib**$_{\mathbf{ClO_4}}$, **Lib**$_{\mathbf{BF_4}}$, **Lib**$_{\mathbf{I}}$ and **Lib**$_{\mathbf{Br}}$. Average and standard deviation over three repeats of the experiment and across all charge states.

**Integrals**	**Templating anion**	**ClO_4^{-}**		**BF_4^{-}**		**I^{-}**		**Br^{-}**	
**Number of L^{A}**		Average	SD	Average	SD	Average	SD	Average	SD
	0	0.161	0.096	0.296	0.072	0.279	0.054	0.307	0.029
	1	0.129	0.092	0.088	0.091	0.033	0.042	0.008	0.021
	2	0.220	0.154	0.110	0.130	0.086	0.093	0.062	0.064
	3	0.411	0.169	0.360	0.162	0.451	0.101	0.519	0.039
	4	0.078	0.082	0.146	0.131	0.151	0.109	0.104	0.056

The distributions obtained for **Lib**$_{\mathbf{NTf_2}}$, **Lib**$_{\mathbf{ClO_4}}$, **Lib**$_{\mathbf{BF_4}}$, **Lib**$_{\mathbf{I}}$ and **Lib**$_{\mathbf{Br}}$ were compared to the corresponding binomial distributions, and the scaling factors by which each species was amplified were calculated. For values below 1, which indicate a decrease in the expected amount of a congener, the inverse was calculated and given as a negative value to represent the decrease. The amplification or decrease was calculated by subtracting 1 to the absolute values, multiplying by 100, and rounding to two decimal places. The values are given in Table S7 below.

Table S7. Scaling factors and percentage amplification calculated for $\mathbf{Lib_{NTf_2}}$, $\mathbf{Lib_{ClO_4}}$, $\mathbf{Lib_{BF_4}}$, $\mathbf{Lib_I}$ and $\mathbf{Lib_{Br}}$ based upon the statistically-predicted binomial distribution.

Scaling factor (Percentage Amplification)	Number of L^A	Anion present				
		NTf_2^-	ClO_4^-	BF_4^-	I^-	Br^-
	0	0.87 (-16%)	2.58 (160%)	4.74 (370%)	4.4 (350%)	4.91 (390%)
	1	0.83 (-20%)	1.64 (64%)	1.44 (44%)	1.81 (81%)	2.08 (110%)
	2	1.05 (5%)	0.59 (-70%)	0.29 (-240%)	0.23 (-340%)	0.16 (-510%)
	3	1.11 (11%)	0.52 (-94%)	0.35 (-180%)	0.13 (-650%)	0.03 (-2900%)
	4	1.08 (8%)	1.25 (25%)	2.34 (130%)	2.41 (140%)	1.66 (66%)

The distributions obtained were also compared to the statistical distribution obtained previously from the library in presence of NTf_2^- ($\mathbf{Lib_{NTf_2}}$). Each type of structure was identified by the number of $\mathbf{L^A}$ ligands they contain. The relative energy of each could be determined by using the same formula as previously:

$$\Delta E_{rN} = -RTln(K_N), \qquad with\ K_N = \frac{\left(\frac{I_N}{I_{statN}}\right)}{I_0}$$

where I_N represented the normalized integrals of the MS peak for the structure incorporating N $\mathbf{L^A}$, I_{statN} was the corresponding normalized integral for the structure incorporating N $\mathbf{L^A}$ when no template was used (NTf_2^- only), and I_0 was the normalized integral of the MS peak for the original structure, here **2** (N = 0). Once averaged across charge state and across the three repeats for each experiment, the following values were obtained for libraries:

Table S8. Relative energies (kJ.mol^{-1}) of $\mathbf{Lib_{ClO_4}}$, $\mathbf{Lib_{BF_4}}$, $\mathbf{Lib_I}$ and $\mathbf{Lib_{Br}}$. Average over three repeats of the experiment and across all charge states.

	Templating anion	ClO_4^-	BF_4^-	I^-	Br^-
Number of L^A	0	0.00	0.00	0.00	0.00
	1	1.02	2.86	2.14	2.04
	2	4.15	7.39	7.83	8.90
	3	4.60	7.04	9.30	13.01
	4	2.33	2.29	2.07	3.22

8) Time dependency of ESI-MS response factors

In the studies of all libraries, the ESI-MS spectra used to determine the relative energies of the different cage species were obtained by integration of ESI-MS traces over 40 scans. For this method to be valid, the relative intensities of signals for each congener within the library need to be time-independent, *i.e.* signal intensities must not vary based on the time of acquisition. We checked this hypothesis by integrating the chromatogram of $\mathbf{Lib_{NTf_2}}$ over 3 short periods (scans 1 to 6, 18 to 23 and 35 to 40) as well as over the whole range (Scans 1 to 40).

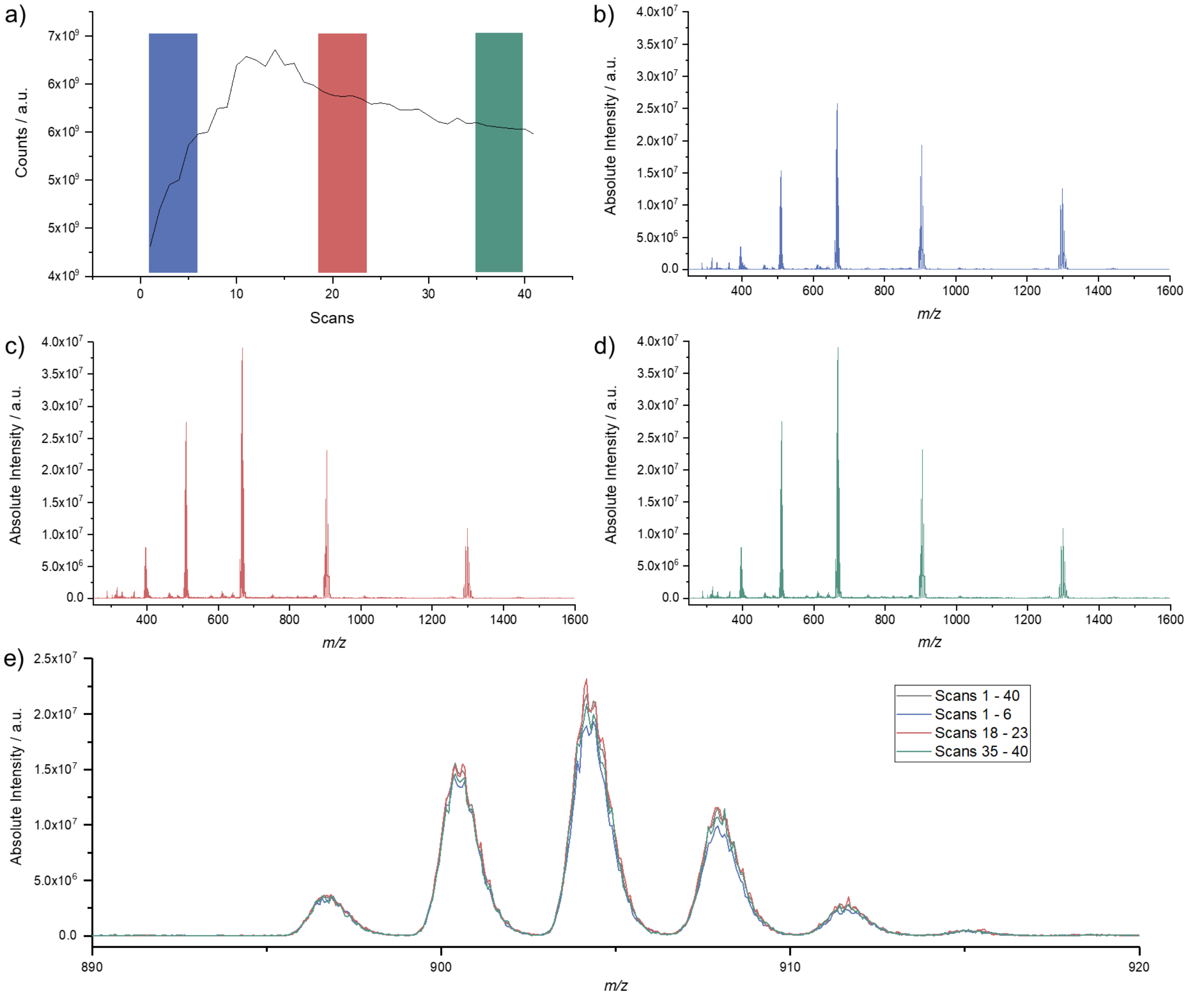

Figure S51. a) Total-ion chromatogram obtained for **Lib**$_{NTf_2}$ by ESI-MS. The three colored rectangles demarcate the regions over which the spectra given in b-d were obtained. b, c and d) Low-Resolution ESI-Mass Spectra of **Lib**$_{NTf_2}$ integrated over scans 1 – 6 (blue), 18 – 23 (red) and 35 – 40 (green) respectively. e) Zoom on the +4 charge state of the overlaid spectra.

The intensity of the chromatogram varied over time (Figure S51a). The lower initial intensity is a consequence of the presence of solvent in the tube, which needs to be displaced first. The slight subsequent decrease is a result of the gradual blockage of the filter and the cone over time caused by the high concentration of the sample required. This led to small differences in the absolute intensities for the spectra averaged over different time periods (Figure S51b, c and d). Despite these small variations in absolute intensities, the ratio of species within each charge state was not observed to vary between spectra obtained by integrating over different periods (Figure S51e). This conclusion was confirmed by integrating the signals for each cage species in the library between the four spectra in each charge state. The absolute values of the integrals obtained are given in Table S9, top part. In order to compare the integrals within charge state and within the different scans, the values of absolute integrals were normalised (Table S9, middle section). The values were averaged across all charge state observed (Table S9, bottom section) and plotted as a function of the number of ligand **L**$^{\mathbf{A}}$ for each spectrum (Figure S52).

Table S9. Absolute integrals (top), normalized integrals (middle) and averaged values (bottom) across the charge states of the congeners of **Lib**$_{NTf_2}$ for spectra averaged over scans 1 – 40, 1 – 6, 18 – 23 and 35 – 40.

Absolute Integrals	+7 charge				+6 charge				+5 charge			
Number **L**^{A}	Range 1-40	Range 1-6	Range 18-23	Range 35-40	Range 1-40	Range 1-6	Range 18-23	Range 35-40	Range 1-40	Range 1-6	Range 18-23	Range 35-40
4	7.2E+05	3.8E+05	8.0E+05	7.8E+05	2.7E+06	1.8E+06	2.9E+06	2.8E+06	5.7E+06	4.2E+06	5.9E+06	5.7E+06
3	2.9E+06	1.4E+06	3.2E+06	3.3E+06	1.2E+07	7.6E+06	1.3E+07	1.2E+07	2.4E+07	1.7E+07	2.5E+07	2.4E+07
2	4.7E+06	2.3E+06	5.4E+06	5.2E+06	2.0E+07	1.3E+07	2.1E+07	2.0E+07	3.7E+07	2.6E+07	3.9E+07	3.7E+07
1	3.0E+06	1.5E+06	3.3E+06	3.2E+06	1.1E+07	7.2E+06	1.2E+07	1.2E+07	2.0E+07	1.4E+07	2.2E+07	2.1E+07
0	1.3E+06	8.5E+05	1.4E+06	1.4E+06	3.2E+06	2.0E+06	3.6E+06	3.3E+06	5.4E+06	3.4E+06	5.9E+06	5.6E+06
	+4 charge				+3 charge							
Number **L**^{A}	Range 1-40	Range 1-6	Range 18-23	Range 35-40	Range 1-40	Range 1-6	Range 18-23	Range 35-40				
4	4.6E+06	4.3E+06	4.5E+06	4.3E+06	3.6E+06	4.3E+06	3.4E+06	3.2E+06				
3	1.9E+07	1.8E+07	1.9E+07	1.8E+07	1.4E+07	1.7E+07	1.3E+07	1.3E+07				
2	2.7E+07	2.4E+07	2.8E+07	2.6E+07	1.9E+07	2.2E+07	1.8E+07	1.7E+07				
1	1.5E+07	1.2E+07	1.5E+07	1.4E+07	9.6E+06	1.0E+07	9.3E+06	8.7E+06				
0	3.8E+06	3.3E+06	3.9E+06	3.6E+06	2.5E+06	2.7E+06	2.4E+06	2.2E+06				

Normalised Integrals	+7 charge				+6 charge				+5 charge			
Number **L**^{A}	Range 1-40	Range 1-6	Range 18-23	Range 35-40	Range 1-40	Range 1-6	Range 18-23	Range 35-40	Range 1-40	Range 1-6	Range 18-23	Range 35-40
4	0.057	0.059	0.056	0.056	0.055	0.058	0.055	0.055	0.061	0.065	0.060	0.061
3	0.228	0.221	0.227	0.235	0.245	0.245	0.246	0.247	0.263	0.265	0.255	0.261
2	0.374	0.353	0.382	0.377	0.401	0.402	0.403	0.398	0.398	0.406	0.399	0.397
1	0.237	0.234	0.234	0.230	0.233	0.232	0.229	0.235	0.220	0.211	0.226	0.222
0	0.104	0.133	0.101	0.102	0.065	0.063	0.068	0.065	0.058	0.053	0.060	0.059
	+4 charge				+3 charge							
Number **L**^{A}	Range 1-40	Range 1-6	Range 18-23	Range 35-40	Range 1-40	Range 1-6	Range 18-23	Range 35-40				
4	0.065	0.069	0.064	0.065	0.074	0.075	0.073	0.073				
3	0.278	0.291	0.274	0.277	0.290	0.303	0.287	0.286				
2	0.394	0.389	0.399	0.395	0.391	0.389	0.388	0.391				
1	0.208	0.200	0.208	0.209	0.195	0.185	0.201	0.198				
0	0.054	0.052	0.055	0.054	0.050	0.048	0.051	0.051				

Normalised Integrals	Mean				ESD			
Number **L**^{A}	Range 1-40	Range 1-6	Range 18-23	Range 35-40	Range 1-40	Range 1-6	Range 18-23	Range 35-40
4	0.062	0.065	0.062	0.062	0.024	0.025	0.024	0.024
3	0.261	0.265	0.258	0.261	0.099	0.102	0.098	0.099
2	0.391	0.388	0.394	0.392	0.146	0.146	0.147	0.146
1	0.219	0.212	0.220	0.219	0.083	0.081	0.083	0.083
0	0.066	0.070	0.067	0.066	0.031	0.039	0.030	0.030

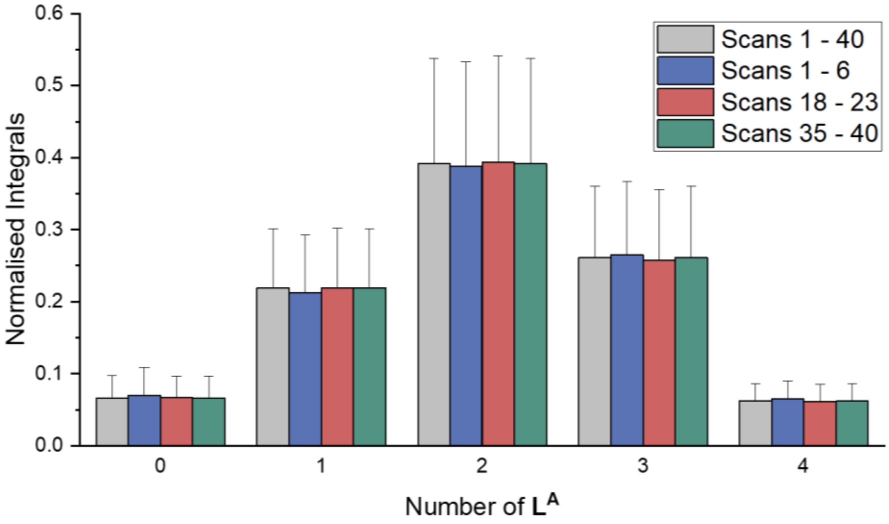

Figure S52. Normalized integrals for the spectra averaged over different scans. The values were averaged across all charge state to obtain standard deviations, which are shown as positive error bars for clarity.

Small discrepancies were observed between charge states within a single spectrum. However once averaged over all charge states, no significant differences were observed between the values obtained for the spectra integrated over different scans.

This additional evidence, along with the similar response factor of cage **1** and **2** (Section S7.1) and the agreement between NMR and ESI-MS data (Section S7.2), confirms that quantitative information on the amount of complexes present in solution could be extracted from the ESI-MS data obtained in this study.

References

[1] I. A. Riddell, M. M. J. Smulders, J. K. Clegg, Y. R. Hristova, B. Breiner, J. D. Thoburn, J. R. Nitschke, *Nat. Chem.* **2012**, *4*, 751-756.

[2] C. S. Wood, T. K. Ronson, A. M. Belenguer, J. J. Holstein, J. R. Nitschke, *Nature Chemistry* **2015**, *7*, 354.

[3] *COLLECT*, Nonius BV: Delft, The Netherlands, **1998**.

[4] *CrystalClear*, 2.0; Rigaku Americas and Rigaku Corporation.: 9009 TX, USA **1997-2009**.

[5] S. J. Coles, P. A. Gale, *Chem. Sci.* **2012**, *3*, 683-689.

[6] Z. Otwinowski, W. Minor, *Methods in Enzymology* **1997**, *276*, 307-326.

[7] R. H. Blessing, *Acta Crystallographica Section A: Foundations* **1995**, *A51*, 33-38.

[8] L. J. Farrugia, *J. Appl. Cryst.* **1999**, *32*, 837-838.

[9] L. Palatinus, G. Chapuis, *J. Appl. Crystallogr.* **2007**, *40*, 786-790.

[10] G. M. Sheldrick, *SHELX-97: Programs for Crystal Structure Analysis*, University of Göttingen, Göttingen, **1997**.

[11] P. van der Sluis, A. L. Spek, *Acta Crystallographica Section A* **1990**, *46*, 194-201.

[12] A. L. Spek, *PLATON: A Multipurpose Crystallographic Tool*, Utrecht University, Utrecht, The Netherlands, **2008**.

[13] G. J. Kleywegt, T. A. Jones, *Acta Crystallographica Section D* **1994**, *50*, 178-185.

[14] S. Mecozzi, J. Rebek, Julius, *Chem.: Eur. J.* **1998**, *4*, 1016-1022.

[15] W. Meng, J. K. Clegg, J. D. Thoburn, J. R. Nitschke, *J. Am. Chem. Soc.* **2011**, *133*, 13652-13660.

[16] Jaynes, E. T., *Am. J. Phys*. **1965**, *33*, 391-398.

Author Contributions

MK synthesized and characterized the cages and libraries (lead), carried out the binding studies (lead), designed and carried out the TBA competition assays (lead), contributed to the modelling of the anion binding (support), designed and executed the MS experiments for the analysis of library composition and carried out the energetic calculations (lead).

RAB carried out initial characterization and testing on the library redistribution (initial testing). RAB grew the crystals for cage **1** and **2** (lead).

JDT designed the Mathematica notebook used to calculate the binding constants and model anion binding (lead).

JKC resolved the crystal structures of the cages (lead).

The original draft was written and figures were done by MK. RAB, JDT, JKC and JRN all contributed to the manuscript at later stages.

JRN administered and secured funding for the project.
